# Photocontrolled Reversible Amyloid Fibril Formation of Parathyroid Hormone-Derived Peptides

**DOI:** 10.1021/acs.bioconjchem.4c00188

**Published:** 2024-06-12

**Authors:** André Paschold, Moritz Schäffler, Xincheng Miao, Luis Gardon, Stephanie Krüger, Henrike Heise, Merle I. S. Röhr, Maria Ott, Birgit Strodel, Wolfgang H. Binder

**Affiliations:** † Macromolecular Chemistry, Institute of Chemistry, Faculty of Natural Science II, Martin Luther University Halle Wittenberg, von-Danckelmann-Platz 4, Halle 06120, Germany; ‡ Institute of Theoretical and Computational Chemistry, Heinrich Heine University Düsseldorf, Düsseldorf 40225, Germany; § Institute of Biological Information Processing, Structural Biochemistry (IBI-7), Forschungszentrum Jülich, Jülich 52425, Germany; ∥ Center for Nanosystems Chemistry (CNC), Theodor-Boveri Weg, Universität Würzburg, Würzburg 97074, Germany; ⊥ Institut für Physikalische Biologie, Heinrich-Heine-Universität Düsseldorf, 40225 Düsseldorf, Germany; # Biozentrum, Martin Luther University Halle-Wittenberg, Weinberweg 22, Halle 06120, Germany; ∇ Institute of Biophysics, Faculty of Natural Science I, Martin Luther University Halle-Wittenberg, Kurt-Mothes-Straße 3, Halle 06120, Germany

## Abstract

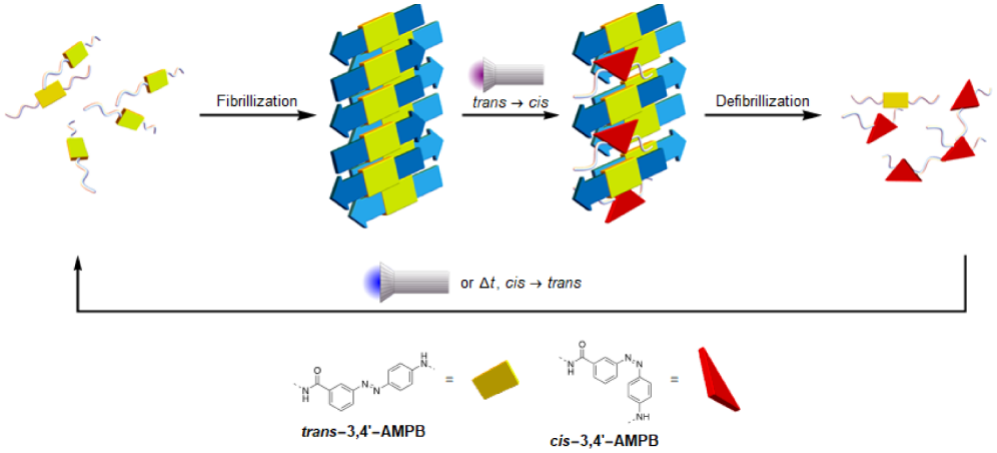

Peptide fibrillization is crucial in biological processes such as amyloid-related diseases and hormone storage, involving complex transitions between folded, unfolded, and aggregated states. We here employ light to induce reversible transitions between aggregated and nonaggregated states of a peptide, linked to the parathyroid hormone (PTH). The artificial light-switch 3-{[(4-aminomethyl)phenyl]diazenyl}benzoic acid (AMPB) is embedded into a segment of PTH, the peptide PTH_25–37_, to control aggregation, revealing position-dependent effects. Through *in silico* design, synthesis, and experimental validation of 11 novel PTH_25–37_-derived peptides, we predict and confirm the amyloid-forming capabilities of the AMPB-containing peptides. Quantum-chemical studies shed light on the photoswitching mechanism. Solid-state NMR studies suggest that β-strands are aligned parallel in fibrils of PTH_25–37_, while in one of the AMPB-containing peptides, β-strands are antiparallel. Simulations further highlight the significance of π–π interactions in the latter. This multifaceted approach enabled the identification of a peptide that can undergo repeated phototriggered transitions between fibrillated and defibrillated states, as demonstrated by different spectroscopic techniques. With this strategy, we unlock the potential to manipulate PTH to reversibly switch between active and inactive aggregated states, representing the first observation of a photostimulus-responsive hormone.

## Introduction

**Figure 1:**
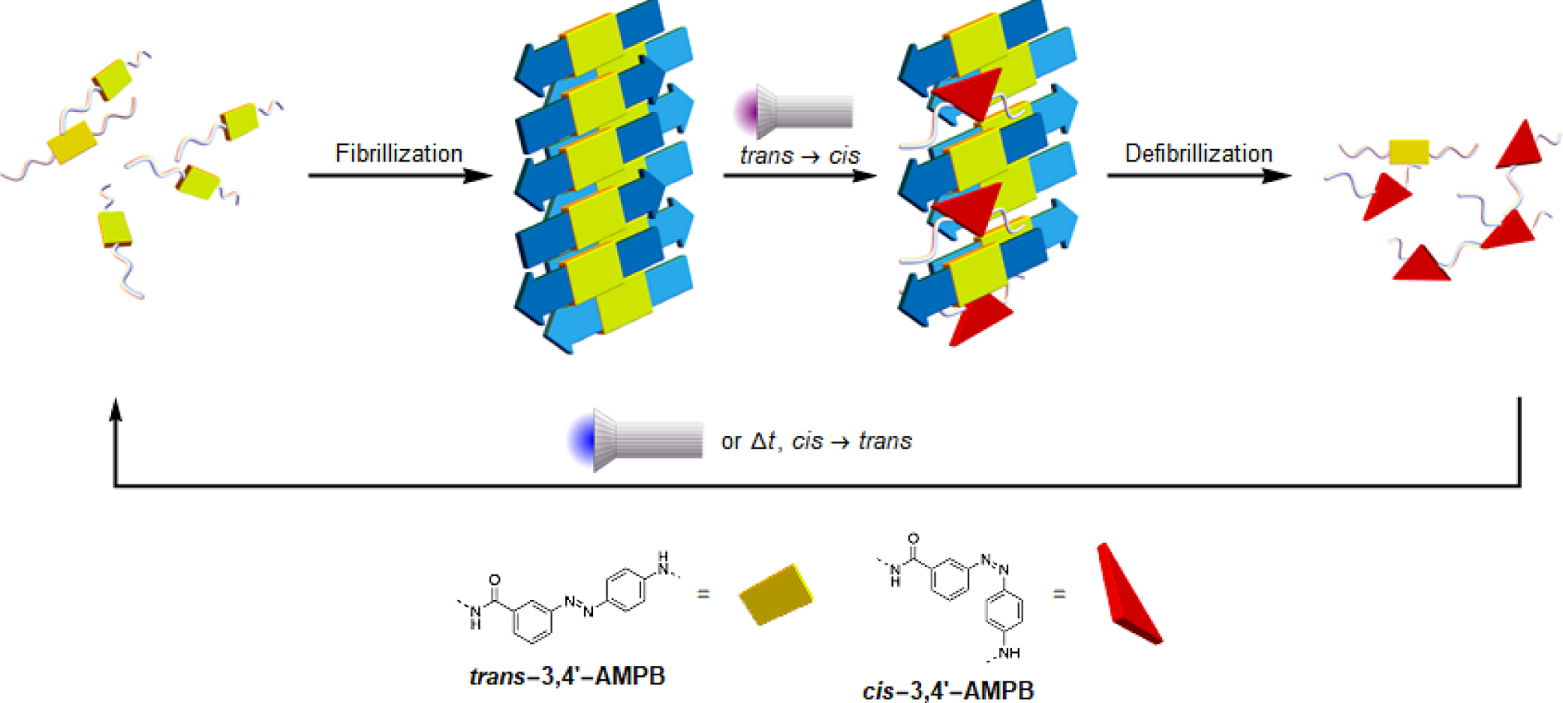
Concept for a light-driven (de)-aggregation of the parathyroid-hormone (PTH). The artificial light-switch, 3-{[(4-aminomethyl)phenyl]diazenyl}benzoic acid (*cis*/*trans*-3,4′-AMPB) is embedded at various positions of a peptide-fragment of PTH, switching between the *cis*/*trans*-form, in this mode regulating the reversible assembly of the peptide into fibrils.

Modulating a protein’s secondary structure stands as a pivotal strategy to define and harness its functionality.^1^ While numerous protein structures are identified, predicted, and engineered, the concept of inducing conformational changes by external triggers to alter their biological activities remains rare. Temperature,^2^ pH,^3^ polarity,^4^ or light offer avenues for such a dynamic control, in particular when applied to sensitive functional groups inserted inside the protein. Among these, light emerges as a particularly advantageous stimulus, providing precise temporal control across vast time scales, noninvasiveness, and compatibility with intricate matrices like living tissues.^5^ Leveraging light-induced conformational changes has demonstrated success in various proteins, from transporter-proteins like rhodopsins to enzymes, showcasing potential applications in photopharmacology and diverse enzymatic processes.^5,6^ Moreover, external photoswitches have successfully enabled photomodulation in a diverse array of enzymes,^7^ modifying binding affinities,^8^ facilitating peptide purification through photoaffinity,^9^ and controlling secondary structure alterations.^10^ Beyond the structure of individual peptides or proteins, there is a strong interplay between secondary structure and aggregation of peptides. This process is particularly pronounced in the context of amyloid aggregation, where proteins form β-sheet-rich structures, leading to the formation of highly ordered fibrillar aggregates.^11^ Such fibrils are a hallmark of several neurodegenerative diseases, including Alzheimer’s and Parkinson’s. Understanding and controlling amyloid aggregation can thus be crucial for developing therapies for diseases associated with these pathological protein assemblies. However, controlling peptide aggregation proves to be a significant challenge, given the complex processes involved. This challenge arises from intricate intrapeptide and interpeptide interactions, coupled with extensive conformational changes on a large scale, particularly when considering the regulation of primary and secondary nucleation preceding fibrillization. Therefore, the utilization of light to control amyloid assembly processes represents a groundbreaking advancement, enabling control over the bioavailability of hormones, such as the parathyroid hormone (PTH). PTH is reversibly stored in functional amyloid fibrils, as these fibrils, unlike, e.g., amyloid-Aβ fibrils, can disintegrate again after aggregation. In this study, we pioneer the utilization of light to achieve a reversible transition between the aggregated and nonaggregated states of PTH (Figure 1), allowing the regeneration of PTH fibrils through precise light control, presenting a transformative advancement in the field.^12^

PTH, a reversibly fibrillating 84-amino acid hormone, is ubiquitously distributed in animals.^13^ Responsible for regulating calcium and phosphate homeostasis,^14^ mature PTH_1–84_ is stored in functional amyloids before secretion,^15^ wherein its N-terminal pro-sequence potentially prevents premature aggregation.^16^ PTH-related diseases prompt the use of approved drugs, Natpara (PTH_1–84_) and Forteo (PTH_1–34_), addressing hormone imbalances.^17^ While the physiological role of the N-terminal 34 amino acids of PTH_1–84_ is well investigated, being crucial for the activation of G-protein coupled receptors of bone and kidney cells^18^ and in the nervous system for calcium and phosphate homeostasis,^19^ there remains a knowledge gap regarding the fibrillization process and the resulting fibrils. Current knowledge indicates that under physiological conditions, the thermodynamic stability of PTH_1–84_ fibrils is low enough to allow dissociation upon dilution, with the fibril-forming sequence encompassing amino acids R25-L37.^15^ Previous research explored the impact of the pro-sequence^16^ and environmental factors on the fibrillization process^20,21^ of PTH, however, with an only limited insight into the precise structural parameters controlling assembly and disassembly of the fibrils.^22^ The aim of the current work is to gain deeper insights into the fibrillization of PTH_25–37_, coupled with the ability to reversibly control this process and understand the structural principles of the assembly process. We employ a synthetic approach for synthesizing PTH_25–37_ peptides bearing the azobenzene photoswitch, 3-{[(4-aminomethyl)phenyl]diazenyl}benzoic acid (AMPB), guided by bioinformatics to strategically place AMPB for optimal photocontrol, with biophysical techniques such as thioflavin T (ThT) fluorescence, CD spectroscopy, and transmission electron microscopy (TEM) to assess the peptide aggregation dynamics and aggregate morphology. This is further combined with molecular dynamics (MD) simulations to elucidate the impact of AMPB on the structure and dynamics of the designed peptides and their small oligomers (amounting to a total of 285 μs simulation time), while wide-angle X-ray scattering (WAXS) and solid-state nuclear magnetic resonance (ssNMR) spectroscopy in conjunction with MD are employed to provide structure models for selected amyloid fibrils. Finally, quantum-chemical potential energy scans of both the ground (S_0_) and excited states (S_1_ and T_1_) reveal insights into the photoswitching mechanism of the AMPB group and its electronic interaction when integrated into PTH_25–37_, considering both the monomeric and dimeric peptide state. Providing a thorough understanding of the aggregation behavior of PTH_25–37_ and its derivatives, we have engineered a peptide analogue with the unique capability of reversible light-induced switching of its aggregation state.

## Results and Discussion

### Aggregation Characteristics of Unmodified PTH_25–37_ and Engineered Peptide

**Figure 2:**
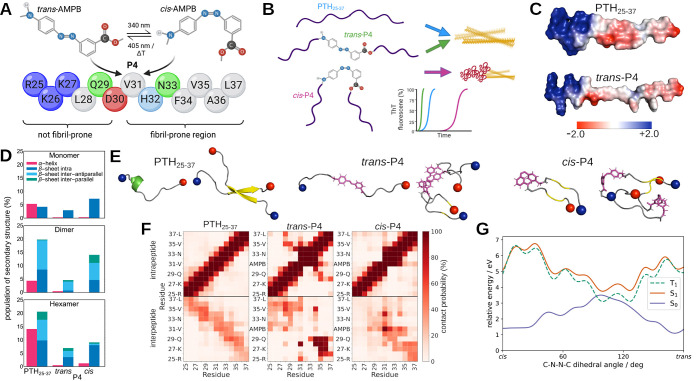
(A) Sequence of PTH_25–37_, where the fibril-forming region identified by bioinformatic analysis is labeled, and the chemical structure of AMPB, which replaces V31 in P4, is shown. The side chains of the amino acids, shown in blue and red, are positively and negatively charged at pH 7.4, respectively, with H32 shown in light blue as a borderline case, while green and gray indicate polar and hydrophobic amino acids, respectively. (B) PTH_25–37_ and *trans*-P4 form fibrils, as shown in the ThT fluorescence cartoon below (blue and green line), while *cis*-P4 initially forms amorphous aggregates, which then transform into fibrils (red line in ThT cartoon). (C) Electrostatic potentials surface (values in kTe^–1^ according to the color scale at the bottom) of PTH_25–37_ and *trans*-P4. (D) Average simulated secondary structure population, divided into α-helical, intrapeptide β-sheets, interpeptide parallel, and antiparallel β-sheets as indicated by the color code. Results are shown for simulations of monomers, dimers, and hexamers of PTH_25–37_, *trans*-P4, and *cis*-P4. (E) Representative snapshots of the monomer and dimer simulations, with the α-helix shown in green, the β-sheets in yellow, the random coil in gray, the N- and C-termini as blue and red spheres, respectively, and AMPB in purple. (F) Contact matrix of dimer simulations for PTH_25–37_, *trans*-P4, and *cis*-P4. (G) Quantum chemical potential energy scan of the *trans* → *cis* isomerization of the AMPB photoswitch in P4.

To understand the aggregation of PTH_25–37_ and make informed design decisions, we began our analysis with a detailed examination of the original peptide PTH_25–37_ and one engineered peptide (P4),^22^ bearing the photoswitch in the central part of the peptide (Figure 2A). Unmodified PTH_25–37_ and *trans*-P4 form amyloid fibrils within 15 and 10 h, respectively, whereas *cis*-P4 initially forms amorphous aggregates and only forms amyloid fibrils after about 50 h (Figure 2B).^22^ The increased rate of aggregation of *trans*-P4 compared to PTH_25–37_ is assigned to the physicochemical properties of the amino acids and the phototrigger, AMPB, that make up the peptide (Figure 2A). PTH_25–37_ has three positive charges (RKK) at the N-terminus, followed by a mixture of hydrophobic, polar and one negatively charged residue (D30) in the middle, while the C-terminal residues are mainly hydrophobic. This uneven distribution of physicochemical properties across the sequence is also reflected in the electrostatic potential surface, which shows a strongly positively charged N-terminus and a more hydrophobic C-terminal half with some negative charge accumulation beyond the first three residues (Figure 2C). The assumption therefore is that amyloid aggregation of PTH_25–37_ is driven by the residues after the initial RKK sequence. To test this assumption, we used four aggregation predictors: PASTA,^23^ AGGRESCAN,^24^ AmyloGRAM,^25^ and FoldAmyloid,^26^ which show that the sequence ^32^HNFVA^37^L is an aggregation hotspot and that the first five amino acids ^25^RKKL^29^Q should not contribute to fibrillization. For ^30^DV, a low tendency to aggregation was observed. In P4, one of the residues of the later sequence, V31, is replaced by AMPB. This increases the overall hydrophobicity of the peptide, while the azo group itself adds some positive charge to the electrostatic potential, which compensates for the predominant negative charge in the C-terminal part of the peptide. These two effects, therefore, explain the faster aggregation kinetics of *trans*-P4 compared to PTH_25–37_.

To understand the aggregation mechanisms and elucidate the structures that form during aggregation, namely first oligomers and finally fibrils, we performed MD simulations and ssNMR spectroscopy of both PTH_25–37_ and P4, the latter simulated in the *trans*- and *cis*-states (see Table S1 for an overview of simulations performed). In this way, we can also develop a structural understanding of why the fibrillization of *cis*-P4 is slowed down and generally reduced. The monomers of either PTH_25–37_, *trans*-P4, and *cis*-P4 are mainly in a random coil state (Figure 2D). Nevertheless, small differences between the monomeric peptides can be observed. One of them is that PTH_25–37_ shows a certain tendency to form an α-helix in the N-terminal residues up to D30, which is lost when V31 is replaced by AMPB (Figure 2E). When the azo group is in the *cis*-state, this allows more intrapeptide contacts, including the formation of a β-hairpin, whereas in the *trans*-state, P4 is mostly in a fully elongated state. The dimer simulations showed an increase in β-sheets for all three peptides, but most for PTH_25–37_. This is a result of peptide aggregation, as there is a particular increase in β-sheets between the peptides (Figure 2D), which are mainly arranged antiparallel, as shown by the contact matrix (Figure 2F). In an antiparallel arrangement, the three positively charged N-terminal residues RKK can interact with the negative charge at the C-terminus of the neighboring peptide. Interestingly, the *trans*-P4 peptide, which we expected to aggregate the fastest based on the fibrillization data, forms fewer β-sheets at the oligomer level. Instead, the interpeptide interactions are dominated by contacts between the two AMPB groups, while the peptides are aligned antiparallel to each other. In contrast, although the *cis*-P4 peptide adopts β-sheet structures to some extent due to intrapeptide hairpins, it aggregates mainly randomly, as confirmed by the many interpeptide contacts, which is consistent with the amorphous aggregates observed *in vitro*. The distinct differences between peptide aggregation involving *cis*- and *trans*-AMPB allow fibril formation to be controlled at the molecular level by isomerization, providing a solid basis for photocontrol of amyloid formation.

In an effort to identify the nucleus of fibril formation, we also simulated the hexamer formation of PTH_25–37_, *trans*-P4, and *cis*-P4. However, this system size is still too small (or the simulation time too short) to observe the emergence of fibrillar structures. On the contrary, the hexamers of these peptides are less ordered than the dimers. This confirms the experimental observation that the propensity of PTH_25–37_ to form amyloid is much lower than that of other peptides, such as that of Aβ_16–22_. For the latter, we observe the formation of ordered hexamers when simulated under the same conditions as here,^27^ while ThT experiments for this peptide show that fibrils are already present at the beginning of the measurements.^28^ For both PTH_25–37_ and P4, we even see a decrease in interpeptide β-sheets when we increase the system size to the hexamer, which can be explained by the increase in dimensionality of the conformational space, which allows for more interpeptide interactions and makes it less likely to see ordered aggregates on short time scales. Another interesting observation is that the amount of helix formed increased in the hexamer system of the PTH_25–37_ compared to its monomer and dimer. This again reflects the helical propensity of this peptide, which can be stabilized by interpeptide interactions, a common observation in intrinsically disordered peptides that can fold after binding to interaction partners.^29^

We further investigated the electronic interactions and the photoswitching mechanism of AMPB integrated into PTH_25–37_, both in the monomer and dimer states. To this end, we generated 34 switching trajectories using MD simulations to model the *trans* → *cis* isomerization of the AMPB photoswitch along the CNNC dihedral angle. The resulting ensemble of pathways was individually analyzed using ONIOM-based QM/QM2^30^ (NEVPT2^31^/xTB^32^) calculations, yielding potential energy scans for the ground and first excited triplet and singlet states (Figures 2G and S1). While the calculations clearly indicate that photoswitching is feasible, we also found that several scans exhibit structural barriers, that may impact the fluorescence wavelength and lifetime compared to the pure photoswitch (Figure S2).^33^ Further elucidation through principal component analysis of the distance matrices between peptide residues (Figures S3 and S4) revealed critical structural motifs potentially responsible for the observed S_1_ barrier in *trans* → *cis* isomerization paths. These motifs particularly involve configurations of R25 interacting with A36/L37 and H32/Q33, in both monomeric and dimeric forms (Figure S5).

### Structures of PTH_25–37_ and P4 Fibrils

**Figure 3:**
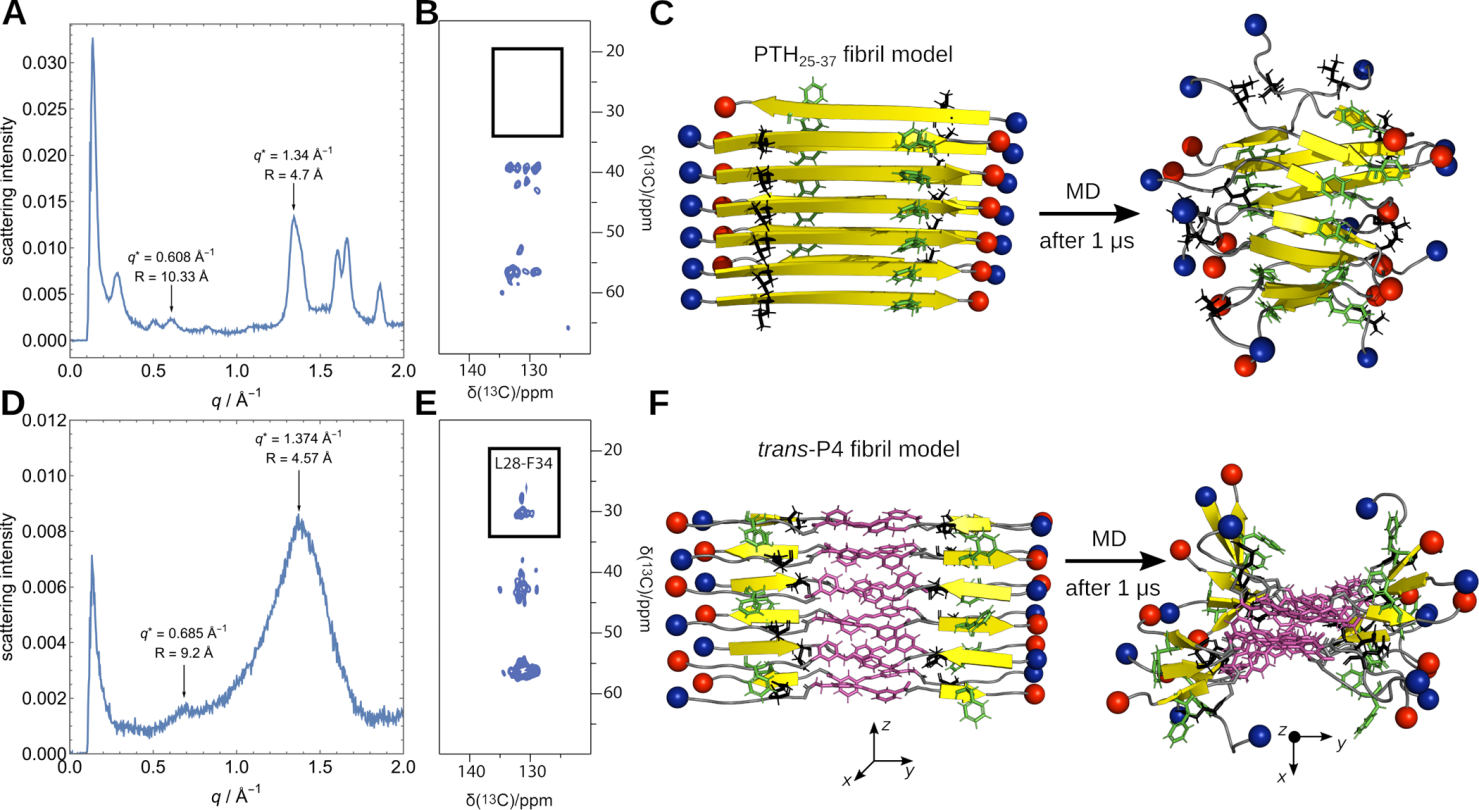
(A, D) WAXS results for PTH_25–37_ and the AMPB-modified PTH_25–37_ peptide *trans*-P4. (B, E) Solid-state NMR (ssNMR) 2D ^13^C–^13^C proton driven spin diffusion spectra close to rotational resonance (mixing time 1s) of fibrils of the PTH_25–37_ and *trans*-P4 peptide with uniformly ^13^C-labeled L28 and F34. (C, F) Fibril models of PTH_25–37_ and *trans*-P4 constructed based on the ssNMR (left) and at the end of MD simulations (right), where β-sheets are shown in yellow, random coil in gray, the N- and C-termini as blue and red spheres, respectively, AMPB in purple, and the side chains of L28 and F34 are highlighted as black and green sticks, respectively. Note that in panel F, the structure on the right-hand side is rotated by 90° to better represent the fibrillar arrangement.

The final state of aggregation, the amyloid fibrils, was characterized by wide-angle X-ray scattering (WAXS) and ssNMR, and the resulting data were used to generate structural models whose stability was evaluated in MD simulations. The WAXS measurement was performed with preformed fibrils of either PTH_25–37_ or *trans*-P4. Due to the isotropic orientations of the fibrils and the resulting isotropic scattering pattern (see Figure S6), the scattering intensities were angular averaged and are displayed in Figure 3 A, D. For PTH_25–37_, we observed a diffraction pattern typical for β-sheet-containing amyloid fibrils.^34^ The reflection at 4.7 Å indicates a structural repeat corresponding to the distance between two β-strands within a sheet, whereas the reflection at 10.3 Å corresponds to the distance between two β-sheets in a fibril. A reflection at 9.4 Å, which would correspond to the repeating unit of two antiparallel β-strands within a β-sheet (i.e., 2 × 4.7 Å), is not observed, suggesting a parallel alignment of the strands within the β-sheets, which allows the hydrophobic, aggregation-prone residues on the C-terminal side of the peptide to lie adjacent to each other. To test this conclusion, we performed ssNMR measurements of synthesized PTH_25–37_ with uniformly ^13^C-labeled L28 and F34. In the 2D ^13^C–^13^C spin diffusion measurements with longitudinal mixing times of 500 ms to 1 s, we did not observe any cross-peaks between these residues (Figures 3B, S7). This finding is indicative of a distance of >6 Å between these residues^35^ and thus supports parallel β-sheets in PTH_25–37_ fibrils. To answer the question of how two sheets of parallel stacked peptides could be arranged in the fibril,^36^ we constructed four possible fibril models (with 6 peptides per sheet) consistent with the NMR distance data and tested their stability in MD simulations. Only one of these arrangements proved to be stable, even after 1 μs MD. In this model, the β-sheets, which consist of parallel and in-register strands, are oriented antiparallel and their R25 side chains point inward (Figure 3C). This also agrees with our findings from the oligomer simulations, which revealed a preferred antiparallel arrangement between the PTH_25–37_ peptides, as this allows the positive charges at the N-terminus to interact with the negatively charged C-terminus. In the fibril, this is realized via intersheet interactions, while within the sheets, the hydrophobic residues are adjacent to each other for optimal β-sheet stability. The fibril model at the end of the simulation confirms that the β-sheets are stable. The β-conformation is partially lost only at the terminal residues, which is due to the electrostatic repulsion between the three positive charges on the RKK residues, which also cause twisting of the fibril. The simulation-averaged distance between β-strands is 4.8 Å, and between two sheets, it is 10.3 Å, in agreement with the WAXS data.

The WAXS signals for *trans*-P4 (Figure 3D) are much broader than for PTH_25–37_, indicating less structured fibrils for P4. Nevertheless, the peak for strand spacing at 4.6 Å dominates the signal, clearly indicating β-sheet formation. The next peak is at 9.2 Å, which corresponds to twice the interstrand distance. This indicates an antiparallel arrangement of the *trans*-P4 peptides within a sheet, as here the antiparallel unit consisting of two peptides is another repeat unit leading to a scattering signal. A signal for the intersheet distance is not visible, but could be hidden under the 9.2 Å signal, considering that the signal at 10.3 Å for the PTH_25–37_ fibrils is also not strong. The conclusion that the *trans*-P4 peptides are antiparallel in the fibrils is supported by the ssNMR measurements showing an L28-F34 cross-peak in 2D ^13^C–^13^C spin diffusion spectra, which is indicative of an inter-residual distance <6 Å (Figures 3E and S8). Based on this information and in addition to the results of our dimer and hexamer simulations of *trans*-P4, which revealed a strong interaction between the AMPB groups, we created several possible fibril structures and tested their stability in MD simulations. The structure that met all our experimental specifications and was also stable during the 1 μs simulation is the one shown in Figure 3F. The structure at the end of the simulation shows a larger deviation from the idealized fibril model and with shorter β-sheets than observed for PTH_25–37_, which explains the broader WAXS signal. Stabilizing aspects are that the oppositely charged N- and C-terminal ends are directly adjacent within and between the β-sheets and that the central AMPB groups are aligned parallel to each other for π–π stacking interactions. The antiparallel β-sheets themselves are less stable, however, as they are formed between the hydrophobic C-terminal half and the nonamyloid-prone N-terminal half on both sides of the AMBP group. Moreover, the AMPB group in the center of the peptide breaks the β-sheet structure, which explains that the P4 fibrils are shorter than those of PTH_25–37_. In addition, the π–π stacking and β-sheet stacking have opposite spacing requirements with ≈3.8 Å and ≈10 Å, respectively, which can be clearly seen in the MD snapshot shown in Figure 3F and further explains the broadness of the corresponding WAXS signal.

### Peptide Design

Based on all simulations and structural investigations made for PTH_25–37_ and the P4 variant, we designed 11 novel PTH_25–37_-derived peptides, wherein the amyloid-forming capabilities of the AMPB group in view of fibrillization were probed by placing the photoswitch at positions in the center of the peptide (P1–P6), at the N-terminal (P7–P9) or at the C-terminal part (P10–P12), with an amino acid either being exchanged by AMPB or AMPB being inserted between two amino acids (Table 1).

**Table 1:** Designed PTH_25–37_ Peptides Containing the AMPB Photoswitch (Azo) Inserted between Two Amino Acids (i) or an Amino Acid Exchanged by AMPB (e)^a^

	peptide	primary sequence	modification	*t*_**1/2**_**(***cis***) [h]**
	PTH_25–37_	^25^RKKLQ^30^DVHNF^35^VAL	-	-
	P1 (i)	^25^RKKLQ^30^D-Azo-VHNF^35^VAL	D30-Azo-V31	90
	P2 (i)	^25^RKKLQ^30^DV-Azo-HNF^35^VAL	V31-Azo-H32	90
central	P3 (e)	^25^RKKLQ-Azo-VHNF^35^VAL	D30 → Azo	-
	P4 (e)	^25^RKKLQ^30^D-Azo-HNF^35^VAL	V31 → Azo	97
	P5 (e)	^25^RKKLQ-Azo-HNF^35^VAL	D30,V31 → Azo	-
	P6 (i)	^25^RKKLQ-Azo-^30^DVHNF^35^VAL	Q29-Azo-D30	-
	P7 (i)	^25^RKKL-Azo-Q^30^DVHNF^35^VAL	L28-Azo-Q29	72
N-terminal	P8 (i)	^25^RK-Azo-KLQ^30^DVHNF^35^VAL	K26-Azo-K27	89
	P9 (e)	^25^R-Azo-KLQ^30^DVHNF^35^VAL	K26 → Azo	86
	P10 (i)	^25^RKKLQ^30^DVHN-Azo-F^35^VAL	N33-Azo-F34	-
C-terminal	P11 (i)	^25^RKKLQ^30^DVHNF^35^V-Azo-AL	V35-Azo-A36	-
	P12 (e)	^25^RKKLQ^30^DVHNF-Azo-AL	V35 → Azo	63

a The corresponding peptides were prepared by on-resin synthesis (Merrifield-synthesis) using Fmoc-based building blocks. The synthesis of the AMPB photoswitch follows previously published methods.^22^ The half-life time *t*_1/2_ (hours) of the cis-form of the peptides was investigated via UV/vis spectroscopy (see the experimental section for further experimental details).

**Figure 4:**
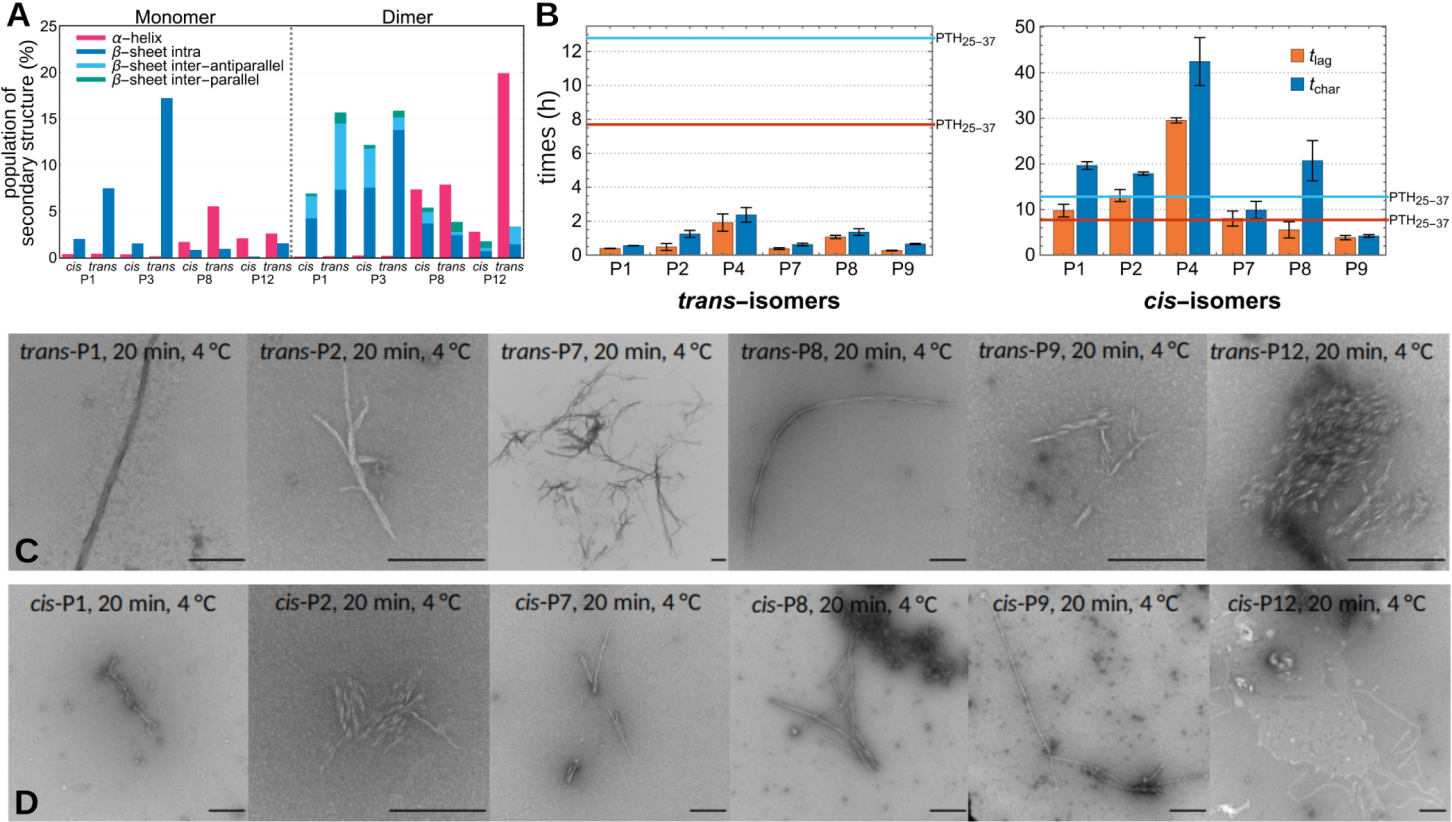
(A) Simulated average secondary structure population, divided into α-helical, intrapeptide β-sheets, and interpeptide parallel and antiparallel β-sheets as indicated by the color code. Results are shown for simulations of monomers and dimers of the *trans*- and *cis*-forms of P1, P3, P8, and P12. (B) Lag time *t*_lag_ (orange) and characteristic time *t*_char_ (blue) of the *trans*-forms (left) and *cis*-forms (right) of the peptides P1, P2, P4, P7, P8, and P9. The corresponding values of PTH_25–37_ are indicated as orange (*t*_lag_) and blue line (*t*_char_). (C) TEM images of the *trans*-isomers of P1, P2, P7, P8, P9, and P12 after 20 min at 4 °C. Scale bar = 250 nm. (D) TEM images if the *cis*-isomers of P1, P2, P7, P8, P9, and P12 after 20 min at 4 °C. Scale bar = 250 nm. TEM data for the photoswitching of P4 are presented in Figure S23.

Our design strategy is based on the assumption that moving the AMPB group to the N-terminal side will enhance fibrillization by introducing the hydrophobic AMPB into the polar and charged N-terminal region, whereas moving it to the C-terminal side should impair amyloid formation by disrupting the amyloid-prone peptide region. In addition, we expect that the difference between the *cis-* and *trans*-forms of AMPB will become less important as it is shifted toward the termini, since hairpinning should no longer be possible in the *cis*-form. To refine these predictions, we performed MD simulations of the P1, P3, P8, and P12 variants, considering both the *cis*- and *trans*-configurations of each peptide as monomer and dimer (Figure 4A; P1 was also simulated as hexamer, see Figure S9). The simulations of P1 revealed a similar tendency for β-sheet formation as seen for P4 (Figure 2D), which makes sense given that AMPB is at the same position in both peptides, but instead of replacing V31 as in P4 it is added between D30 and V31, thereby extending the hydrophobic stretch on the C-terminal side of the peptide (Figure S10), which might lead to a faster aggregation kinetics in the experiments. In P3, the AMPB group is shifted by one position toward the N-terminus compared to P4, replacing D30. This increases the hydrophobicity of the peptide in support of aggregation, while the removal of the negative charge increases the overall positive peptide charge, which could discourage aggregation. The simulations revealed that the increase in hydrophobicity prevails, as random dimerization with mainly intrapeptide instead of interpeptide β-sheets dominated in the simulations of *trans*-P3. P8 was simulated as a representative peptide in which AMPB is significantly shifted toward the N-terminus and inserted between K26 and K27. The electrostatic potential surface (Figure S10) shows that the insertion of the hydrophobic AMPB into the positively charged N-terminal region indeed significantly increases its hydrophobicity, promoting fibrillization. However, the secondary structure preferences are somewhat shifted away from β-sheet toward the formation of α-helices in both *cis*- and *trans*-configurations and both as monomers and dimers, which was not seen for the P3 and P4 variants and may counteract amyloid fibrillization. Finally, we examined the P12 mutant in which the AMPB photoswitch is introduced into the aggregation-prone C-terminal region in the form of an exchange of V35. As predicted, the simulations confirm a drastic decrease in β-sheet formation compared to PTH_25–37_ and the other simulated peptide designs. As with P8, where AMPB is placed at the N-terminal side, its C-terminal position in P12 also leads to the formation of α-helices, which could also counteract amyloid aggregation.

Next, we investigated the influence of the AMPB site experimentally. The designed peptides displayed varying solubility in a buffered aqueous solution (50 mM Na_2_HPO_4_, pH 7.4), ranging from 370 μM for P4 to 7 μM for P10 (Table S2), thus limiting the assay-copnditions to those wherein sufficient critical concentrations could be reached. The critical concentration (*c*_cr_) of monomers in a fibril-forming system is the minimum concentration required to form fibrils in the dynamic equilibrium between the fibrils and the monomeric peptide form, which can be converted to a standard free energy of the fibril elongation reaction (Δ*G*^0^). Since the *c*_cr_ of PTH_25–37_ is 42 μM at the chosen conditions, we decided to exclude the peptides with lower solubility, also motivated by the observation that no fibrillization was observed in P3 and P5, due to their low solubility. The peptides with small *c*_cr_ are the ones in which the hydrophobicity in the otherwise rather polar and positively charged peptide region was increased (P3, P5, P6), or in which the already hydrophobic C-terminal region was made more hydrophobic by placing the AMPB there (P10, P11). The reduced solubility of P3 is consistent with our MD simulation prediction of random aggregation. For the remaining peptides (P1, P2, P7, P8, P9, and P12, in addition to P4), we determined the photophysical characteristics of the isomerization reaction, such as the half-life time *t*_1/2_ of the thermodynamically less favorable *cis*-isomer, which is influenced by the position of the azobenzene switch (Table 1). To ensure probing of the fibrillization kinetics, it is important that the stability of the *cis*-isomer is significantly larger than the lag time of the fibrillization process. As both process are competing we strived to minimize the rate of *cis* → *trans* isomerization to primarily investigate the behavior of the *cis*-isomer in the relevant time range. These half-life times range from 63 to 97 h, with the longest time observed for P4, where the central V31 was replaced by AMPB, and the shortest time, when the azo switch was shifted toward the C-terminus, replacing V35. The increased half-life time can be explained by the energy barrier for the *cis* → *trans* isomerization in the ground state due to interaction between the residues at either side of the AMPB group (Figure 2G), which is also reflected in the *cis*:*trans* ratio. After synthesis, this ratio is between 3:97 and 6:94 for the freshly prepared peptides, subsequently increasing during the photo-induced *trans* → *cis* isomerization to the photostationary state (PSS), reaching values of 90:10 to 82:18. Isomerization back under dark conditions reduces this ratio to 19:81 and 24:76 in the PSS of *cis* → *trans* isomerization (Table S3). To exclude a photobleaching effect of the AMPB photoswitch, we repetitively conducted alternating *trans* → *cis* and *cis* → *trans* isomerizations over 5 cycles (Figure S11). When monitoring the absorption at two wavelengths (absorption maxima of *trans*- and *cis*-isomer, respectively) we did not observe a decline in the overall absorption, except for the first cycle, which can be explained by the fact that the initial peptides directly after synthesis possess an increased *trans*-content compared to the subsequent photostationary state. To assess the toxicity of the peptides with the incorporated photoswitch, we conducted cytotoxicity assays with the N-terminally modified P8, the centrally modified P4, and the C-terminally modified P12 (Figure S12), and we did not observe any toxicity toward 3T3 and NHDF cells.

We then examined the fibrillization process of the peptides using the ThT fluorescence assay after an established protocol for PTH_1–84_^20^ (Figures S13–S20) and followed the formation of fibrils with TEM images after 20 min at 4 °C and after 1, 3, 24, and 96 h at 37 °C (Figures S21–27, original data for the photoswitching (TEM) for P4 are presented in Figure S23). The ThT fluorescence curves were fitted using eq. 1 and yielded the lag time *t*_lag_, which corresponds to the onset of the fibril growth phase, and the characteristic time *t*_char_, the point at which the fluorescence intensity reaches 50% of its maximum (Figures 4B, S13–20). Compared to PTH_25–37_, the fluorescence intensity was significantly lower, resulting in a poorer signal-to-noise ratio. We attribute this to fluorescence quenching by the azobenzene moiety, which is an already known property of this molecular building block.^10 g,22^ Nevertheless, a sigmoidal fibrillization curve is clearly visible for the three replicate measurements of each peptide. Peptides P1, P2, P4, P7, P8 and P9 show typical fibrillization behavior with lag phase, growth phase, and stationary phase (Table S4). This observation is consistent with our prediction that the placement of AMPB in the middle or at the N-terminus of PTH_25–37_ should not prevent the amyloid aggregation ability of the peptide. All *trans*-peptides exhibited, irrespective of the positioning of the photoswitch, a faster fibrillization process than PTH_25–37_ with significantly shortened lag phases (*t*_lag_ of 8 h for PTH_25–37_ vs 0–2 h for the peptide designs) and reduced *t*_char_ (Figure 4B). We assume that the increased hydrophobicity and the organizing effect of the AMPB group are the driving forces for the increased tendency of amyloid aggregation. TEM images of the fibrils were obtained for all peptides after only 20 min at 4 °C (Figure 4C). The *cis*-forms of peptides P1, P2, and P4, in which AMPB was inserted at the central position next to the aggregation hot-spot sequence H32-L37, showed slower fibrillization compared to PTH_25–37_, whereby for *cis*-P1 and *cis*-P2 the fibrillization occurred in a similar time range, while the fibrillization of *cis*-P4 was strongly delayed. In agreement with our predictions, the incorporation of the photoswitch in its *cis*-form at the N-terminal part had almost no influence on the fibrillization kinetics: *cis*-P7 and cis-P8 aggregated at a similar rate as PTH_25–37_, while the fibrillization of *cis*-P9 was even faster (due to the increased hydrophobicity of the peptide). The TEM images (Figures 4D, S21–27) confirm these observations. For the *cis*-isomers of P1 and P2, we mainly observed amorphous aggregates in the early images (after 20 min); the first objects, which could be clearly assigned to fibrillar aggregates, as with the *trans*-forms, were only detected after 24 h. For P4 (Figure S20) the difference was even more significant: in the *cis*-form, the first fibrillar aggregates were visible in the image only after 60 h. For the N-terminally modified peptides *cis*-P7, *cis*-P8, and *cis*-P9, however, fibrils were already visible after 20 min.

For the fibrillization behavior of the C-terminally modified P12, where V35 was replaced by AMPB, we found that it is entirely different. Unlike other peptides, ThT fluorescence curves for both *trans*- and *cis*-P12 show a linear increase after a lag phase of approximately 20 h, with no saturation observed even after 140 h, indicating reduced fibrillization. This also holds true for fibril morphologies, as both P12 isomers exhibit similar shorter fibrils in the TEM images, with *trans*-P12 forming fibril-like structures after 1 h and *cis*-P12 after 3 h.

In summary, our study revealed that the impact of the photoswitch varies depending on its placement: when positioned within the fibril-forming segment, such as in P12, fibril formation is significantly hindered, whereas outside this crucial region, the *trans*-isomers demonstrate faster fibril formation compared to PTH_25–37_. Notably, *cis*-isomers display a strongly hindered fibril formation or the formation of amorphous aggregates, with the inhibition decreasing as the distance from the fibril-forming C-terminal region increases.

### Photocontrolled Multiple Switching of Fibril Formation

**Figure 5:**
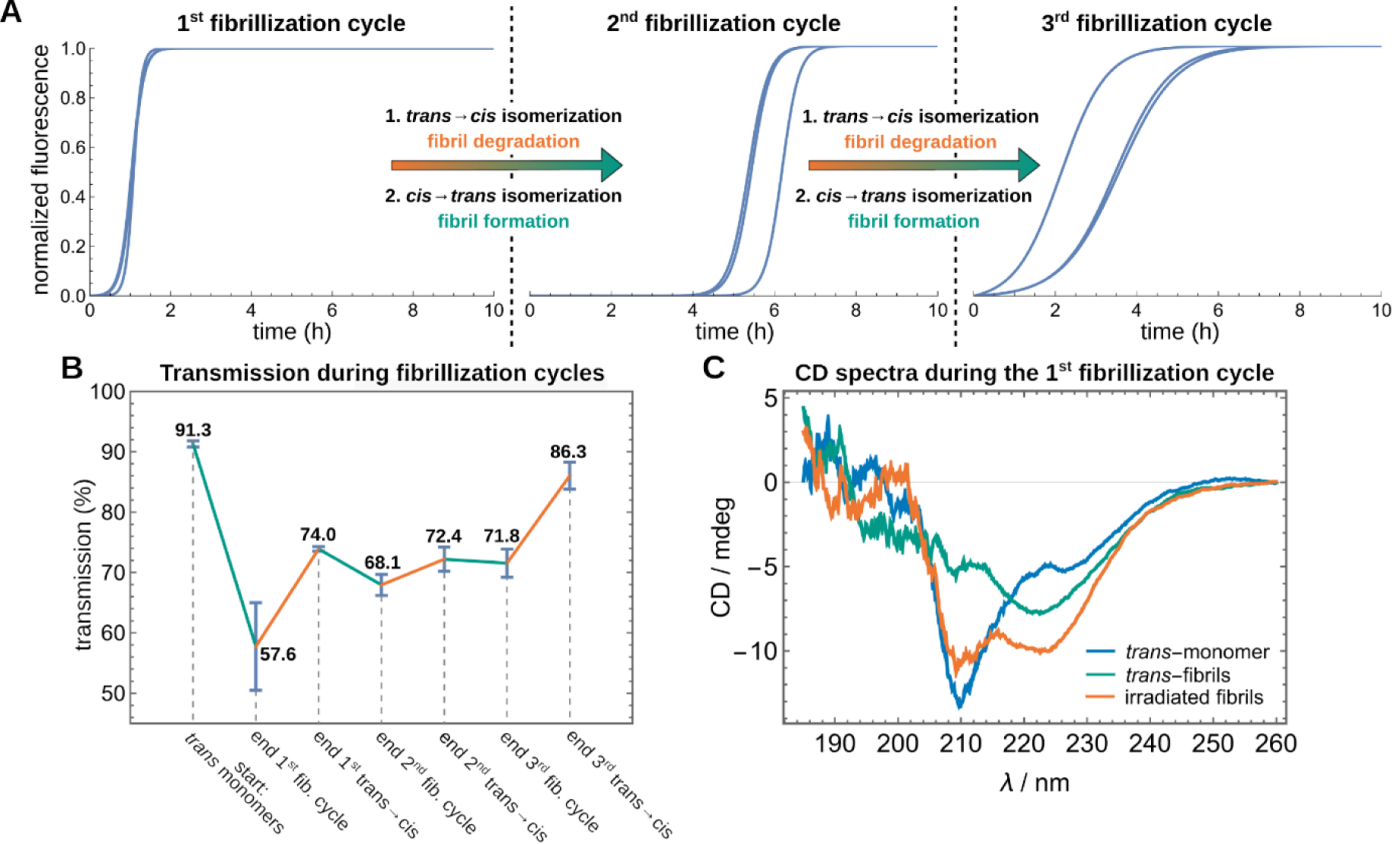
Multiple fibrillization and degradation cycles of *trans*-P4 fibrils through photoisomerization. (A) Fitted ThT signal monitored by the fluorescence assay of three fibrillization cycles of *trans*-P4 comprising degradation of fibrils by photoinduced *trans* → *cis* isomerization and subsequent photoinduced *cis → trans* isomerization leading to renewed fibrillization. (B) Change in the transmission of a P4 sample over several switching cycles (cyan for fibrillization periods and orange during *trans* → *cis* isomerization). (C) CD spectra of monomeric *trans*-P4 (blue), *trans*-P4 fibrils (green), and the fibrils after *trans* → *cis* isomerization *via* irradiation with 340 nm for 5 h (orange).

By computationally analyzing the primary structure, modeling the peptide structures in their monomeric and aggregated forms, and studying the fibrillation process of PTH_25–37_ and the designed peptides, we aimed to determine the most suitable position for the incorporation of the photoswitch into the peptide sequence to control fibril formation. Based on the results obtained, we applied several criteria to select the most promising candidate from the designed peptides (Table 1) to test whether amyloid formation can be reversibly switched on and off by light. After excluding peptides with insufficient solubility (P10 and P11) as well as the poorly aggregating P12, the focus was placed on the peptides P1, P2, and P4, which show accelerated fibrillization due to the central placement of the photoswitch in the *trans*-conformation, while the *cis*-form slows down fibrillization. Of these peptides, P4 exhibited both the best solubility and the largest difference in fibrillization for *trans*- and *cis*-P4. Therefore, we selected this peptide P4 to further test whether we could achieve reversible fibril formation (Figures 5, S19, S28). It should be noted that during the following sequence of experiments, the cuvettes were not changed; moreover, also no new peptides were added to the solution. Starting from the *trans*-isomer of P4, fibrils were formed in the first cycle at 37 °C in phosphate buffered aqueous solution and the aggregation kinetics followed via ThT fluorescence (Figure 5A, left). After the fibrillization passed over to the stationary phase, the solution was shaken another 20 h to affirm that the equilibrium has been established. Subsequently, the suspension was irradiated at a wavelength of 340 nm for 5 h under stirring, after which UV/vis spectroscopy was used to verify that the *cis*-photostationary state of P4 was reached. Now, the solution was irradiated for 20 min with light of 405 nm wavelength to switch the *cis*-PSS to the *trans*-PSS to produce monomeric *trans*-peptides, which underwent another fibrillization cycle (Figure 5A, middle). The newly formed fibrils were again exposed to the photoisomerization treatment involving *trans* → *cis* isomerization to degradate the fibrils. The ThT-monitored fibrillization assay was repeated with a portion of the solution, and fibril formation was observed for a third time (Figure 5A, right). For the second and the third fibrillization cycle, a decreased starting concentration of the *trans*-isomer was observed (UV/vis spectra, Figure S29). As we could already exclude photobleaching, we suppose that aggregates adhere to the wall of the 96-well plates and are, therefore, not available for further fibrillization cycles. The fibrils formed during the fibrillization process can precipitate and thus disturb the measurement via scattering effects, causing noise in the signal after the first stationary phase. Because the *trans* → *cis* isomerization of the AMPB-switch is not reaching 100% due to the photostationary state, there certainly are fibrillar aggregates present in the solution, which, however, may interfere in the subsequent fibrillization processes and thus change the following fibrillation kinetics. Nonetheless, for each new fibrillization process monitored here, a lag phase, growth phase, and stationary phase were observed.

While monomers remain soluble, fibrils tend to precipitate, causing the solution to become turbid during the fibrillization assay and leading to a decrease in transmission. Observing the transmission across three switching cycles, it was evident that transmission decreases during the fibrillization process (Figure 5B, cyan lines). Degradation of the fibrils via *trans* → *cis* isomerization, however, led to an increase in transmission, as released monomers dissolve back into the solution (Figure 5 B, orange lines). The incomplete recovery of the transmission during degradation results from the aforementioned observation that the *trans* → *cis* isomerization only leads to a maximum *trans*:*cis* ratio of 18:82. Therefore, the degradation process of the fibrils is an only partial one, presumably as a full isomerization cannot be reached. The changes in secondary structure during fibrillization were probed by CD spectroscopy (Figures 5C, S28). In its monomeric form, *trans*-P4 displays a shoulder around 220–230 nm and a minimum around 210 nm. In the CD spectrum of the *trans*-P4 fibrils, a minimum is observable between 220 and 230 nm, while the minimum around 210 nm is nearly completely absent. Degradation of the fibrils by the *trans* → *cis* isomerization leads to a CD spectrum where the minimum around 210 nm reappeared and the other minimum between 220 and 230 nm still exists. The minimum at 210 nm arises from the monomeric peptides, while the minimum between 220 and 230 nm is indicative for the fibril form. The observed CD spectrum is thus a superposition of both forms, the monomeric and the fibrillated form. From the CD spectra, we can, therefore, conclude that fibrillization from the *trans*-form and incomplete fibril degradation during *trans → cis* isomerization can be observed.

Fibrillization of proteins are complex, often irreversible processes, which are characterized by a strong thermodynamic negative free energy, as e.g., in Aβ fibrillization, and thus conventionally termed as “irreversible.”^58^ This is often connected with a high kinetic barrier, usually preceding the fibrillization process, wherein nucleation is central to initiate that nucleation process. The PTH peptides studied here are “more reversibly” fibrillating peptides, wherein the thermodynamic stabilization of the amyloid state is less and, therefore, also the kinetic barriers.^15,59^ Nonetheless, until now, it has not been possible to control the reversibility of this process, as accomplished here via the introduction of a photoswitch. However, in contrast to other reversibly (photo)-switchable systems, such as reported for small molecule assemblies,^60^ adhesives,^61^ or photoswitchable enzymes,^62^ a full reversion of the aggregation of the current system is not reached, as expected, as the fibrillization and defibrillization processes depend on many factors, such as the kinetic barriers in either direction, the kind and number of nuclei present during the different switching cycles, or also the precipitation of fibrils.

## Conclusion

In this study, we have demonstrated precise control over reversible peptide fibrillization by strategically positioning a photoswitch within the central region of a fibril-forming peptide. Utilizing the artificial light switch 3-{[(4-aminomethyl)phenyl]diazenyl}benzoic acid (AMPB) embedded in a peptide containing residues 25–37 of the parathyroid hormone (PTH), we investigated the impact of position on peptide aggregation. Through a comprehensive approach involving computational modeling, peptide synthesis, aggregation assays, and structural analyses, we elucidated key features governing the fibrillization of both unmodified and modified PTH_25–37_ peptides. Notably, the *trans*-peptides with the modification positioned adjacent to the fibril-forming region in the center of the peptide (P1, P2, and P4) displayed enhanced fibrillization compared to unmodified PTH_25–37_, while the aggregation is slowed down for their *cis*-isomers. Peptides featuring the photoswitch in the nonamyloidogenic N-terminal region behaved similarly to unmodified PTH_25–37_ (P7, P8, and P9), whereas peptide P12, where the azobenzene unit replaced V35 in the amyloid-prone region, showed decreased fibrillization, largely unaffected by the photoswitch’s isomer state. Our bioinformatics and simulation analysis uncovered that modification with AMPB typically boosts the peptides’ hydrophobicity, thereby augmenting their tendency to aggregate. Furthermore, AMPB facilitates self-interaction among peptides through π–π interactions, further enhancing their aggregation propensity. Structural investigations of P4 employing WAXS and ssNMR suggest that β-strands in amyloid fibrils of P4 are—in contrast to fibrils of unmodified PTH_25–37_— aligned antiparallel. Simulations suggest that AMPB might not fully adhere to the amyloid fold, owing to the distinct demands for interpeptide distances in π–π interactions and fibril formation. This elucidates the diminished fibrillization observed when the photoswitch is positioned within the amyloid-forming segment of the peptide, as seen in P12. Importantly, our results showcase the potential of strategically placing the azo photoswitch, particularly exemplified by peptide P4, to control reversible amyloid aggregation. The phototriggered degradation of fibrils formed in the *trans*-state of AMPB enables repeated fibril formation, which in fact may allow to reversibly modulate the fibrillization of the PTH hormone, with the monomeric peptides released upon fibril degradation serving as the active form and the fibrillar structures acting as an inert peptide reservoir. Given the peptides’ nontoxic nature (Figure S12), this light-triggered approach, therefore, presents a promising method for controlled drug delivery and release of such reversibly fibrillating peptides. We regard our system here as a switchable fibrillization system, where a phototrigger from the outside is able to induce fibrillization, as e.g., needed in many modern neuro-cellbiology systems.^63^

## Experimental Section

### Materials

All technical solvents were distilled prior use; air- and moisture-sensitive reactions were carried out in flame-dried glassware under atmospheric pressure of nitrogen. 2-(6-Chloro-1-H-benzotriazole-1-yl)-1,1,3,3-tetramethylaminium hexafluorophosphate (HCTU), *N*-methyl-morpholine (NMM), *N*,*N*-dicyclohexylcarbodiimide (DIC), *N*-Hydroxybenzotriazole (HOBT), trifluoroacetic acid, 4-amino-benzylamine, and oxone were purchased from Sigma-Aldrich. 9-Fluorenylmethyl-*N*-succinimidylcarbonat (Fmoc-OSu) was received from Fluorochem. 3-Aminobenzoic acid was purchased from Merck and was used without further purification.

### UV/Vis, CD, and Transmission Spectroscopy Measurements

UV/vis-absorbance spectroscopy was measured on a JASCO V-660 absorbance spectrometer in a 1 cm quartz glass cuvette. For PTH_25–37_, the absorbance was measured at 205 nm with a molar extinction coefficient of 49.310 cm^–1^ M^–1^; *trans*-azobenzene containing peptides were measured at 327 nm with a molar extinction coefficient of 13.000 cm^–1^ M^–1^. CD spectroscopy was measured with a JASCO J-1500 CD Spectrometer in either a 1 mm. As buffered solution, a 50 mM aqueous Na_2_HPO_4_ buffer solution was used with a pH value adjusted to 7.4. Transmission was measured with a Litesizer DLS 500 from Anton Paar using a 3 mm × 3 mm quartz glass cuvette. The irradiation wavelength was 658 nm. The equilibration time was 1 min, and the measurement time was 10 s. The temperature was maintained at 25 °C.

### Peptide Synthesis

The 3,4′-AMPB photoswitch was synthesized in two steps according to our published procedure.^22^ Solid-phase peptide synthesis was utilized on an automated peptide synthesizer MultiPep RS (Intavis AG, Koeln, Germany) using standard Fmoc-chemistry and preloaded resins. Standard coupling of all protected natural amino acids was performed as single couplings in dimethylformamid (DMF) using 5 equiv of amino acids, HCTU as coupling reagents, and 10 equiv of NMM as base for 1 h at room temperature. Special building groups, such as Fmoc-3,4′-AMPB, were coupled with 3 equiv using DIC and HOBT in DMF/ *N*-methyl-2-pyrrolidone (NMP) at room temperature and with gentle shaking in the dark overnight. The N-terminal Fmoc protecting group was removed by washing the resin with 20% piperidine for 20 min. The final side chain deprotection and cleavage from the resin employed a mixture of trifluoroacetic acid and water (90:10 Vol%) with gentle agitation for 2 h at room temperature. The crude peptides were purified to >95% purity using preparative RP-HPLC (Gilson, Limburg, Germany). For both analytical and preparative use, the mobile phase was a mixture of water (eluent A) and acetonitrile (eluent B), respectively, each containing 0.1% trifluoroacetic acid. Samples were eluted with a linear gradient from 5% B to 95% B in 15 min for analytical runs and in 90 min for preparative runs on a semipreparative PLRP-S column (Agilent Technologies, 300 × 25 mm, 8 um). Finally, all peptides were characterized by analytical HPLC Dionex Ultimate 3000 (Thermo Scientific, Germany) using a PLRP-S column (Agilent Technologies, 150 × 4.6 mm, 3 um) and MALDI-MS (Bruker Microflex LT, Bremen, Germany), which gave the expected [M + H]^+^ mass peaks. The full molecular characterizations are shown for all peptides in Figures S30–S47.

### Aggregation Kinetics

The fibrillization process was investigated using a thioflavin T (ThT) monitored fluorescence assay following the established process for PTH_1–84_.^20^ Therefore, the fluorescence intensity of ThT was measured. Lyophilized peptides were dissolved in buffered solution and kept on ice for the next steps. The sample solutions were centrifuged at 13 000 rpm for 10 s, and the concentration of the respective peptide was determined using UV/vis-absorbance spectroscopy. If required, the *cis*-isomer of the azobenzene containing peptides was produced as described below. The sample solutions were centrifuged at 10 000 rpm for 1 h at 4 °C, and the supernatant was transferred to another tube. The solutions were diluted with buffer, and ThT was added as a 1 mM stock solution to obtain a final concentration of 100 μM for the peptides, with a solubility above 100 μM, and 50 μM for ThT. For the other peptides, the solutions were solely diluted with the ThT stock solution, to achieve the highest possible concentration. For each sample, a total volume of 480 μL was prepared, and three aliquots with 150 μL were transferred to a medium binding 96-well plate (GREINER Bio-One 675 076). The plate was sealed with a microplate cover, and the fluorescence intensity was monitored at 37 °C using a BMG FLUOStar Omega multimode plate reader using fluorescence excitation and emission wavelengths at 460 and 485 nm, respectively. One measurement cycle lasted 5 min, consisting of double-orbital shaking for 150 s and incubating for 150 s. To describe the fibrillization process, the source data were fitted. As the description of fibrillar growth in terms of the molecular rate, kinetic can be considered as two main fibrillation processes.^37^ Their contributions to the increase in fluorescence Δ*F (t)* can be analyzed by a function, which was derived by Dear et al.^38^ and has been used to characterize the fibrillation kinetics of full-length PTH.^20^
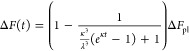
1

Δ*F*_pl_ corresponds to the plateau value of the fluorescence in the stationary phase, while λ and κ are the rate constants of the primary and secondary nucleation processes.

### Determination of the Solubility Parameters

To determine the maximal solubility of a peptide, small amounts (0.5 mg) of the respective peptide were added to 1 mL of buffered solution and shacked for 10 s until an insoluble precipitate was visible. The suspensions were centrifuged at 10 000 rpm for 1 h at 4 °C, and the supernatant was transferred to another tube. The remaining monomer concentration was determined using UV/vis-absorbance spectroscopy. To determine the critical concentration *c*_cr_, two samples were prepared for each peptide according to the procedure for the ThT monitored fibrillization assay, one with ThT as a reference sample and the other one without ThT. Twenty hours after the reference sample reached the stationary phase, the fibril containing solutions from the sample without ThT were transferred to a tube and centrifuged at 10 000 rpm for 1 h at room temperature. The supernatant was transferred to another tube, and the concentration of the remaining monomers was determined using UV/vis-absorbance spectroscopy at 278 nm with a molar extinction coefficient of 3750 cm^–1^ M^–1^ for the azobenzene containing peptides and at 205 nm with a molar extinction coefficient of 49 310 cm^–1^ M^–1^ for PTH_25–37_.

### Photosiomerization

The photoisomerization of the *trans*-azobenzene moiety in the peptides was performed by irradiating the dissolved peptides in a 1 cm quartz cuvette for 30 min with light of 340 nm wavelength using a 69.2 mW LED (Thorlabs, M340L5) equipped with a controller (Thorlabs, LEDD1B). The photoisomerization of the *cis*-azobenzene moiety in the peptides was performed by irradiating the dissolved peptides in a 1 cm quartz cuvette for 30 min with light of 405 nm wavelength using a 1.4 W LED (Thorlabs, M405L4) equipped with a controller (Thorlabs, LEDD1B).

### Photobleaching

To test whether photobleaching occurs during the photoisomerization, a solution of 50 μM P4 was irradiated alternatingly for 30 min with light of 340 nm wavelength (*trans* → *cis* isomerization) and light of 405 nm wavelength (*cis* → *trans* isomerization). The absorption was measured after each isomerization step at two wavelengths: 295 nm (absorption maximum of the *cis*-isomer) and 327 nm (absorption maximum of the *trans*-isomer).

### Transmission Electron Microscopy

TEM images were taken with an electron microscope (EM 900; Zeiss) at 80 kV acceleration voltage. For preparation, 5 μL of the peptide solution were added on Formvar/Cu grids (mesh 200). After 3 min of incubation, the grids were gently rinsed two times with water and then negatively stained using uranyl acetate (1%, w/v) for one minute.

### Fibril Degradation

To investigate the fibril degradation through photoisomerization, 3 mL of a 100 μM *trans*-P4 in buffer was prepared according to the procedure for the ThT monitored fibrillization assay, except that ThT was excluded, and aliquots of 150 μL were transferred to a medium binding 96-well plate. As a reference sample, 480 μL of a 100 μM *trans*-P4 in buffer was prepared with ThT, and both approaches were incubated as described for the ThT monitored fibrillization assay. Twenty hours after the reference sample reached the stationary, the fibril containing solutions of the sample without ThT were collected in one tube. UV/vis-absorbance and CD were measured, and the fibril solution was transferred in a 1 cm quartz glass cuvette and treated according to the procedure for the *trans* → *cis* isomerization, except that the irradiation time was prolonged to 5 h. Again UV/vis-absorbance and CD were measured. Subsequently, the *cis*-isomer was photoisomerized back to the *trans*-form. 456 μL of the solution was mixed with 24 μL of a 1 mM ThT stock solution and transferred as 150 μM aliquots to a medium binding 96-well plate for a ThT monitored fibrillization assay. The remaining solution was transferred as well in 150 μL aliquots to the 96-well plate and incubated under the same conditions. This procedure was repeated for every degradation cycle.

### Solid-State NMR Spectroscopy

For the solid-state NMR spectroscopy, 6 mL of a 100 μM solution of the respective peptides (PTH_25–37_ and P4 with uniformly ^15^N/^13^C labeled residues L28 and F34) was prepared according to the procedure for the ThT monitored fibrillization assay and transferred in 150 μL aliquots to a medium binding 96-well plate. Twenty hours after the stationary phase was reached, the fibril containing solutions were collected in a tube and centrifuged at 60 000 rpm for 15 min at 10 °C. The supernatant was discarded, the remaining fibril pellet was frozen with liquid nitrogen, and the residual water was removed using lyophilization. The lyophilized peptides were packed into Bruker MAS rotors with outer diameters of 3.2 mm (PTH_25–37_) and 1.9 mm (P4), respectively. Solid-state NMR experiments were conducted on an 18.8 T (800 MHz ^1^H frequency) Bruker Avance III spectrometer equipped with a triple resonance HCN 3.2 mm MAS Efree probe and a 1.9 mm triple resonance probe. For determination of intermolecular long-range contacts between L28 and F34 resonances, proton-driven spin diffusion (PDSD) experiments with an MAS frequency of 20 kHz close to the rotational resonance condition between aromatic ^13^C resonances of F34 and aliphatic C_γ_ /C_δ_ resonances of L28 were recorded with mixing times ranging from 50 ms to 1 s. For PTH_25–37_, additional PDSD and DQSQ spectra were recorded on a 14.1 T (600 MHz ^1^H frequency) Bruker Avance wide bore spectrometer equipped with a with a 3.2 mm MAS triple resonance ^1^H, ^13^C, ^15^N probe. Typical radiofrequency field strengths were 91–100 kHz for ^1^H, and 55.6 kHz for ^13^C Spinal64 ^1^H decoupling^39^ (rf field of 85 kHz) was applied during ^13^C evolution and acquisition. The VT gas temperature was set to 263 K (thermocouple reported temperature); the sample temperature was estimated to be around 10–20 K higher due to frictional heating under MAS.

### WAXS Measurements

The WAXS measurements were performed in transmission mode with a SAXSLAB laboratory setup (Retro-F) equipped with an AXO microfocus X-ray source. As a monochromator, the AXO multilayer X-ray optic (AXO Dresden GmBH, Dresden, Germany) was used for Cu–Kα (λ = 0.154 nm). The two-dimensional scattering patterns were recorded with a two-dimensional detector (PILATUS3 R 300 K, DECTRIS, Baden, Switzerland). The preformed fibrils were prepared as described for the measurement of the aggregation kinetics in a total volume of 1 mL. Twenty hours after the fibrillization reached the stationary phase, the fibril suspension was ultracentrifugated at 60.000 *g* for 15 min, and the obtained pellet was transferred into a glass capillary and dried overnight. The scattering measurements were performed at room temperature in vacuum and corrected for background.

### Cytotoxicity Tests

Cell viability was determined for *trans*-P4, *trans*-P8, and *trans*-P12 on NHDF as well as 3T3 fibroblasts with a resazurin reduction assay. Briefly, cells were seeded at desired cell densities in corresponding culture media supplemented with 10% FCS and penicillin/streptomycin in 96-well plates on day 0 and incubated overnight under standard cell culture conditions. On day 1, serial dilutions of the peptides in cell culture media were prepared. Then, medium (background and negative control–100% viability), TritonX in medium f.c. 0.025% (postive control–0% viability), and the peptide dilutions were added with one treatment per column (*n* = 8). Incubation for 24 h or 96 h under standard cell culture conditions followed. On day of measurement, resazurin stock solution was added to a final concentration of 44 μM into each well. After 2 h incubation, the resorufin fluorescence was measured with a Cytation 5 plate reader system. Means and standard deviation of each column were calculated. Experiments were repeated independently 3 times, and average values were evaluated (see Figure S12).

### MD Simulations

For all MD simulations of the PTH_25–37_ peptide, as well as the mutants P1, P3, P4, P8, and P12, we used the GROMACS simulation package.^40^ Since the PTH_25–37_ sequence classifies as an intrinsically disordered protein (IDP), we have used the CHARMM36m force field^41^ to model protein interaction, which has previously been shown to be a suitable choice for IDPs.^42^ For the azobenzene photoswitch (denoted AZO in our force field implementation), we parametrized the interactions following the cgenff standard protocol^43^ and refined the parametrization using data from QM/MM simulations,^44^ by fitting our parameters to reproduce the bond, angle, and dihedral angle distributions of the *cis-* and *trans*-state as obtained from QM/MM. The resulting force field parameters are available at https://github.com/strodel-group/Charmm36m_Azobenzene-FF. During the MD simulations of the modified PTH_25–37_ peptides, the ∠CNNC dihedral angle was restricted to either the *cis-* or *trans*-states; thus, transitions between the two states of the AMPB photoswitch were not modeled. The MD simulations of all systems were prepared following the same protocol: first, the peptide(s) were placed in the simulation box, where in case of the dimer and hexamer simulations, the box size was always chosen to achieve a peptide concentration of 10 mM. Then, the box was filled with TIP3P water molecules,^45^ as well as Na^+^ and Cl^–^ ions to neutralize the system and achieve a physiological salt concentration of 150 mM. After equilibration of the systems, a production run of 10 μs per system (1 μs for the fibril models) was carried out under *NpT* conditions at constant number of particles *N*, pressure *p* = 1, bar and temperature *T* = 300 K. The pressure and temperature were regulated using the Parrinello–Rahman pressure coupling scheme^46^ and Nosé–Hoover thermostat,^47^ respectively. To exclude edge effects, periodic boundary conditions in all directions were applied, and the particle-mesh Ewald method^48^ was used to calculate electrostatic interactions. For the calculation of van der Waals and Coulomb interaction in real space, a cutoff of 12 Å was applied. An overview over all simulations performed is given in Table S1, yielding 285 μs of total simulation time across all systems. All MD simulations were run on the high-performance cluster JURECA-DC.^49^ Analysis of the MD data was performed with python using the MDAnalysis^50^ package for reading of the MD trajectory and the calculation of distances between groups of atoms, while the MDTraj^51^ package was used for secondary structure analysis. For visualization of the MD structures, the PyMOL^52^ software was used. Additionally, PyMOL in combination with the APBS^53^ plugin was used for the calculation and visualization of electrostatic potential surfaces.

### QM Calculations

The photoswitching mechanism of AMPB integrated into PTH_25–37_ was monitored in the ground and excited states along the ∠CNNC dihedral angle for the *cis* ↔ *trans*-transition of the azobenzene photoswitch. As a starting point for the QM calculations, we constructed pathways from *cis* to *trans* and *vice versa*, using MD simulations. To consider the structural ensemble along the pathway, we simulated 40 *cis* ↔ *trans* switching trajectories. We started the switching from equilibrated *cis*- and *trans*-P4 structures obtained from equidistant time steps of 250 ns from the 10 μs MD simulations. The switching in the MD simulations was achieved by imposing a restraining potential on the ∠CNNC dihedral angle and changing it every 2 ns in increments of 10° between 0° and 180°. All other MD simulation parameters were the same as described above. The resulting trajectories were used as input for the subsequent QM calculations. The QM calculations were conducted using the ONIOM-based QM/QM2 method,^30^ with NEVPT2(2,2)/def2-TZVP^31,54^ for QM and xTB2^32^ for QM2, as implemented in the ORCA program package.^55^ The QM region was focused on the azobenzene core, including the peptide in π-conjugation with it. The solvent was implicitly modeled using the ALPB method.^56^ Due to convergence issues, analysis was limited to 34 of the original 40 trajectories. For these paths, the energies of electronic states S_0_, S_1_, and T_1_ were interpolated to obtain potential energy curves using Gaussian process regression from the scikit-learn package.^57^ Some of the paths that have an energy barrier at dihedral angles around 170° were analyzed at the structural level. To this end, the inter-residue distances based on the centers of mass of the residues were calculated for all structures exhibiting a dihedral angle near 170° and then analyzed through dimensionality reduction via principal component analysis (PCA). The first three principal components were transferred back into the original distance matrix format and illustrated with three representative distance matrices from the corresponding cluster of structures. Representative structures for the distance matrices were also extracted and visualized.

## Supporting Information Available

**Supplementary figures** S1, QM potential energy curves for 34 *cis* ↔ *trans* isomerization paths; S2, fluorescence lifetime measurements; S3, PCA analysis of selected P4 structures; S4, principal components projected onto inter-residue distance space; S5, selected P4 snapshots; S6, WAXS data of P4 and PTH_25–37;_ S7 ssNMR data for PTH_25–37_ fibrils; S8, ssNMR data for *trans*-P4 fibrils; S9, summary of all MD simulation results; S10, electrostatic potential surface of PTH_25–37_ and selected peptides; S11, absorption data to exclude photobleaching; S12, cell viability assay of *trans*-P4, *trans*-P8, and *trans*-P12; S13–S20, ThT fluorescence data; S21–S27, TEM images of fibrils; S28, CD spectra during reversible fibrillization of P4; S29, UV–vis spectra of *trans*-P4 before each fibrillization cycle; S30–S47, ^1^H NMR, HPLC traces, and MALDI data of the peptides P1–P12. **Supplementary tables** S1, list of all MD simulations performed; S2, sequence of PTH_25–37_ and P1–P12 and their respective solubility; S3, photophysical properties of peptides; S4: fibrillization parameters of peptides (PDF)

## Abbreviations

AMPB: 3-{[(4-aminomethyl)phenyl]diazenyl}benzoic acid
CD: circular dichroism
MD: molecular dynamics
NMR: nuclear magnetic resonance
PSS: photostationary state
PTH: parathyroid hormone
ssNMR: solid state nuclear magnetic resonance
TEM: transmission electron microscopy
ThT: thioflavin T
UV/vis: ultraviolet/visible
WAXS: wide-angle X-ray scattering
