# Supplementary material for: Photocontrolled Reversible Amyloid Fibril Formation of Parathyroid Hormone-Derived Peptides
Source: Bioconjug Chem. 2024 Jun 12;35(7):981–95. doi: 10.1021/acs.bioconjchem.4c00188 (PMC11261605; doi:10.1021/acs.bioconjchem.4c00188)
Supplement: Supplementary file 1 — bc4c00188_si_001.pdf [file bc4c00188_si_001.pdf]

Supporting Information

Photocontrolled reversible amyloid fibril formation of parathyroid hormone-derived peptides

André Paschold[1,#], Moritz Schäffler[2,3,#], Xincheng Miao[4], Luis Gardon[2,5], Stephanie Krüger[6], Henrike Heise[2,5], Merle Insa Silja Röhr[4], Maria Ott[7], Birgit Strodel[2, 3]*, Wolfgang H. Binder[1]*

[1] Macromolecular Chemistry, Institute of Chemistry, Faculty of Natural Science II, Martin Luther University Halle Wittenberg, von-Danckelmann-Platz 4, 06120 Halle (Germany)

[2] Institute of Theoretical and Computational Chemistry, Heinrich Heine University Düsseldorf, 40225 Düsseldorf, Germany

[3] Institute of Biological Information Processing, Structural Biochemistry (IBI-7), Forschungszentrum Jülich, 52425 Jülich, Germany

[4] Center for Nanosystems Chemistry (CNC), Theodor-Boveri Weg, Universität Würzburg, 97074 Würzburg, Germany

[5] Institute of Physical Biology, Heinrich-Heine-Universität Düsseldorf, 40225 Düsseldorf, Germany

[6] Biozentrum, Martin Luther University Halle-Wittenberg, Weinberweg 22, 06120 Halle (Germany)

[7] Institute of Biophysics, Faculty of Natural Science I, Martin Luther University Halle-Wittenberg, Kurt-Mothes-Straße 3, 06120 Halle (Germany)

[#] These authors contributed equally to this work.

Contents

1. Supplementary figures 2
2. Supplementary tables 19
3. Peptide characterization after synthesis 21

1. Supplementary figures

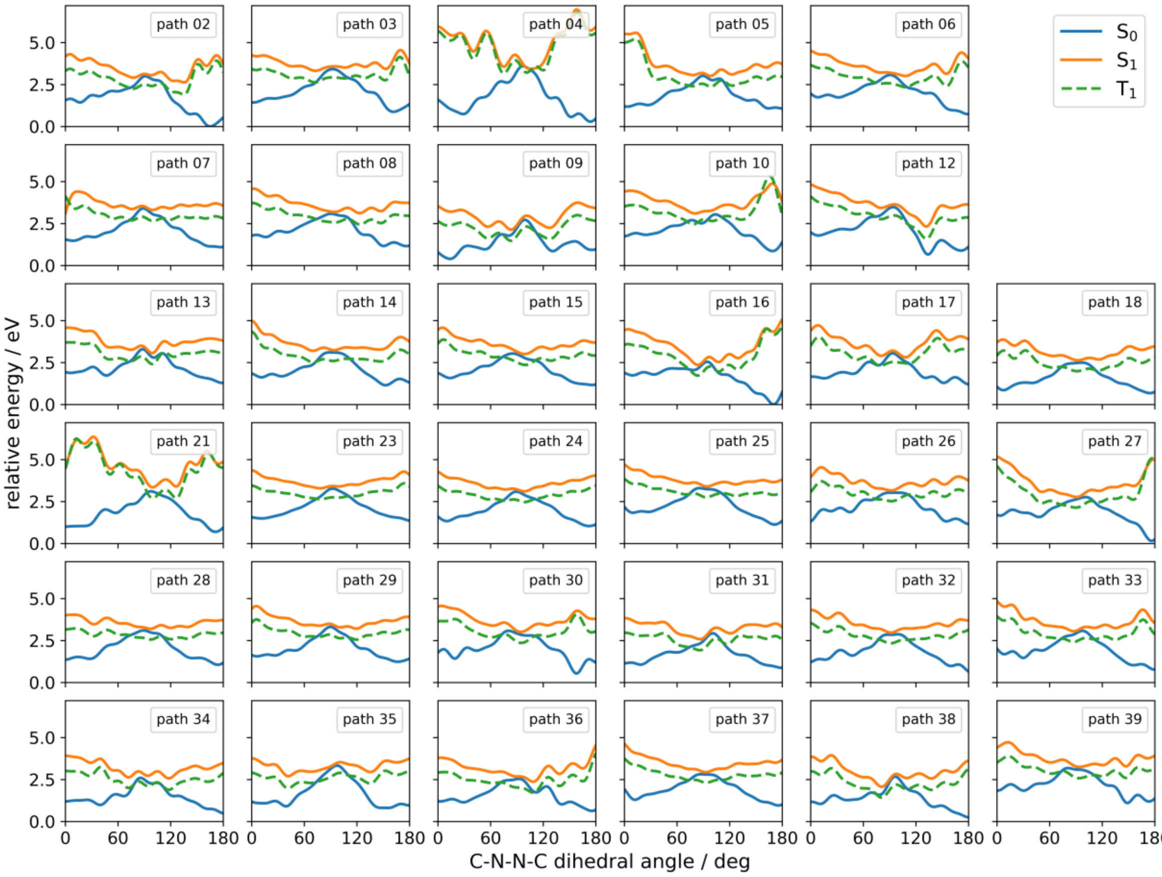

Figure S1. The smoothed potential energy curves from 34 converged *cis* ↔ *trans* isomerization paths. Single-point calculations are performed using NEVPT2(2,2)/xTB with implicit water through ALPB. The smoothing is carried out using Gaussian process regression. A subset of the paths has an energy barrier in the *trans*-*cis*-isomerization, notably paths 04, 10 and 21, with excitation energies before the barrier range from 2.5 to 3.0 eV. This correlates with the experimental fluorescence peak at 485 nm (**Figure S2**), albeit with an expected ipsochromic effect caused by the minimum active space considered in the calculations. We attribute the extended fluorescence lifetime, compared to azobenzene, to structures hindered by this barrier, preventing radiationless relaxation. These barriers predominantly arise from structures characterized by dihedral angles around 170°, which exhibit a heightened S0–S1 excitation energy in the scan.

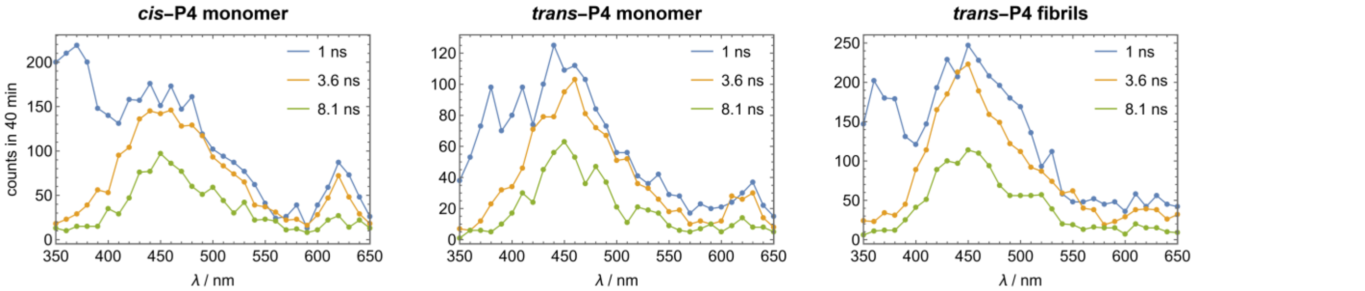

Figure S2. Fluorescence life-time measurements. Fluorescence spectra measured at various points in time after the excitation pulse.

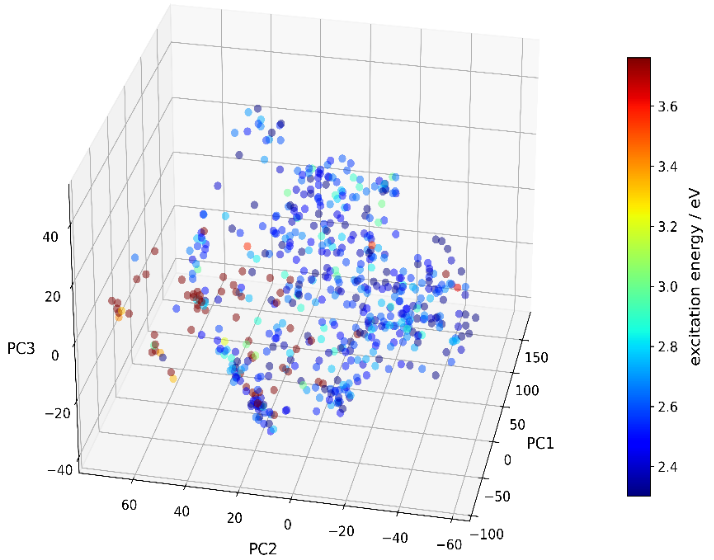

Figure S3. All geometries with dihedral angles near 170° projected on to the three-dimensional PCA space. The colors encode the S0–S1 excitation energies. The reduced three-dimensional space accounts for 86% of the total variance. Several clusters can be detected, notably one predominantly comprising structures with high excitation energies (visually represented in dark red in the lower left corner of the projection). This particular cluster is positioned at the negative extremity of the PC1-axis, advances towards the positive end of the PC2-axis and is situated approximately mid-way along the PC3-axis.

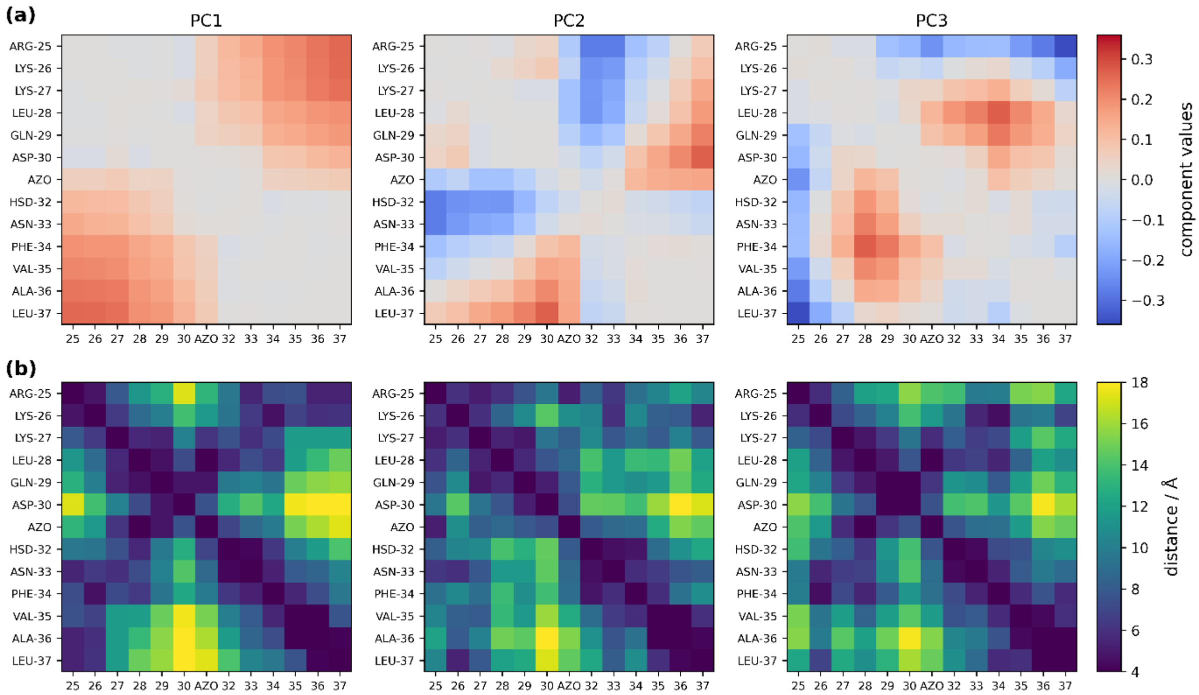

Figure S4. (a) The first three principal components represented in the distance matrix space. The first principal component signals a considerable separation between the residues R25 and A36/L37. Given this cluster's positioning at the PC1-axis's negative end, it implies that within these geometries, the distances between these residues are notably reduced. The second principal component reveals negative values for the separation between R25 and H32/N33, alongside positive distances between D30 and L37. The positive projection of this cluster on the PC2 suggests these distance matrix characteristics directly, without reversing the sign. With almost negligible projection on PC3, this component scarcely influences the distance matrices for structures within this cluster. (b) Distance matrices for clusters with high excitation energies. Despite originating from different paths, these matrices exhibit consistent patterns.

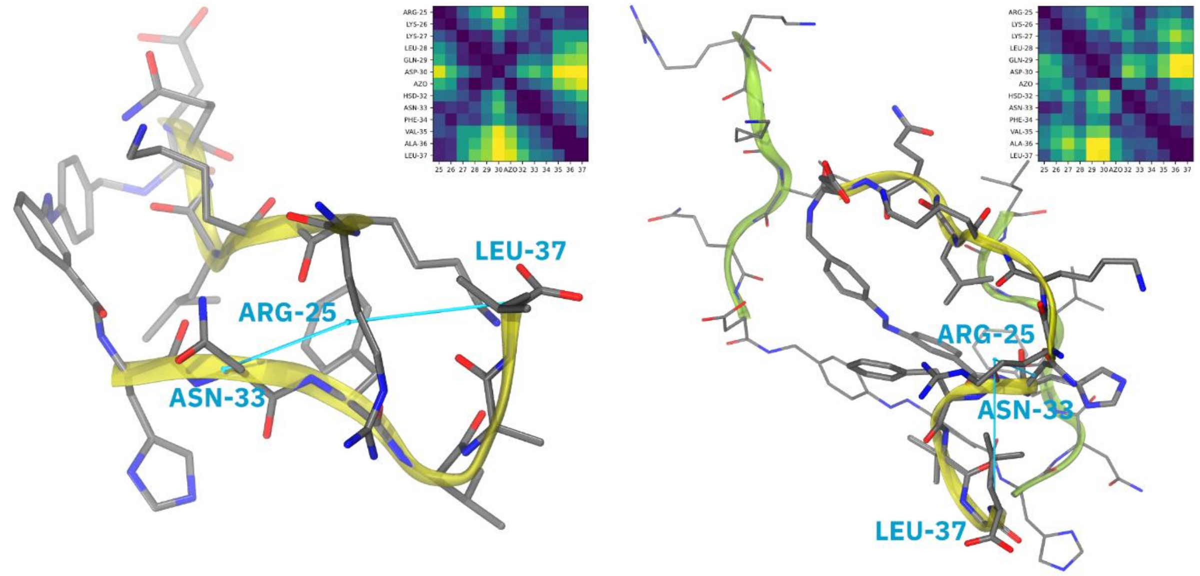

Figure S5. Selected monomer structure (left) with distance matrix shown in Figure S4b and dimer structure (right) with a similar distance matrix. The corresponding distance matrices are shown in the upper right corners. It can be seen that the simultaneous interactions of R25 with A36/L37 and H32/N33 potentially contribute to the S1-barrier during the *trans*→*cis* isomerization path, leading to longer fluorescence lifetimes. This structural motif is also found in the dimer, while their configurations stabilized by intermolecular interactions could also lead to an increased barrier.

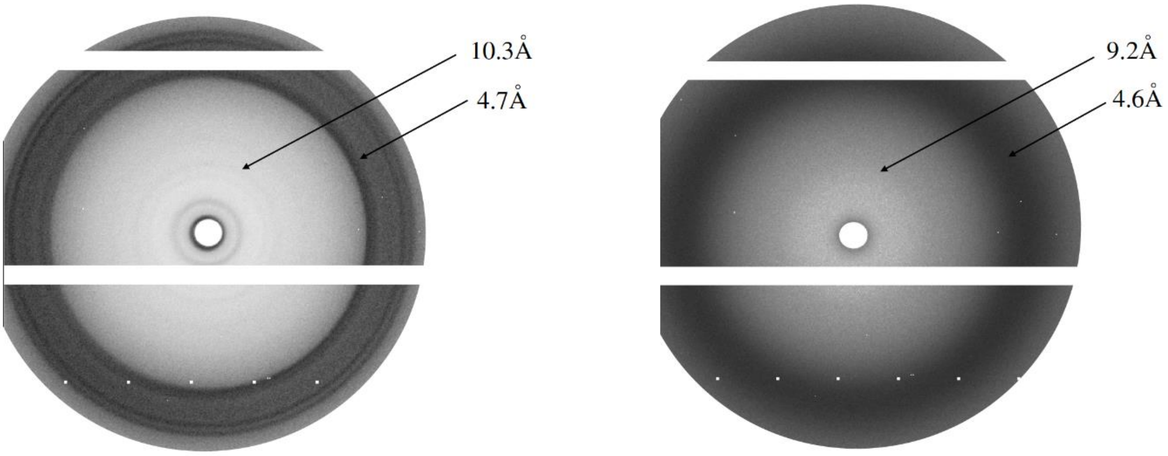

Figure S6: Wide-angle x-ray diffraction patterns from dried fibrillar samples of $PTH_{25\text{-}37}$ (left) and P4 (right).

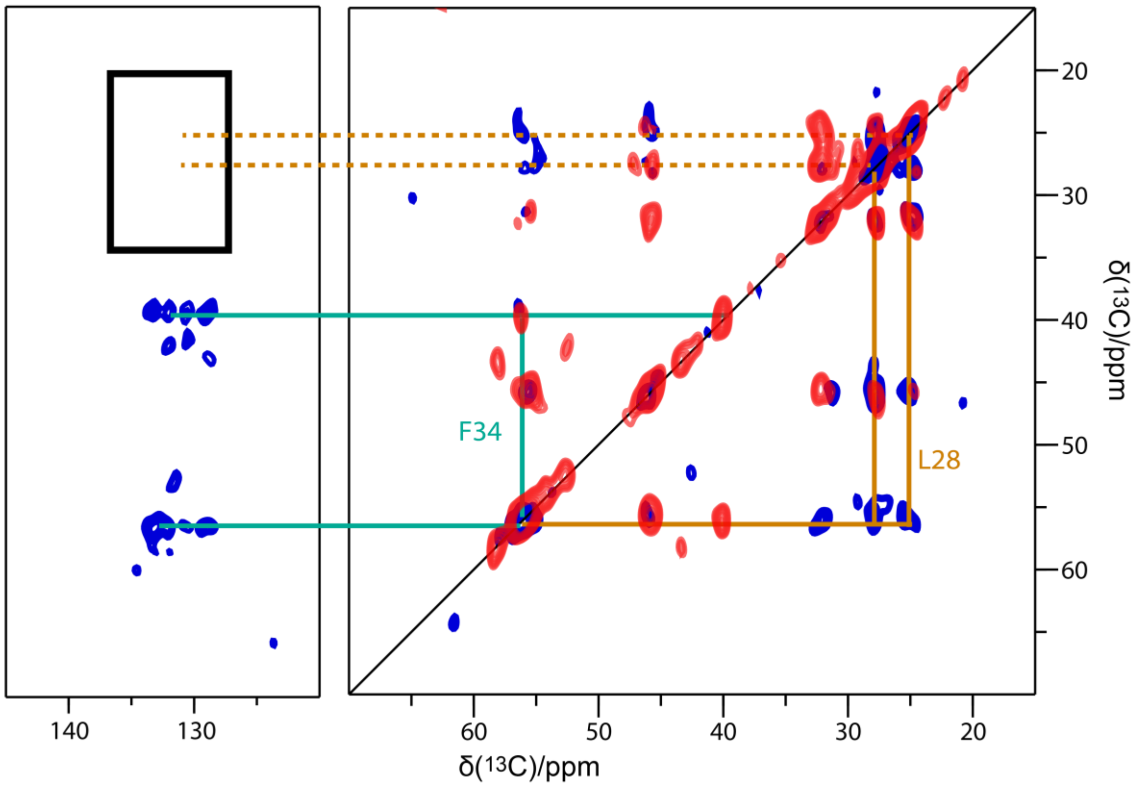

Figure S7. 2D ^{13}C,^{13}C spin diffusion spectra of fibrils from PTH_{25-37}, uniformly ^{13}C labeled for L28 and F34, recorded under different conditions. Red: magnetic field strength 14.1 T (corresponding to 600 MHz ^{1}H resonance frequency), spinning speed 11 kHz, mixing time 10 ms. Blue: magnetic fiels strength of 18.8 T (corresponding to 800 MHz ^{1}H resonance frequency), spinning frequency of 20 kHz, mixing time 1 s. Figure S6 shows the 2D ^{13}C,^{13}C spin diffusion spectra of fibrils from PTH_{25-37}, uniformly ^{13}C labeled for L28 and F34, recorded under different conditions. In the red spectrum, intraresidual cross-peaks between neighboring ^{13}C sites of L28 and F34, respectively, are visible. At a longer mixing time of 1 s (blue), long-range correlations between all ^{13}C spins within one residue are obtained. Spin systems of the labeled amino acids F34 (cyan) and L28 (brown) are marked by solid lines. Dashed lines show a possible contact between L28 Cγ/Cδ and an aromatic F34 carbon. The spinning speed of 20 kHz corresponds to the first order rotational resonance condition for resonances with a chemical shift difference of 100 ppm, leading to a recoupling of dipolar couplings between aromatic ring carbon atoms of F34 and aliphatic C_γ and C_δ signals of L28, facilitating magnetization transfer between those residues if the distance between these residues would not exceed 6 Å.[20] The fact that no inter-residual cross-peaks between L28 and F34 can be observed is thus a strong indication against an antiparallel arrangement of β-strands within the β-sheet.

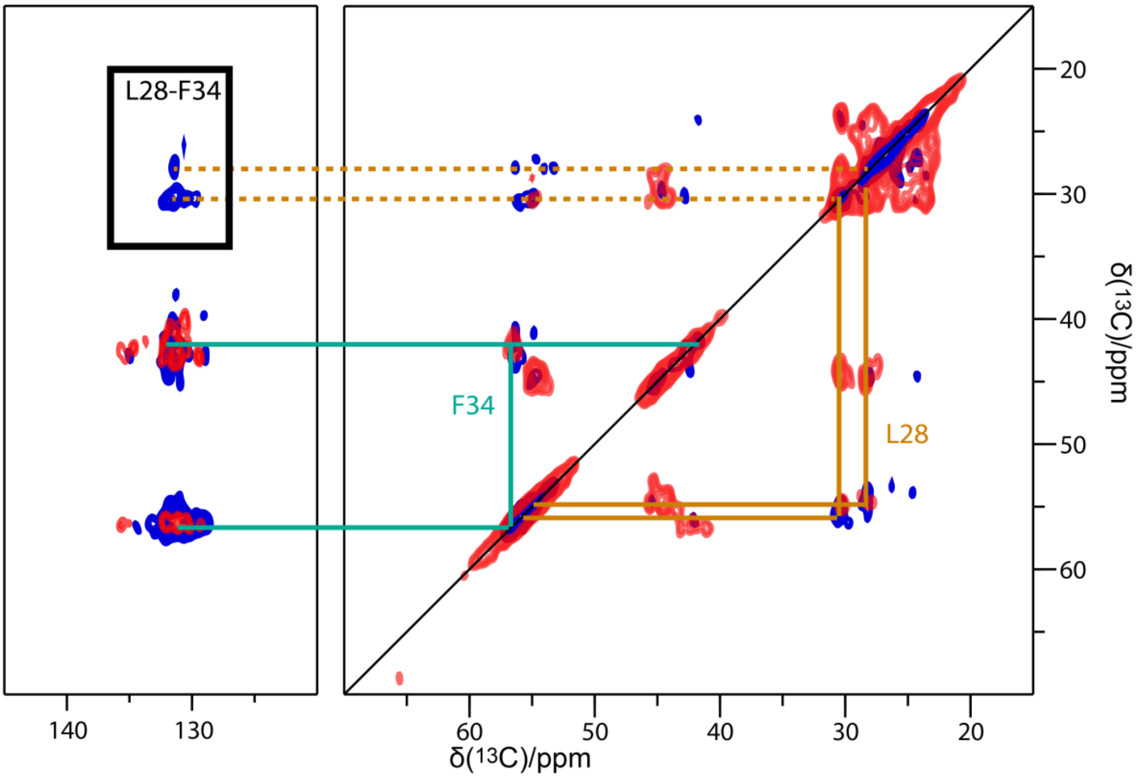

Figure S8. 2D ^{13}C,^{13}C spin diffusion spectra of fibrils from P4, uniformly ^{13}C labeled for L28 and F34, recorded at a magnetic field strength of 18.8 T (corresponding to 800 MHz ^{1}H resonance frequency) at a spinning speed of 20 kHz, corresponding to the first order rotational resonance condition for signals with a chemical shift difference of 100 ppm. Red: Mixing time of 50 ms. Blue: Mixing time of 1 s. See for more information in Fig. S6. Here, inter-residual cross-peaks between the aromatic ring signals of F and L Cδ resonances are clearly visible for a mixing time of 1 s (blue spectrum).

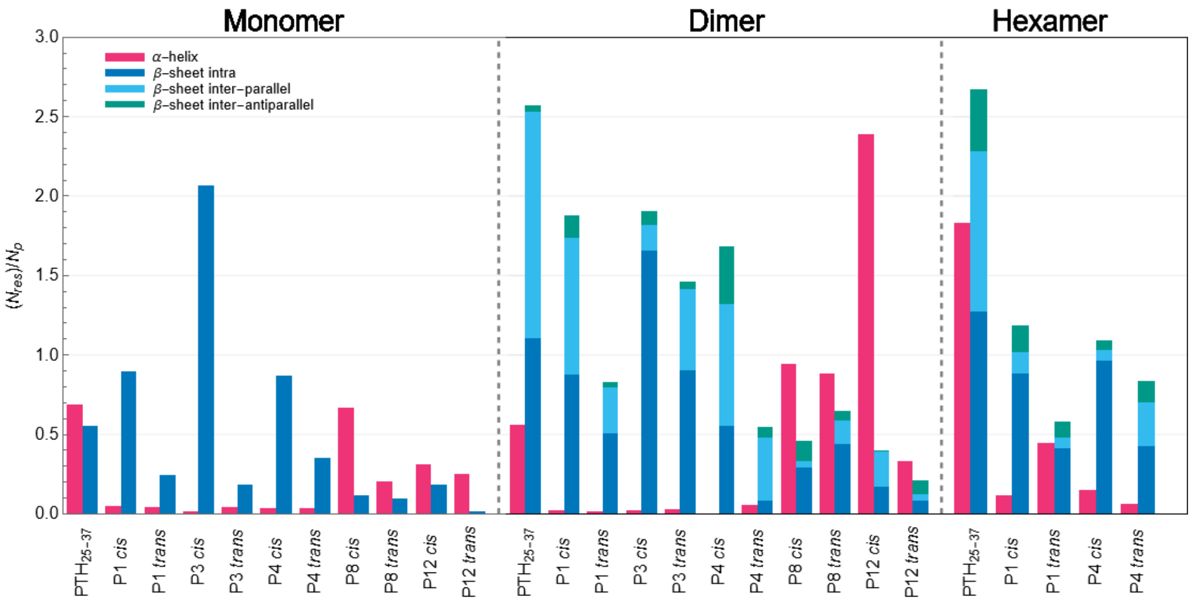

Figure S9. Summary of all MD simulation results. The average numbers of residues $\langle N_{\mathrm{res}} \rangle$ forming secondary structure elements, divided into α-helical (magenta), intrapeptide β-sheets (blue), interpeptide parallel β-sheets (cyan) and interpeptide antiparallel β-sheets (green) are given. The average was taken over the number of frames of the simulation and normalized by the number of peptides N_P present in the corresponding simulation.

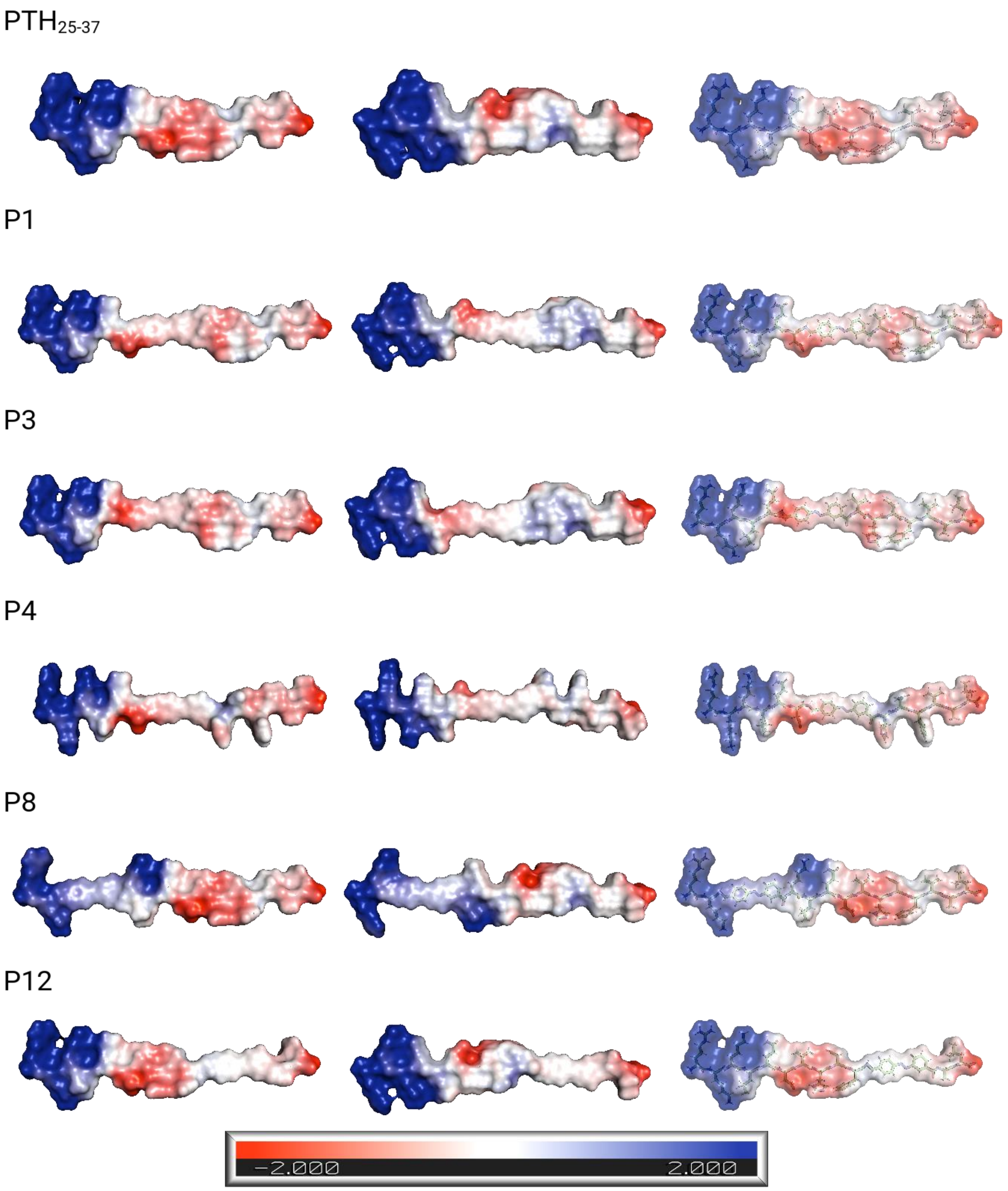

Figure S10. Electrostatic potential surface of PTH_{25-37} and the peptides P1, P3, P4, P8, and P12, with values according to the color map at the bottom, ranging from -2 (red) to +2 kTe^{-1} (blue). The electrostatic potential mapped to the molecular surfaces was calculated using the Adaptive Poisson-Boltzmann Solver (APBS[1]) plugin for the pymol[2] software package. For each peptide, the two views that are rotated by 180° around the backbone axis are shown, as well as a transparent front view.

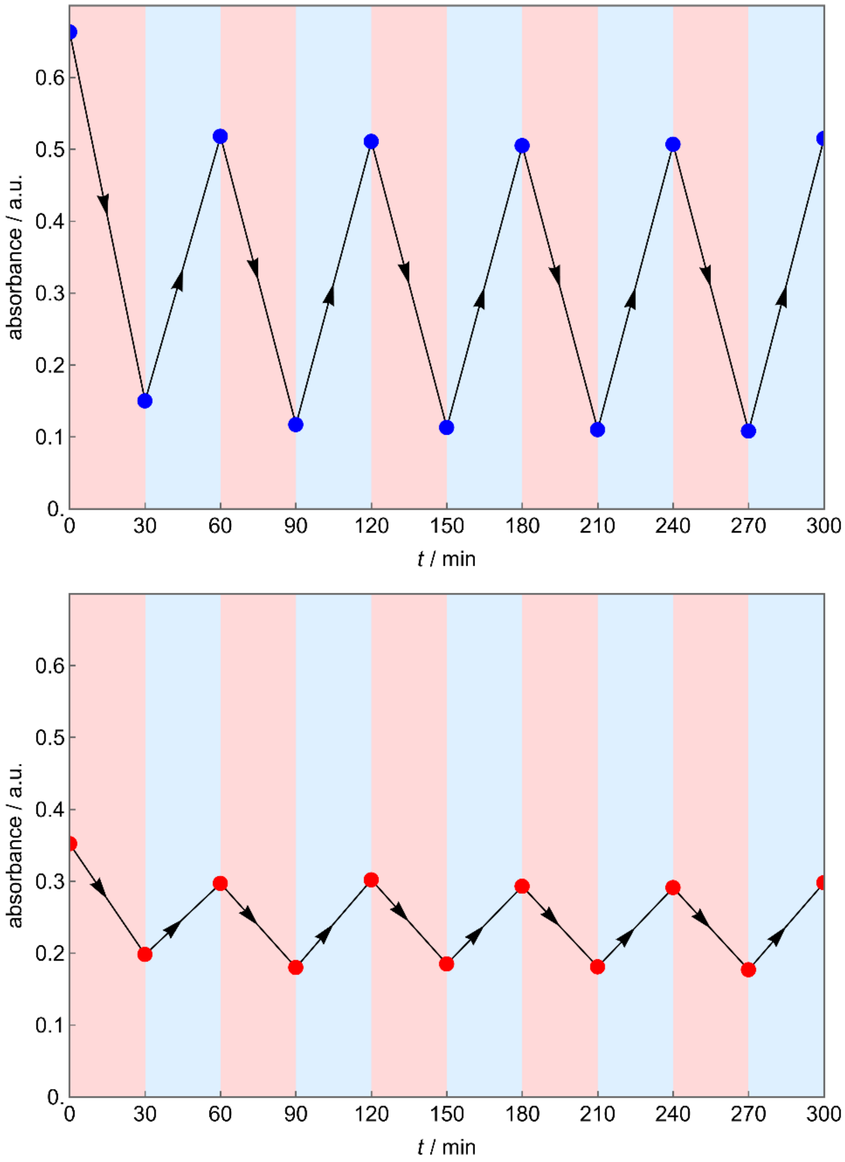

Figure S11: Control experiment to investigate photobleaching of the azobenzene unit during the photoisomerization. Absorption of P4 was measured after each isomerization step at 327 nm (top, blue dots) and 295 nm (bottom, red dots). Light red area corresponds to irradiation with light of 340 nm wavelength for 30 min to achieve *trans*→*cis* isomerization. Light blue area corresponds to irradiation with light of 405 nm wavelength for 30 min to achieve *cis*→*trans* isomerization.

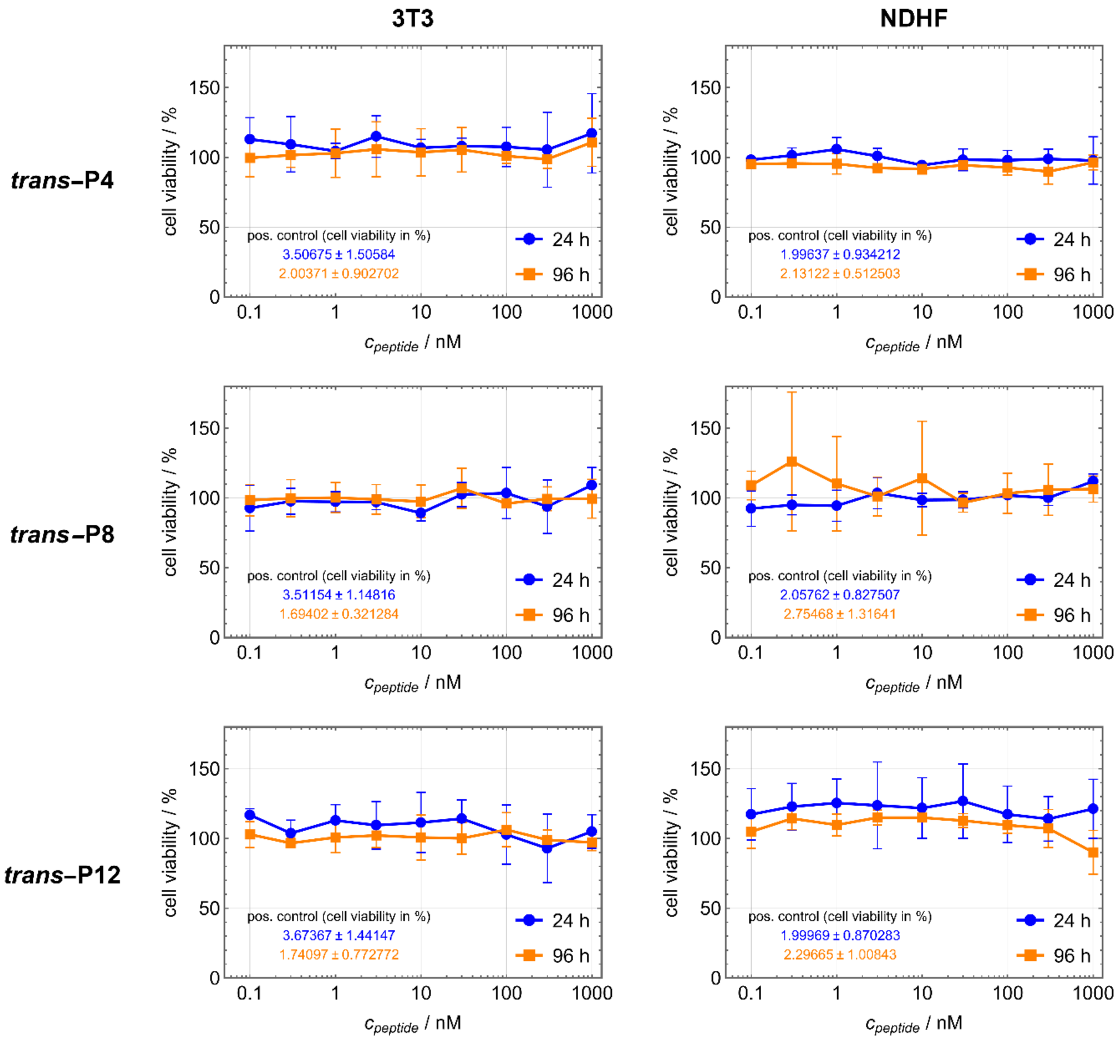

Figure S12: Cell viability assay of *trans*-P4, *trans*-P8, and *trans*-P12 on NHDF (human, adult, fibroblasts) and 3T3 (murine, embryonal, fibroblasts) cells after 24 h (blue) and 96 h (orange). 100% cell viability corresponds to the value of the negative, untreated control.

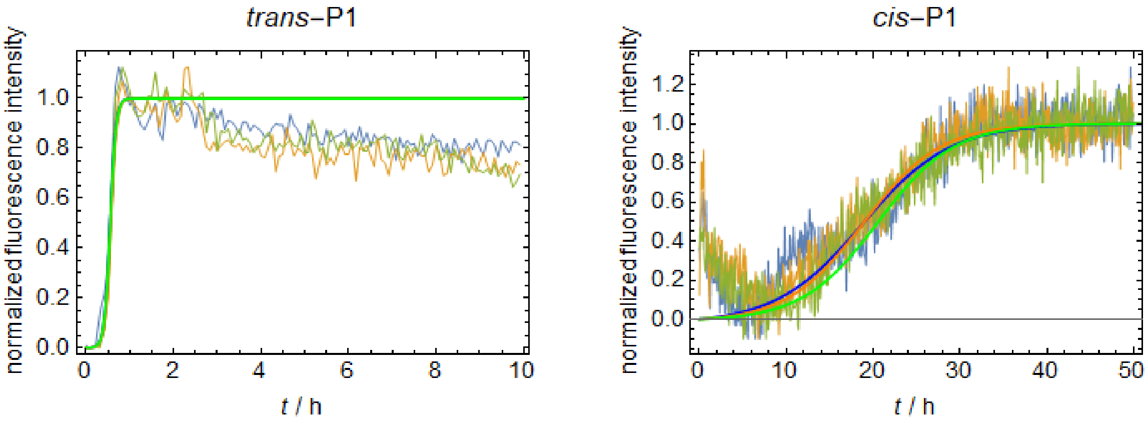

Figure S13. Fibrillization kinetics of 85 µM P1 as *trans*- (left) and *cis*-isomer (right). Measured in a ThT-monitored fluorescence assay at 37 °C in 50 µM Na_2HPO_4 buffered aqueous solution (pH 7.4).

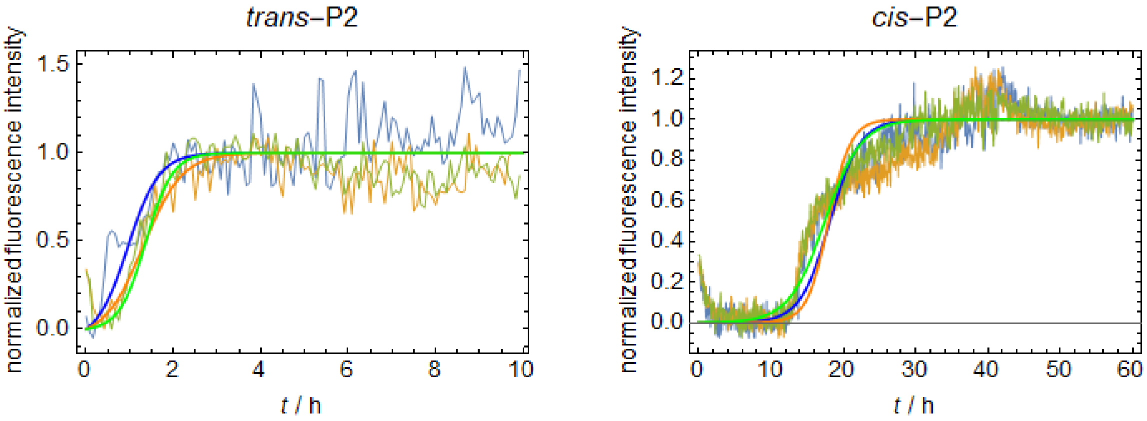

Figure S14. Fibrillization kinetics of 55 µM P2 as *trans*- (left) and *cis*-isomer (right). Measured in a ThT-monitored fluorescence assay at 37 °C in 50 µM Na_2HPO_4 buffered aqueous solution (pH 7.4).

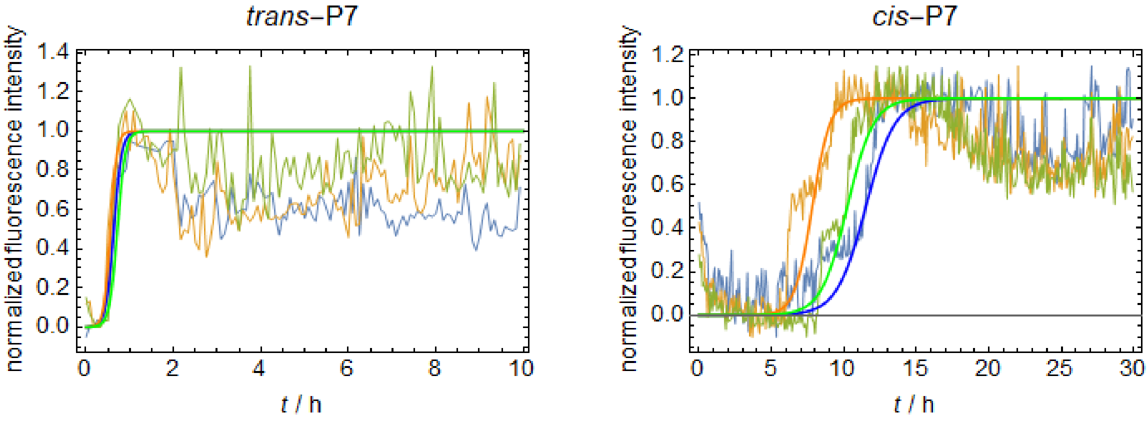

Figure S15. Fibrillization kinetics of 100 µM P7 as *trans*- (left) and *cis*-isomer (right). Measured in a ThT-monitored fluorescence assay at 37 °C in 50 µM Na_2HPO_4 buffered aqueous solution (pH 7.4).

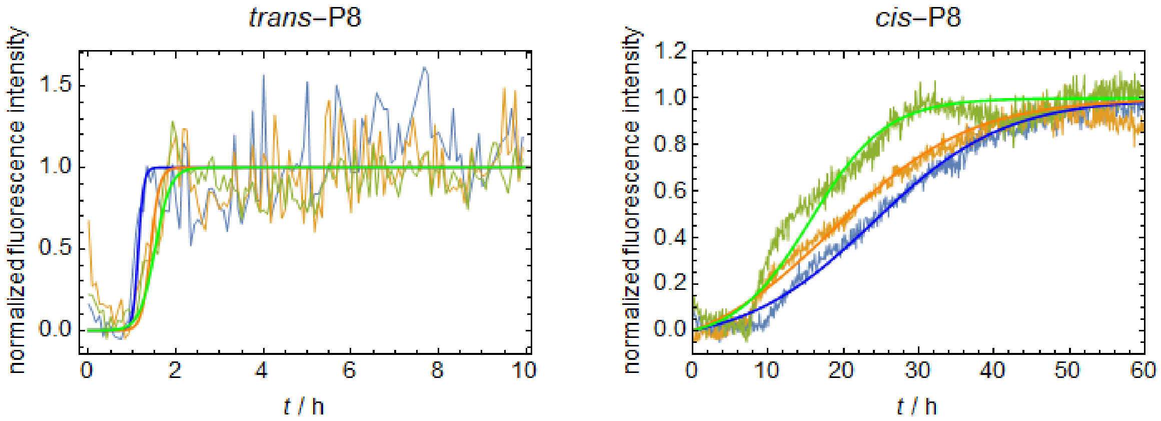

Figure S16. Fibrillization kinetic of 100 µM P8 as *trans*- (left) and *cis*-isomer (right). Measured in a ThT-monitored fluorescence assay at 37 °C in 50 µM Na_2HPO_4 buffered aqueous solution (pH 7.4).

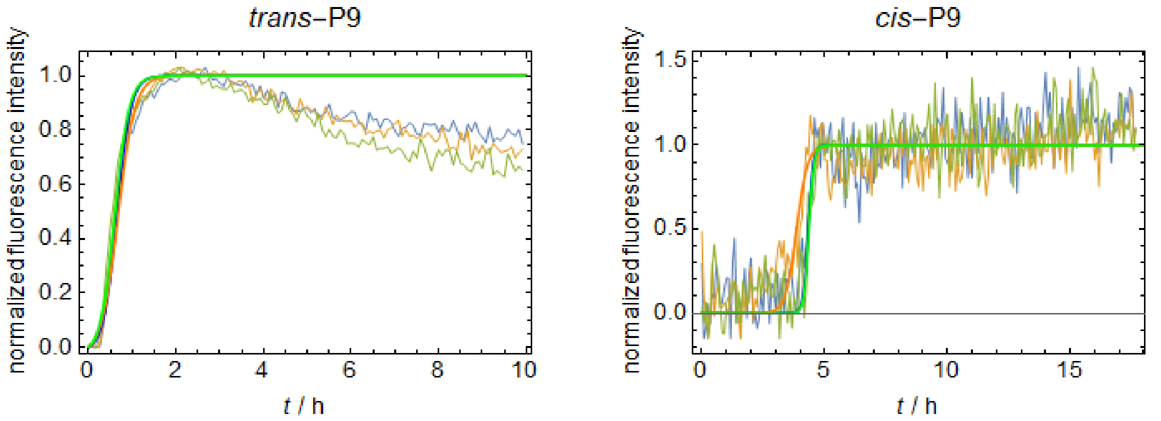

Figure S17. Fibrillization kinetics of 100 µM P9 as *trans*- (left) and *cis*-isomer (right). Measured in a ThT-monitored fluorescence assay at 37 °C in 50 µM Na_2HPO_4 buffered aqueous solution (pH 7.4).

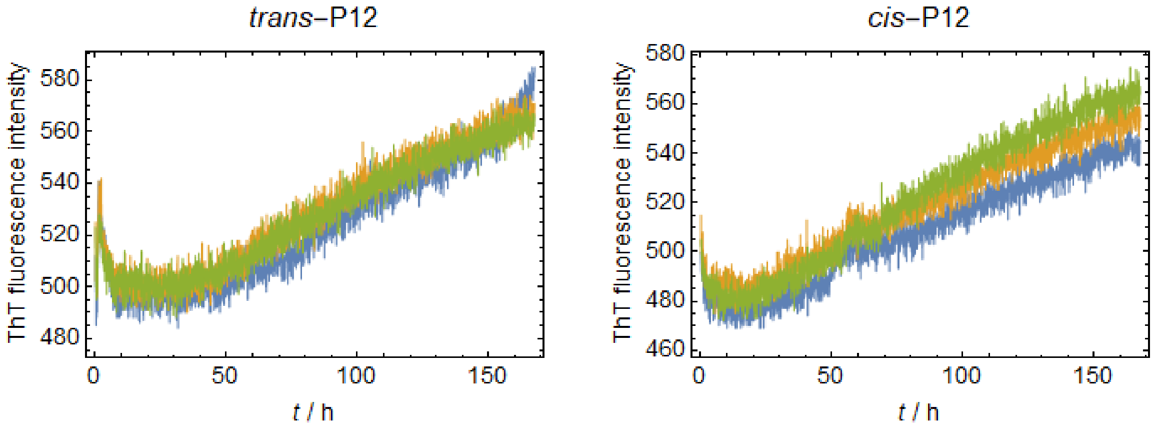

Figure S18. Fibrillization kinetics of 100 µM P12 as *trans*- (left) and *cis*-isomer (right). Measured in a ThT-monitored fluorescence assay at 37 °C in 50 µM Na_2HPO_4 buffered aqueous solution (pH 7.4).

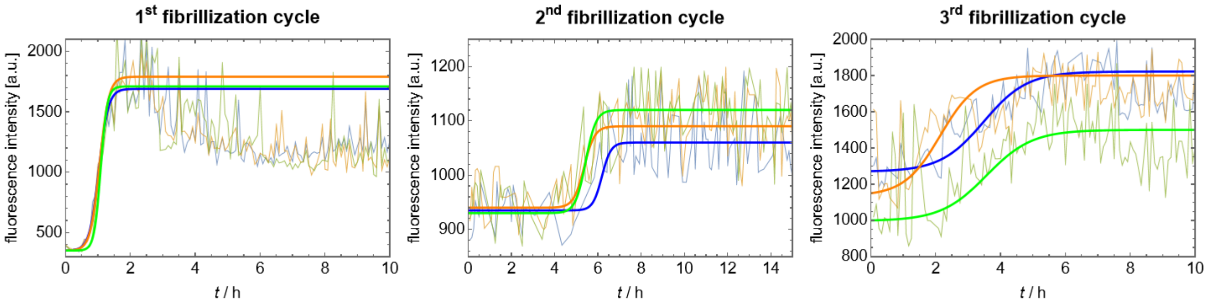

Figure S19. Fibrillization kinetics of P4 over three cycles of alternating fibrillization and fibril degradation through *trans*→*cis* isomerization measured in a ThT-monitored fibrillization assay at 37 °C in 50 µM Na_2HPO_4 buffered aqueous solution (pH 7.4).

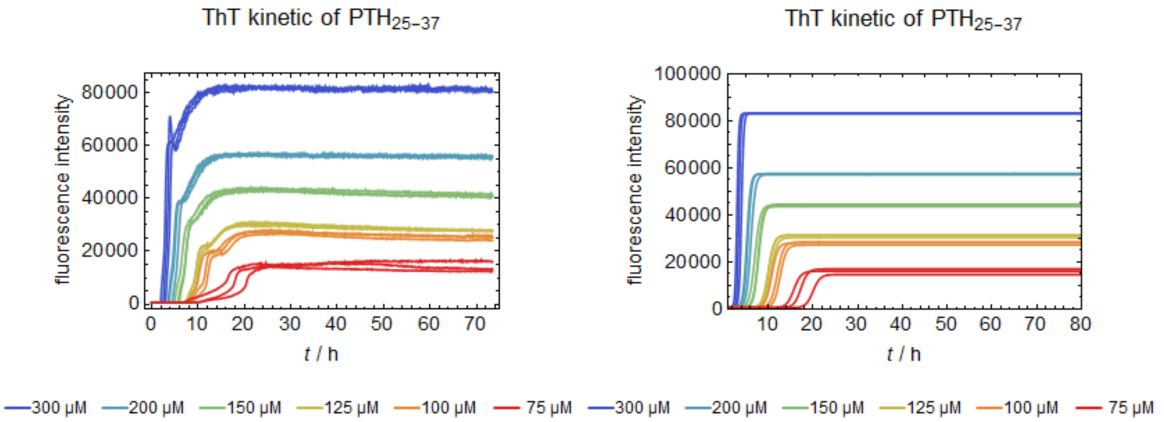

Figure S20. Fibrillization kinetics of $PTH_{25\text{-}37}$ in different concentrations measured in a ThT-monitored fluorescence assay at 37 °C in 50 µM Na_2HPO_4 buffered aqueous solution (pH 7.4); (left) raw data, (right) fitted data using Eq. (1).

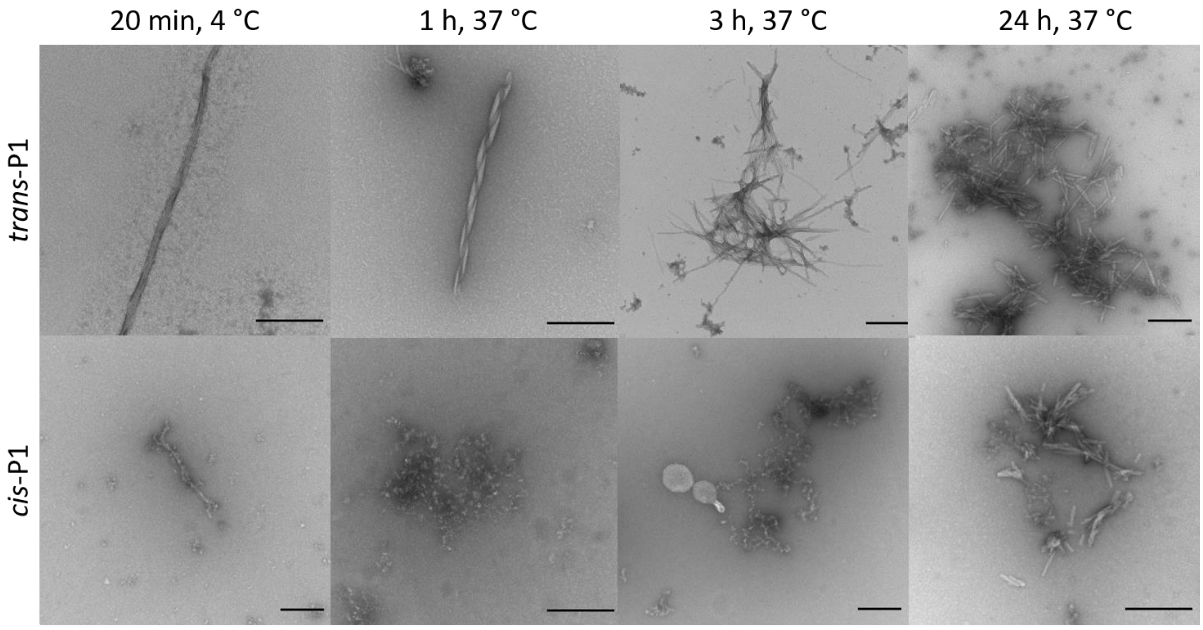

Figure S21. TEM images of P1 at different times and temperatures; scale bar = 250 nm.

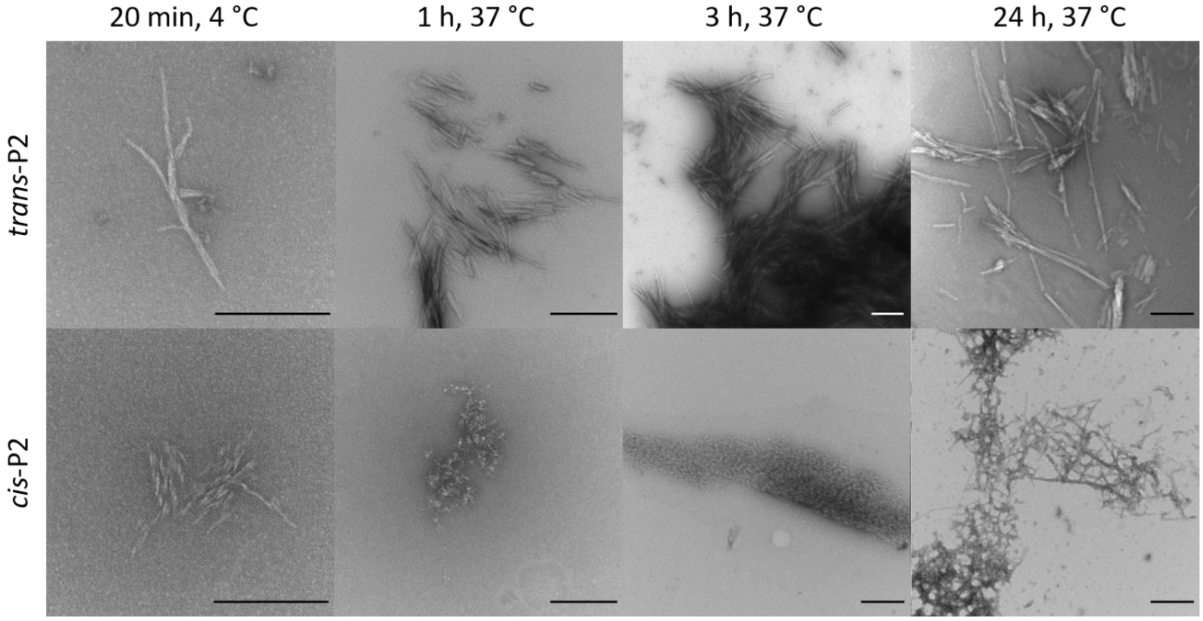

Figure S22. TEM images of P2 at different times and temperatures; scale bar = 250 nm.

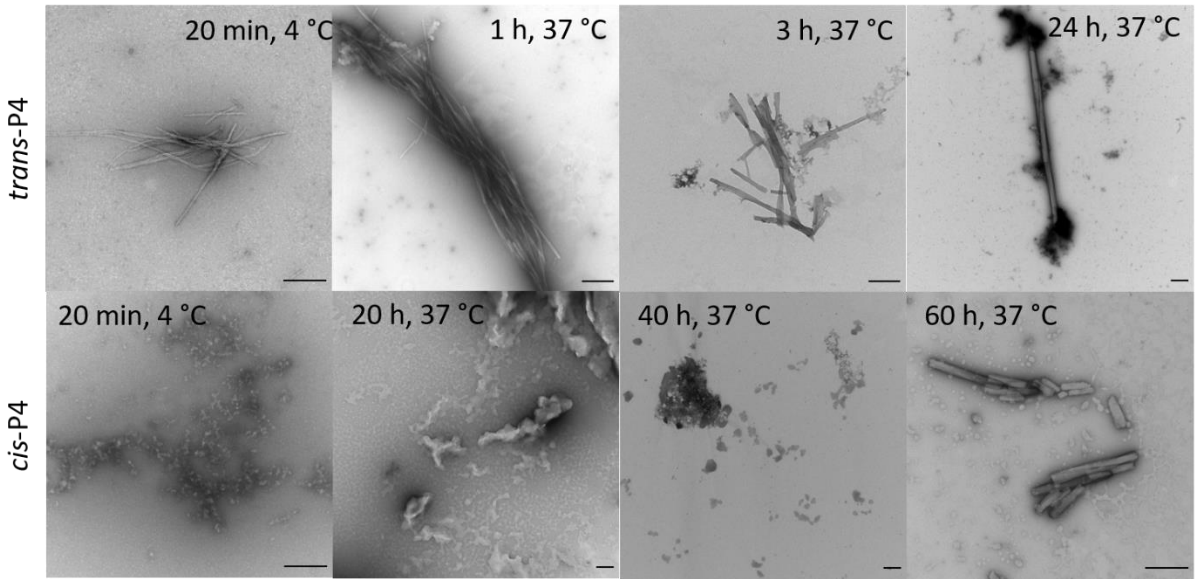

Figure S23. TEM images of P4 at different times and temperatures; scale bar = 250 nm.

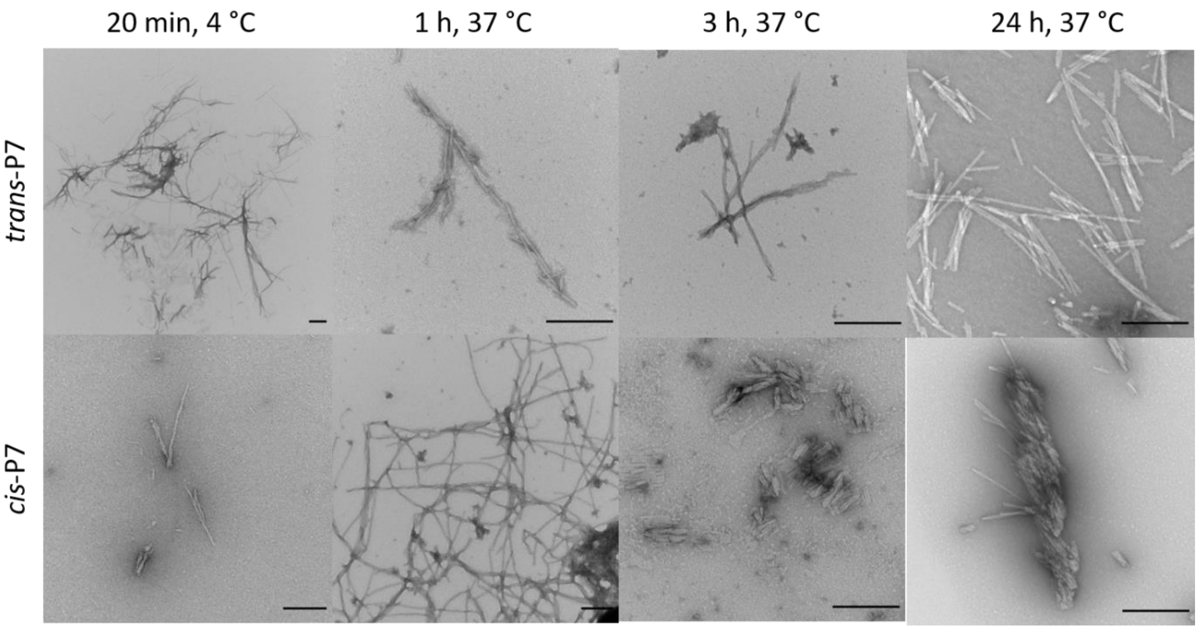

Figure S24. TEM images of P7 at different times and temperatures; scale bar = 250 nm.

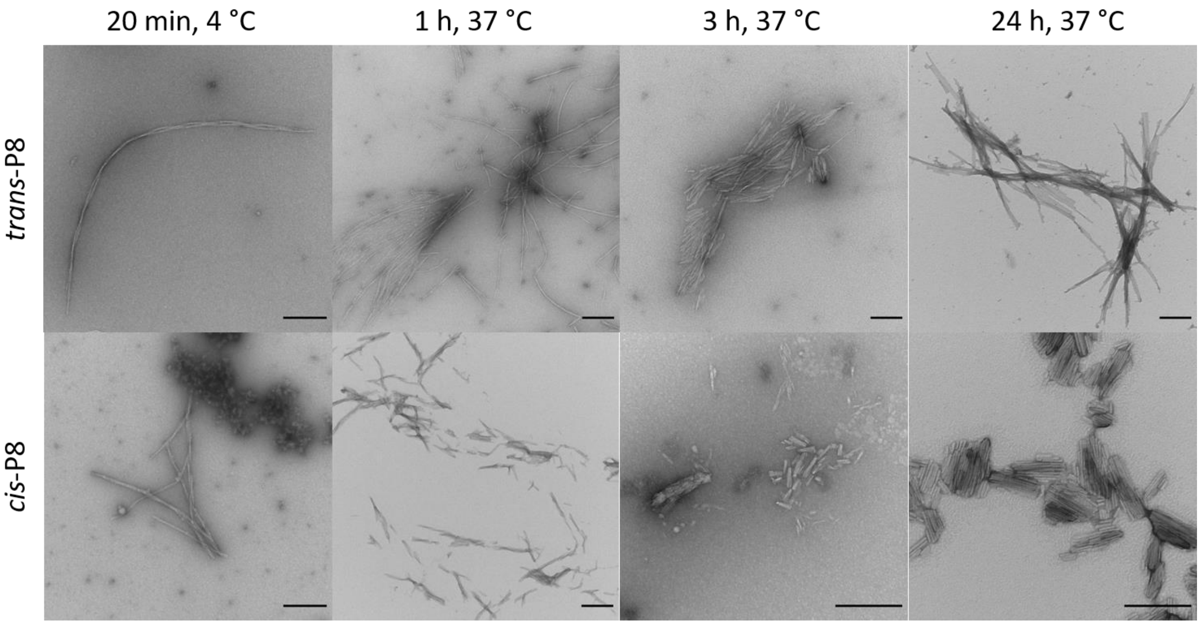

Figure S25. TEM images of P8 at different times and temperatures; scale bar = 250 nm.

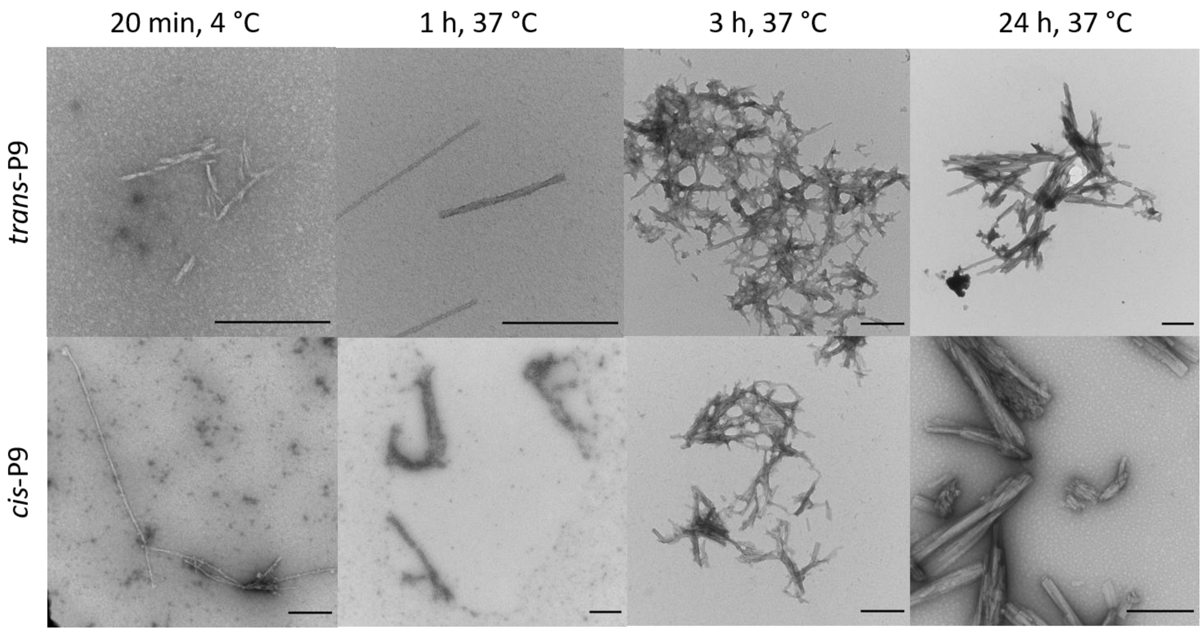

Figure S26. TEM images of P9 at different times and temperatures; scale bar = 250 nm.

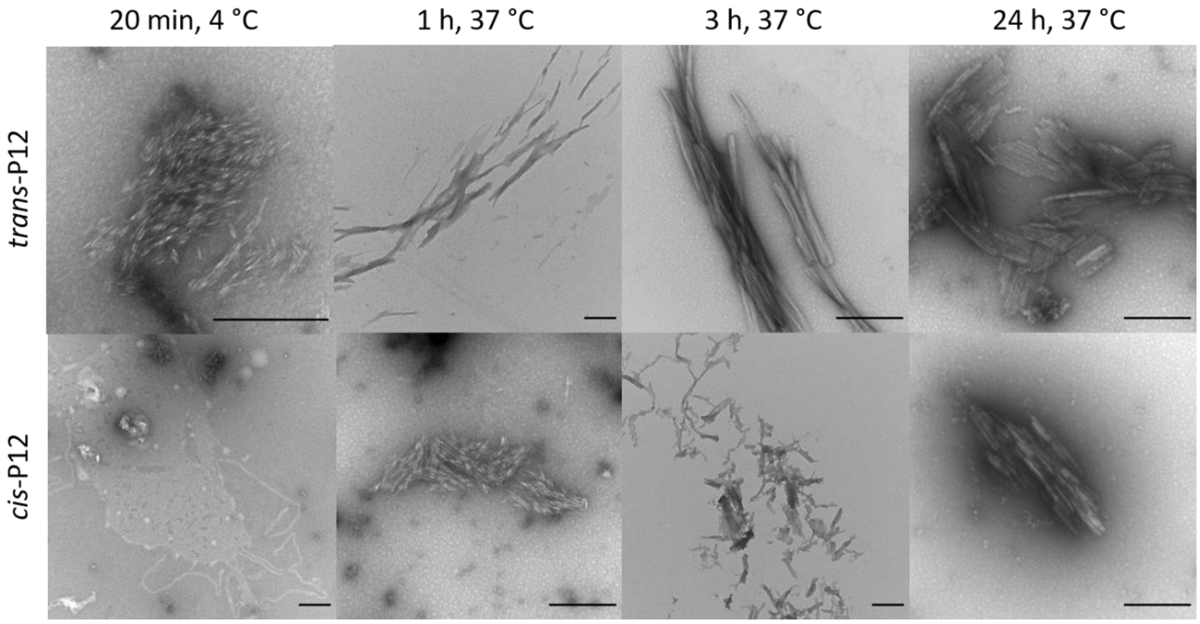

Figure S27. TEM images of P12 at different times and temperatures; scale bar = 250 nm.

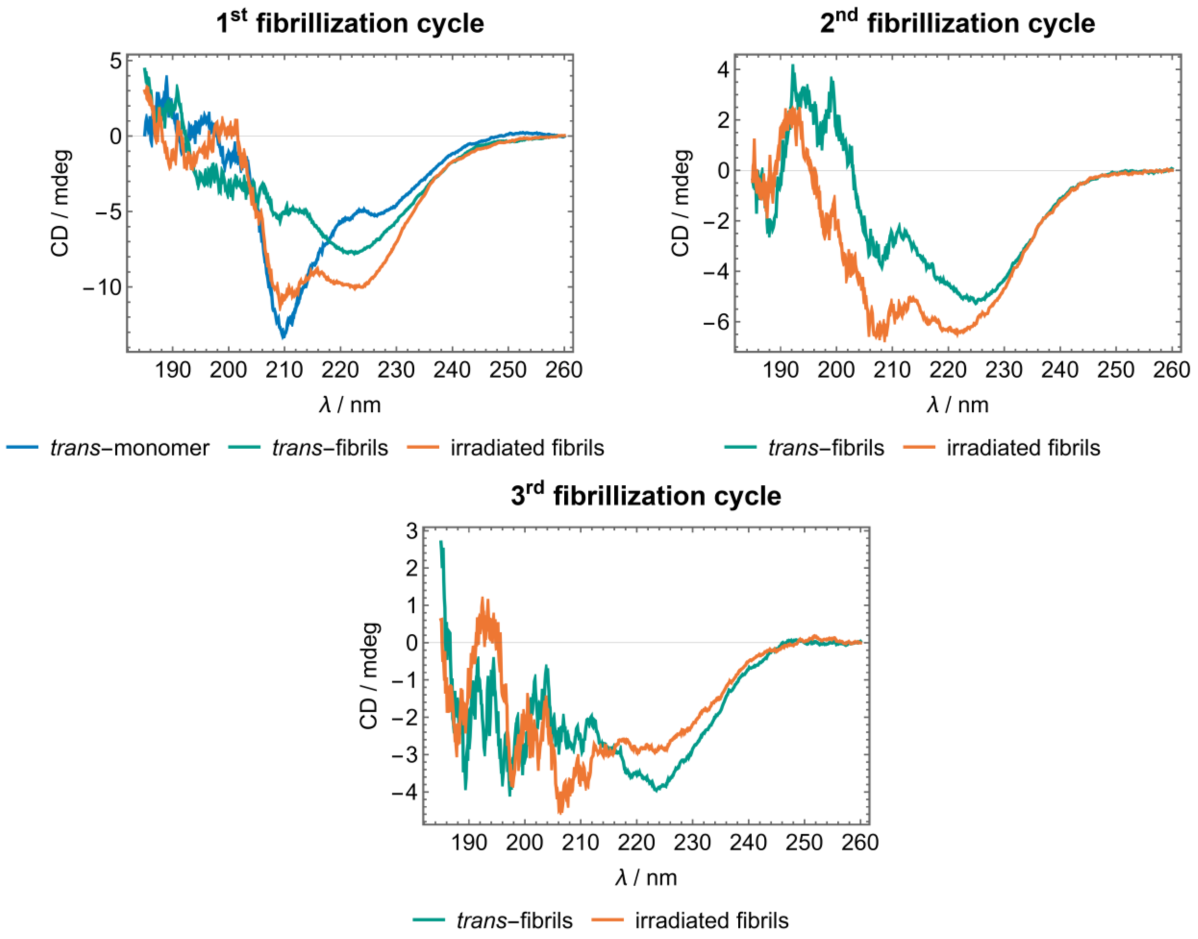

Figure S28: CD-spectra of the reversible fibrillization of P4 over three cycles (1st, 2nd, and 3rd). *trans*-Isomer (blue) was measured directly after dissolving the peptide, *trans*-fibrils (green) were measured 20 h after reference sample reached the stationary phase, and irradiated fibrils (orange) were measured directly after irradiating the fibrils with 340 nm for 5 h.

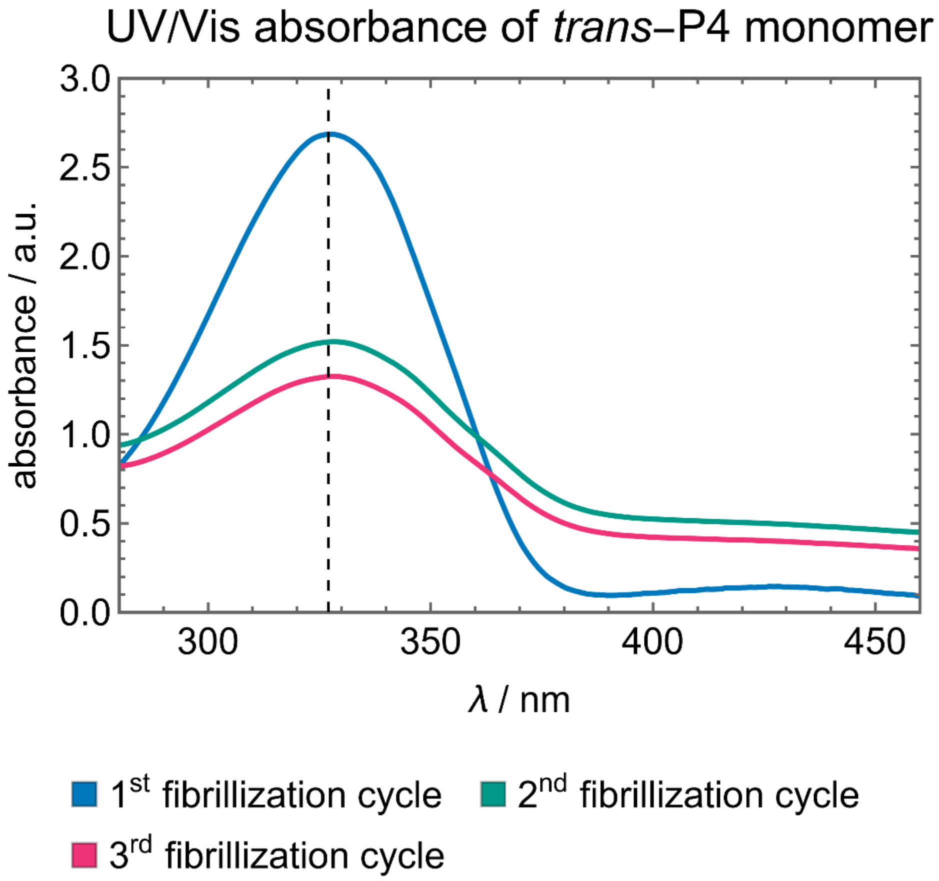

Figure S29: UV/Vis-spectra of *trans*-P4 monomer before each fibrillization cycle. Dashed line corresponds to the absorption maxima of the *trans*-isomer at 327 nm.

2. Supplementary tables

Table S1. All Simulations performed, with their respective simulation time

Peptide	Simulation	Simulation Time [µs]
$PTH_{25\text{-}37}$	Monomer/Dimer/Hexamer	3 systems x 10
P1	Monomer/Dimer/Hexamer - *cis*/*trans*	6 systems x 10
P3	Monomer/Dimer - *cis*/*trans*	4 systems x 10
P4	Monomer/Dimer/Hexamer - *cis*/*trans*	6 systems x 10
P8	Monomer/Dimer - *cis*/*trans*	4 systems x 10
P12	Monomer/Dimer - *cis*/trans	4 systems x 10
$PTH_{25\text{-}37}$	fibril	4 models x 1
P4	fibril	11 models x 1

Table S2. Sequence of $PTH_{25\text{-}37}$ and P1 – P12 and their respective solubility in buffered solution (50 mM aqueous Na_2HPO_4) with pH 7.4, the critical fibrillization concentration (c_{cr}), and standard free energy of the fibrillization reaction ΔG^0.

Peptide	Primary sequence	Solubility [µM]	c_{cr} [µM]	ΔG^0 [kJ/mol]
$PTH_{25\text{-}37}$	^{25}RKKLQ^{30}DVHNF^{35}VAL	>500	42	26
P1	^{25}RKKLQ^{30}D-Azo-VHNF^{35}VAL	90	28	27
P2	^{25}RKKLQ^{30}DV-Azo-HNF^{35}VAL	60	27	27
P3	^{25}RKKLQ-Azo-VHNF^{35}VAL	35	n.d.	n.d.
P4	^{25}RKKLQ^{30}D-Azo-HNF^{35}VAL	370	23	28
P5	^{25}RKKLQ-Azo-HNF^{35}VAL	25	n.d.	n.d.
P6	^{25}RKKLQ-Azo-^{30}DVHNF^{35}VAL	20	n.d.	n.d.
P7	^{25}RKKL-Azo-Q^{30}DVHNF^{35}VAL	140	32	27
P8	^{25}RK-Azo-KLQ^{30}DVHNF^{35}VAL	200	6	31
P9	^{25}R-Azo-KLQ^{30}DVHNF^{35}VAL	130	26	27
P10	^{25}RKKLQ^{30}DVHN-Azo-F^{35}VAL	7	n.d.	n.d.
P11	^{25}RKKLQ^{30}DVHNF^{35}V-Azo-AL	20	n.d.	n.d.
P12	^{25}RKKLQ^{30}DVHNF-Azo-AL	135	-	-

n.d. – not determined

Table S3. Photophysical properties of P1, P2, P4, P7, P8, P9, P12 in buffered solution. *cis*-PSS – *cis*-photostationary state at 340 nm after 30 min. *trans*-PSS – *trans*-photostationary state at 405 nm after 20 min.

**Peptide**	$t_{1/2}$ **(*cis*, 37 °C) [h]**	**after synthesis**	***cis*-PSS**	***trans*-PSS**
		***cis*:*trans* ratio**		
P1	90	5:95	86:14	19:81
P2	90	6:94	87:13	19:81
P4	97	3:97	82:18	24:76
P7	72	8:92	94:6	23:77
P8	89	6:94	85:15	23:77
P9	86	9:91	91:9	16:84
P12	63	4:96	90:10	19:81

Table S4. Fibrillization parameters of $PTH_{25–37}$, P1, P2, P4, P7, P8, and P9 in buffered solution. t_{lag} - lag time. t_{char} - characteristic time.

**Peptide**	t_{lag} **(*trans*) [h]**	t_{char} **(*trans*) [h]**	t_{lag} **(*cis*) [h]**	t_{char} **(*cis*) [h]**
PTH_{25-37}	7.770 ± 1.111	12.797 ±3.341	-	-
P1	0.392 ± 0.013	0.556 ± 0.008	9.759 ± 1.383	19.640 ±0.842
P2	0.482 ± 0.207	1.247 ± 0.208	13.052 ±1.336	17.867 ± 0.360
P4	1.917 ± 0.507	2.371 ± 0.433	29.525 ±0.558	42.444 ± 5.251
P7	0.381 ± 0.056	0.627 ± 0.078	8.014 ± 1.643	9.875 ± 1.881
P8	1.069 ± 0.096	1.356 ± 0.197	5.524 ±1.781	20.701 ± 4.415
P9	0.267 ± 0.019	0.653 ± 0.040	3.833 ± 0.458	4.188 ± 0.290

3. Peptide characterization after synthesis

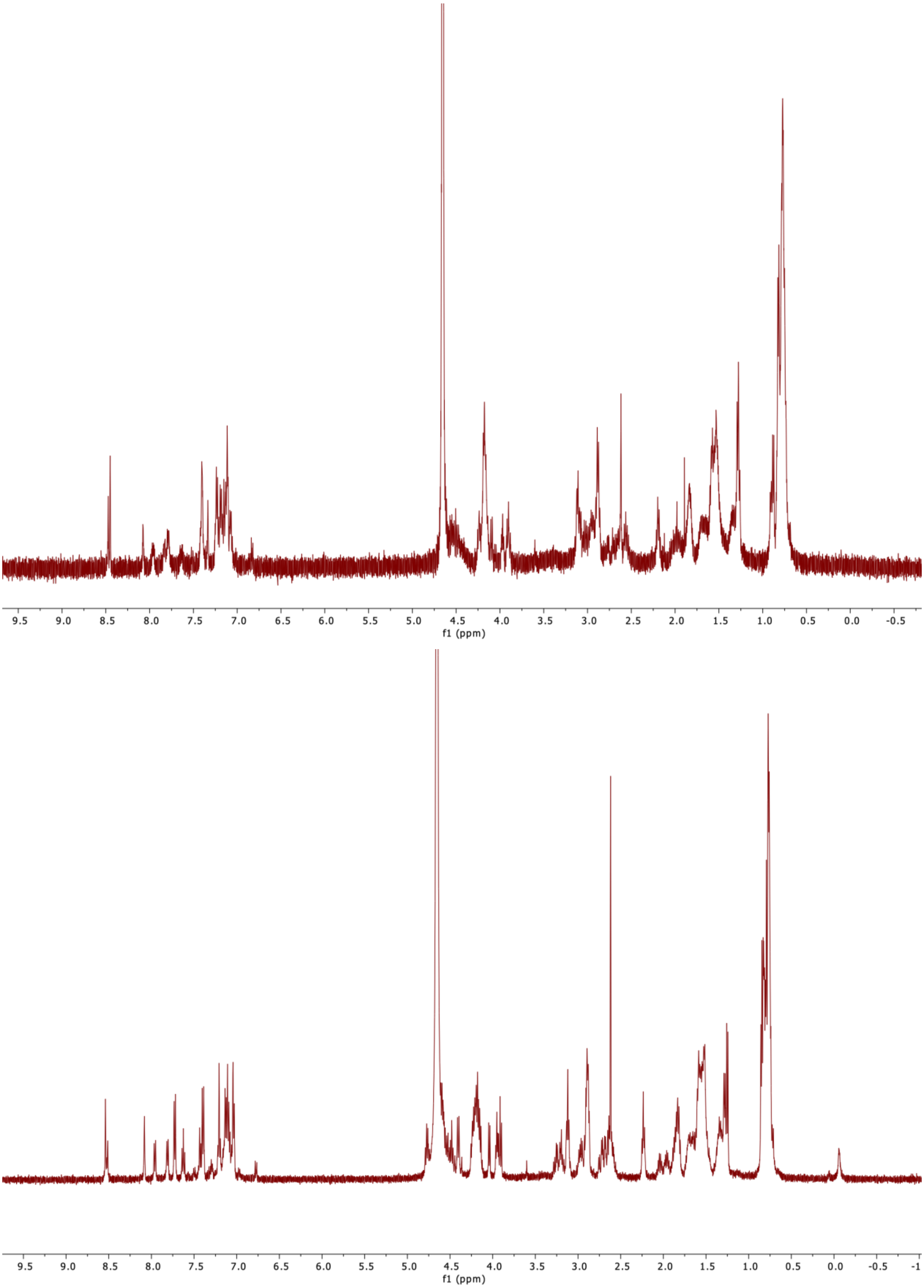

Figure S30: ^{1}H-NMR spectra (500 MHz, D_2O) of P1 (top, *trans*-isomer) and P2 (bottom, *trans*-isomer).[3]

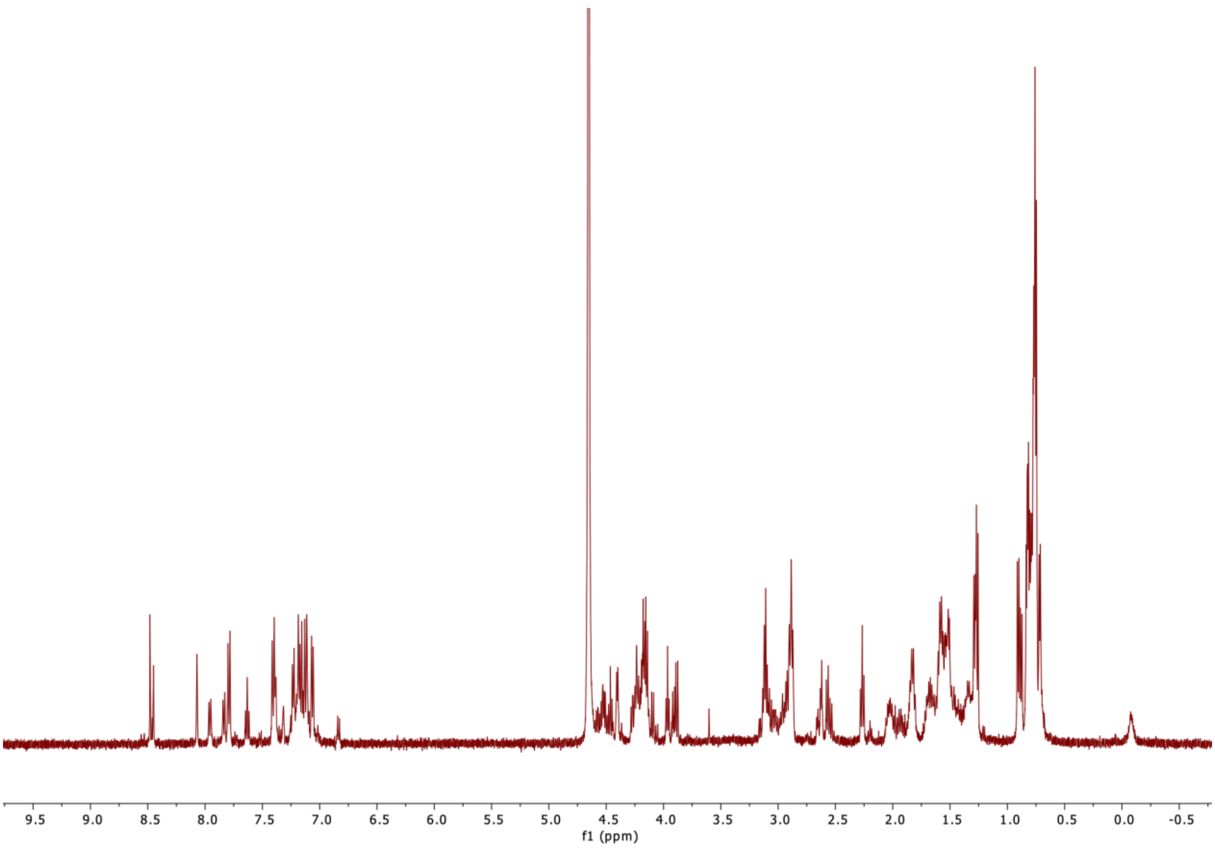

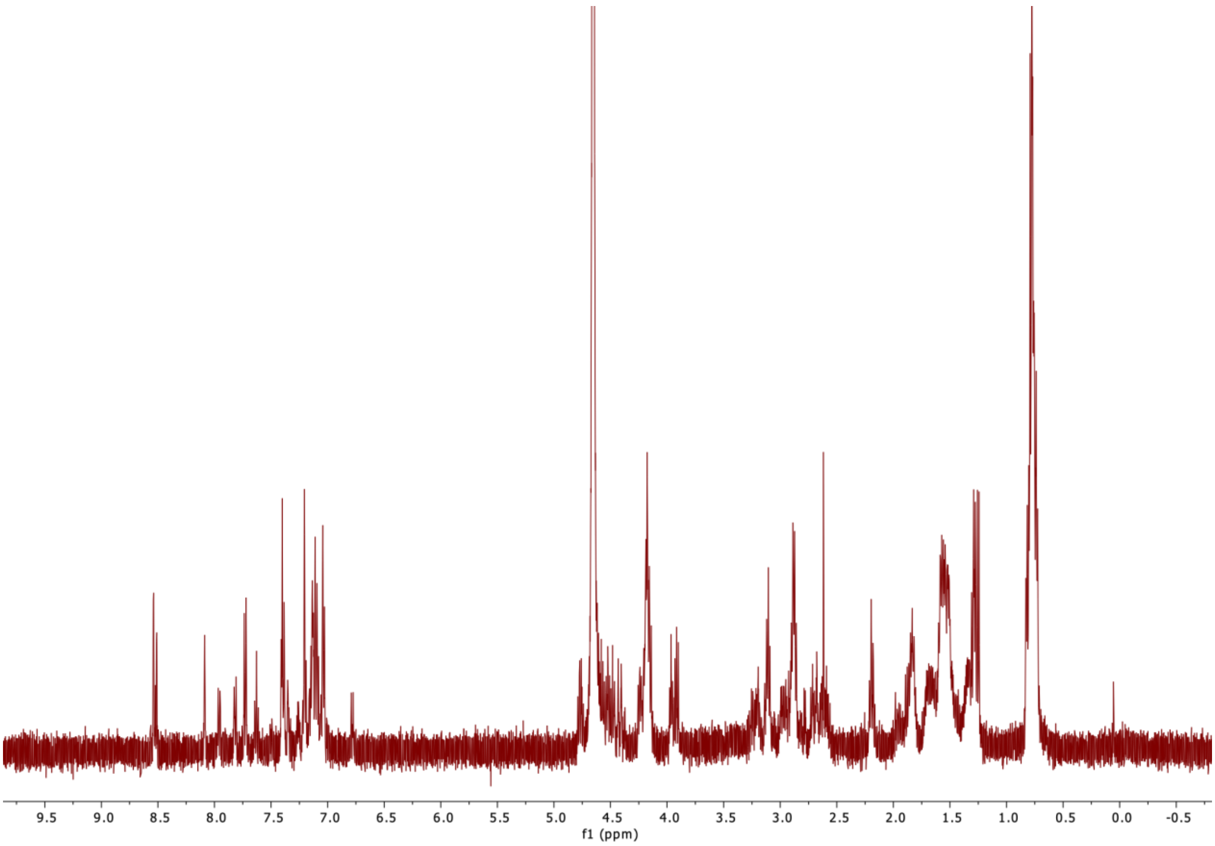

Figure S31: ^{1}H-NMR spectra (500 MHz, D_2O) of P3 (top, *trans*-isomer) and P4 (bottom, *trans*-isomer).[3]

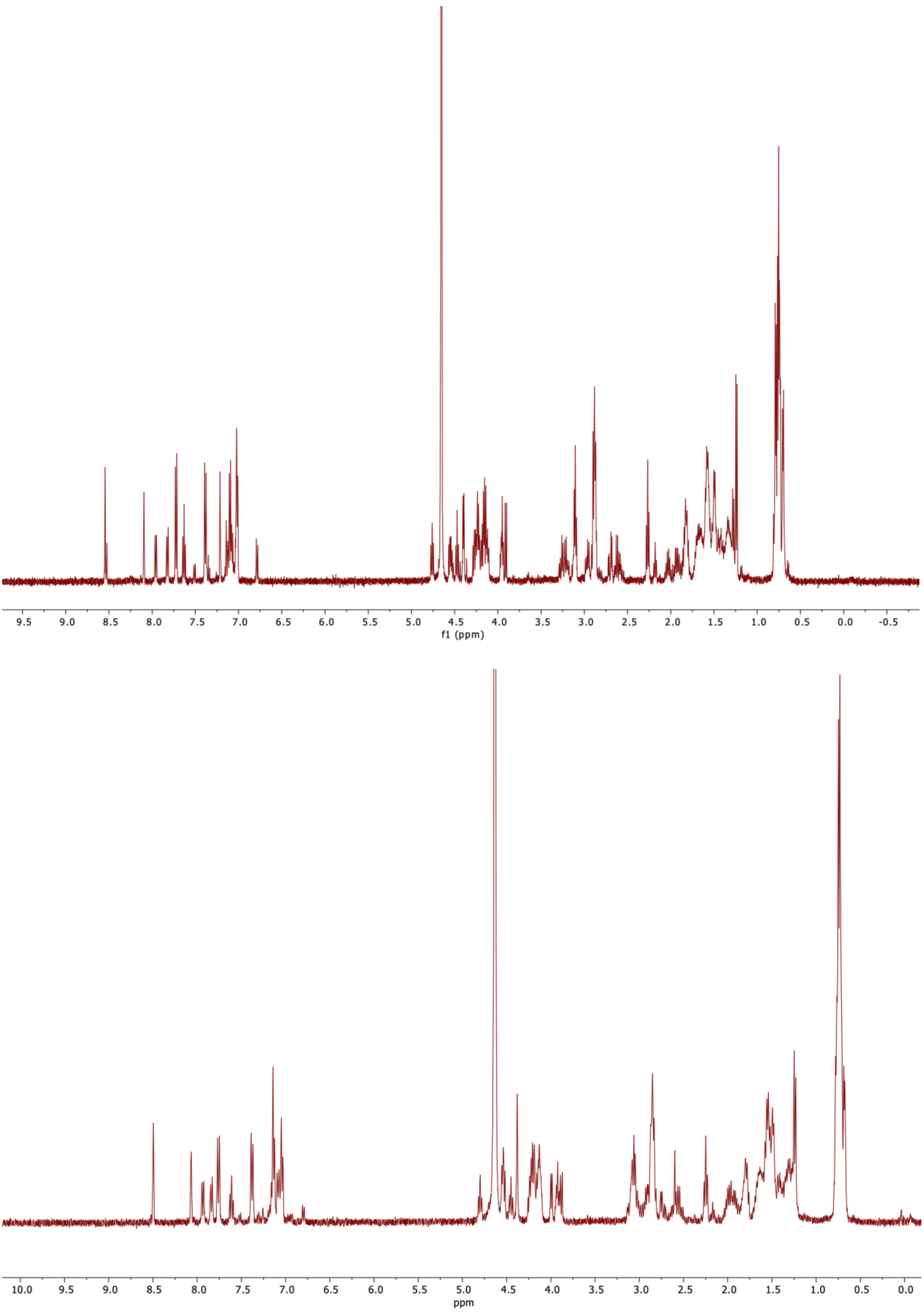

Figure S32: ^{1}H-NMR spectra (500 MHz, D_2O) of P5 (top, *trans*-isomer)[3] and P6 (bottom, *trans*-isomer).

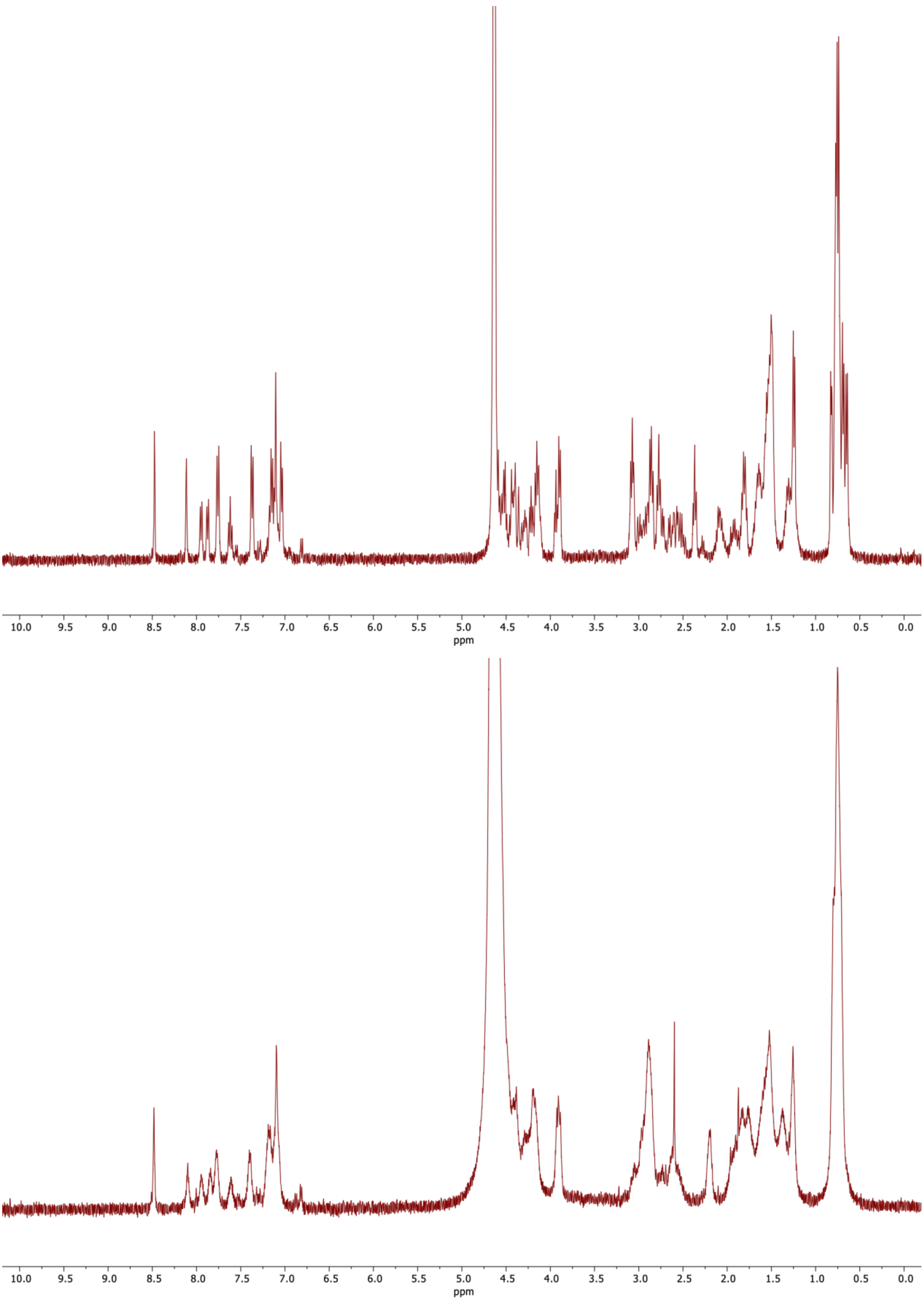

Figure S33: ^{1}H-NMR spectra (500 MHz, D_2O) of P7 (top, *trans*-isomer) and P8 (bottom, *trans*-isomer).

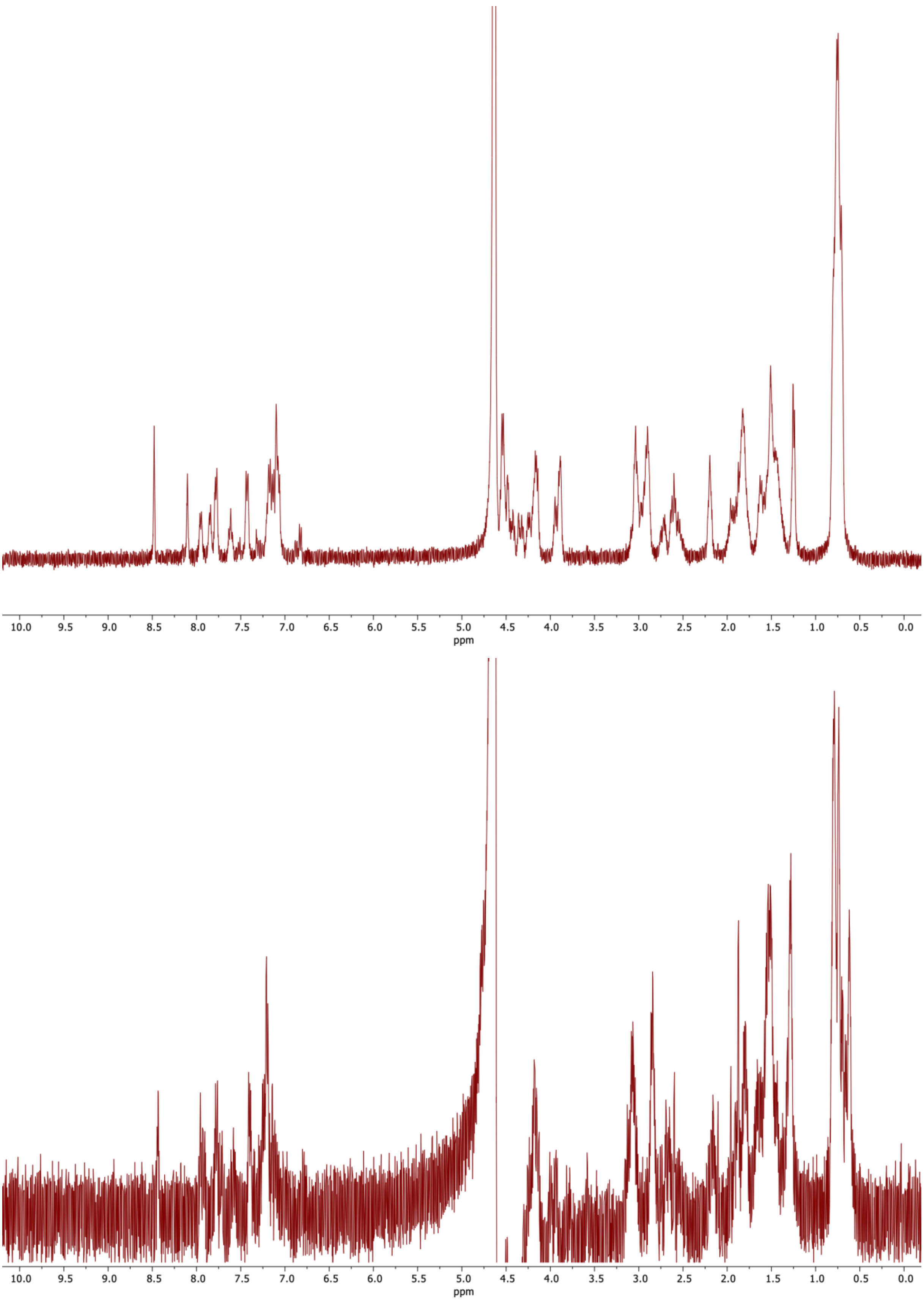

Figure S34: ^{1}H-NMR spectra (500 MHz, D_2O) of P9 (top, *trans*-isomer) and P10 (bottom, *trans*-isomer).

+

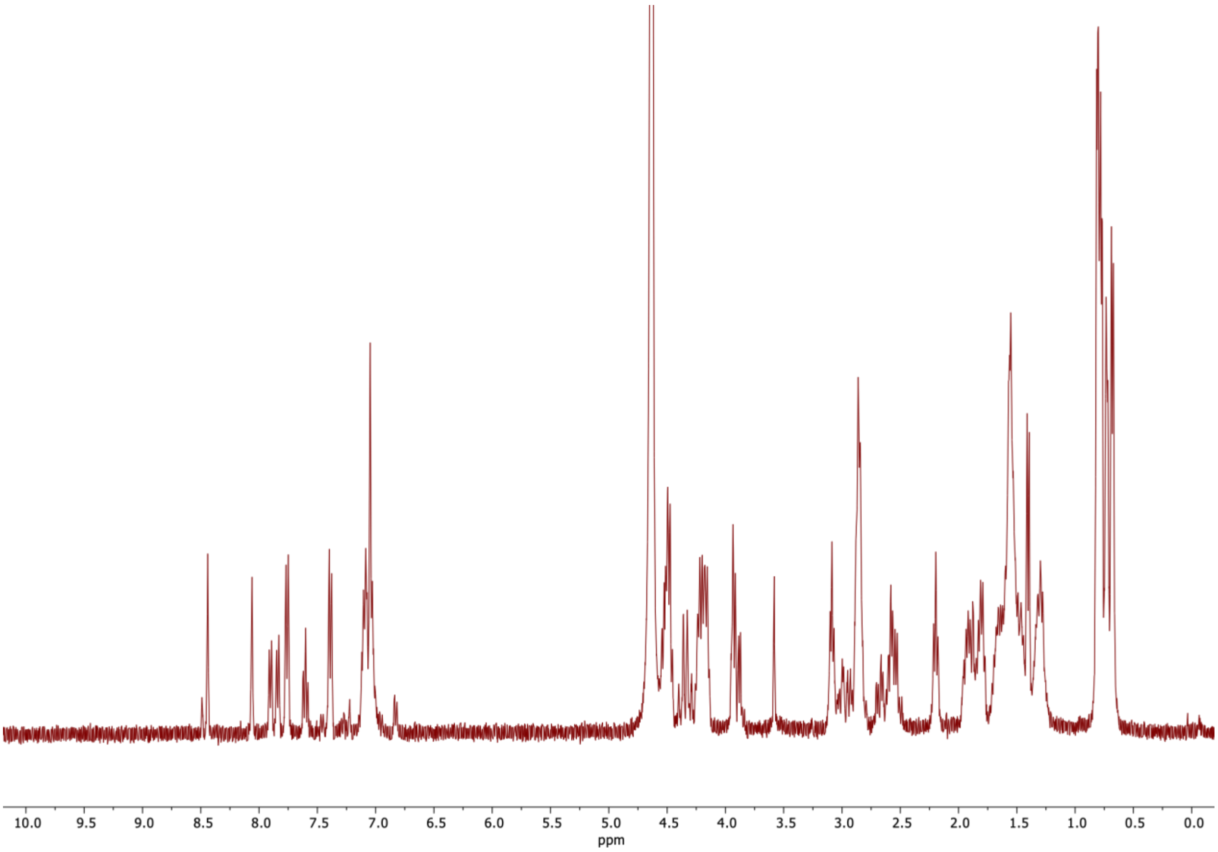

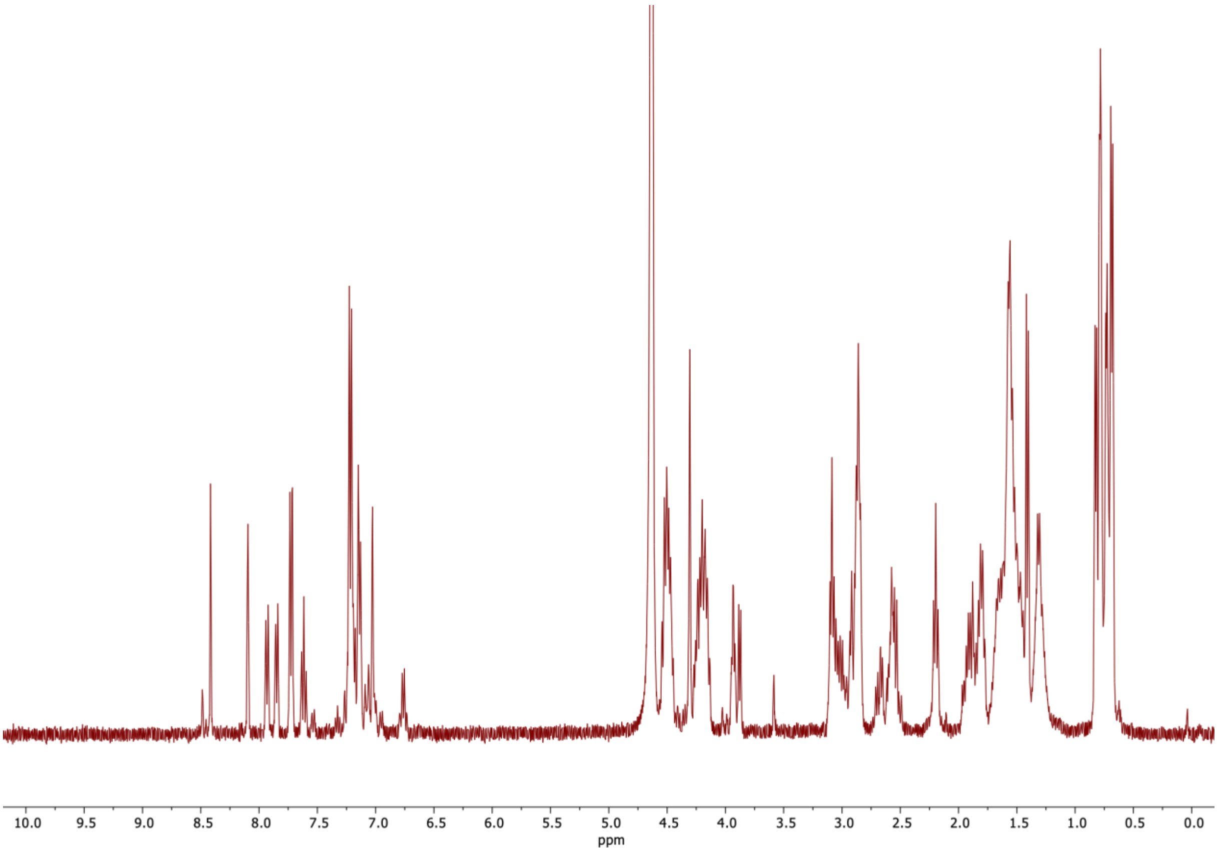

Figure S35: ^{1}H-NMR spectra (500 MHz, D_2O) of P11 (top, *trans*-isomer) and P12 (bottom, *trans*-isomer).

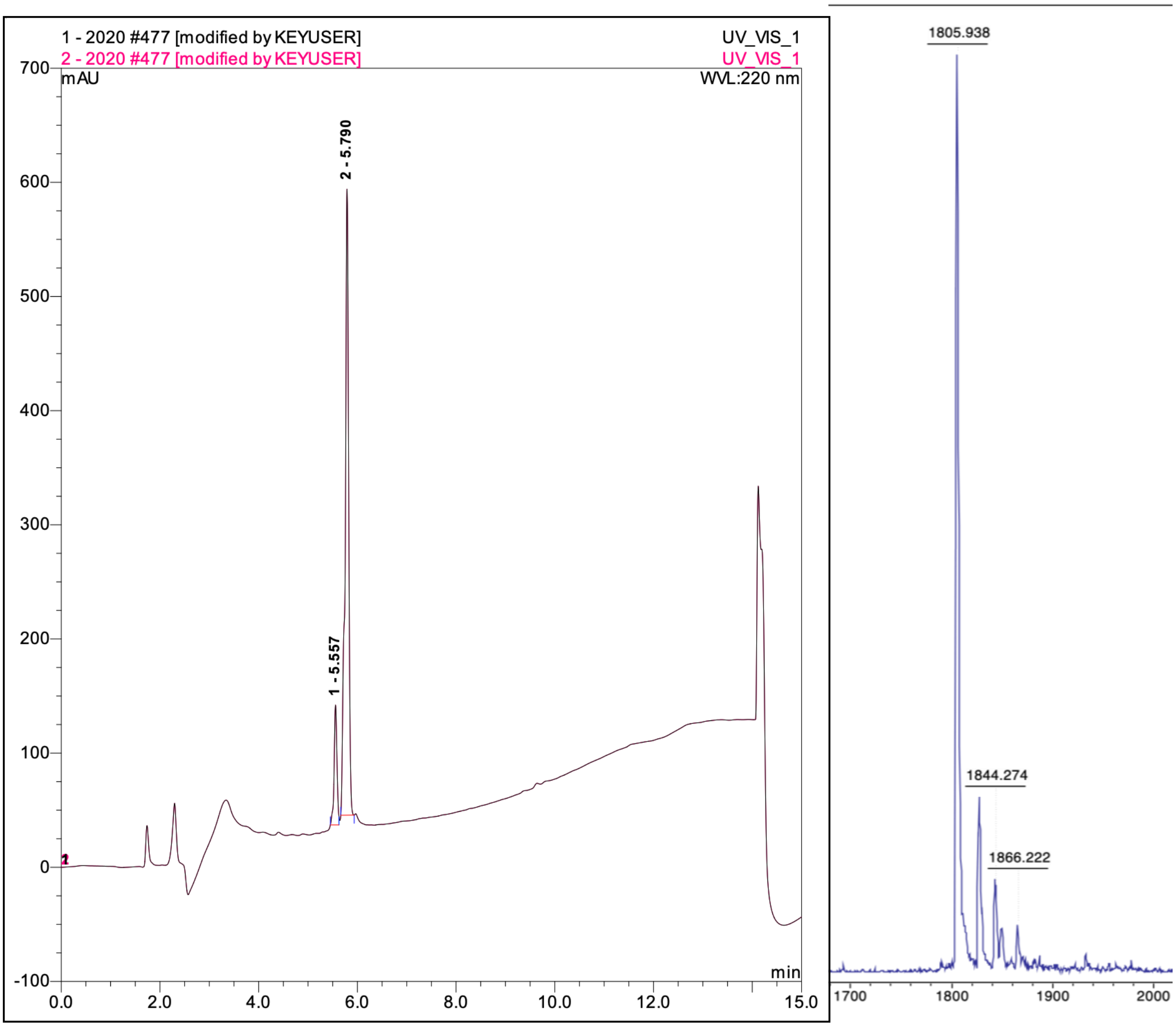

Figure S36: (**left**) HPLC-trace of P1 (*cis*- and *trans*-isomer between 5 and 6 min). (**right**) MALDI-spectrum of P1 (*m/z* calculated: 1805.00 found: 1805.938).[3]

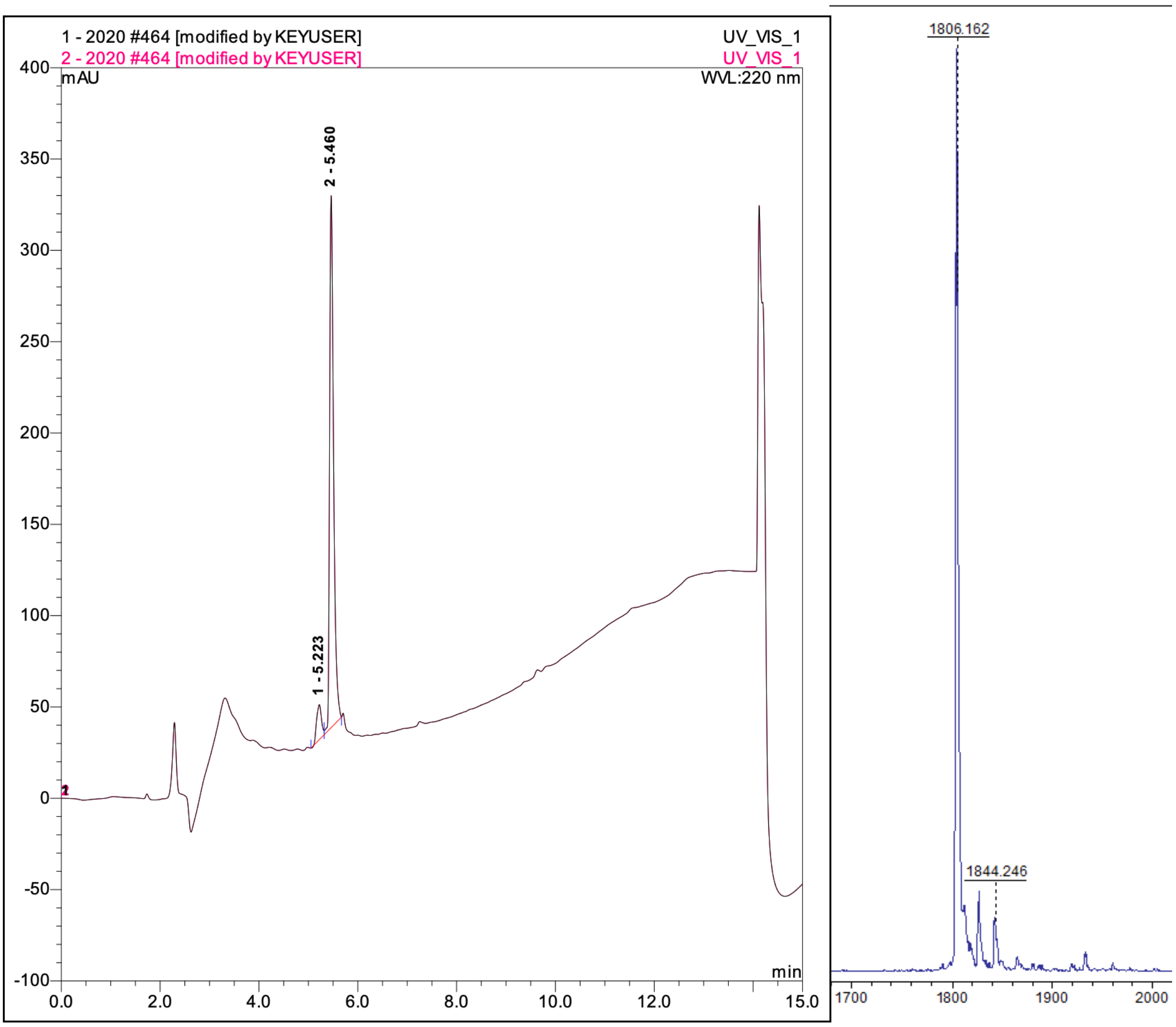

Figure S37: (**left**) HPLC-trace of P2 (*cis*- and *trans*-isomer between 5 and 6 min). (**right**) MALDI-spectrum of P2 (*m/z* calculated: 1805.00 found: 1806.162).[3]

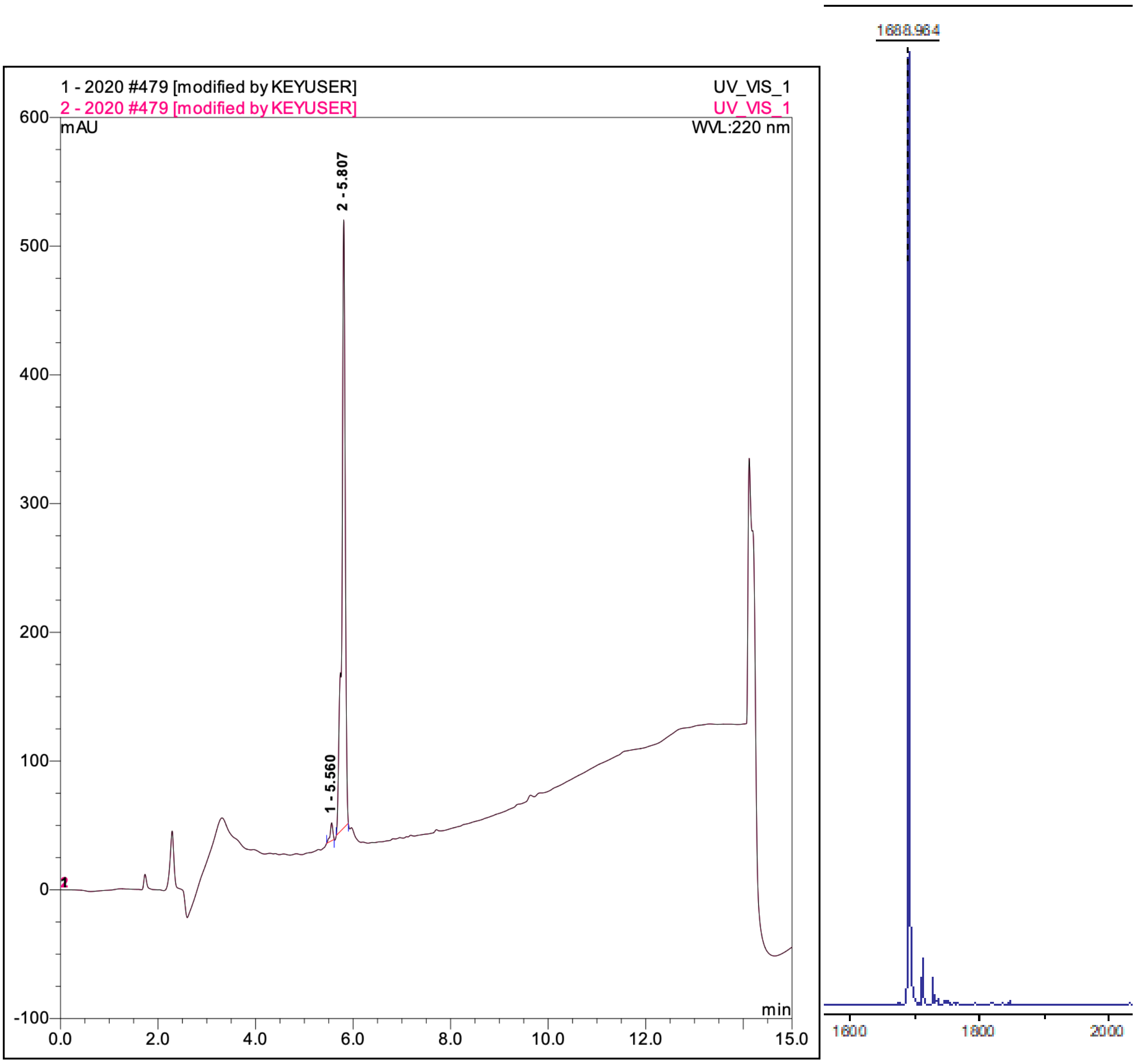

Figure S38: (**left**) HPLC-trace of P3 (*cis*- and *trans*-isomer between 5 and 6 min). (**right**) MALDI-spectrum of P3 (*m/z* calculated: 1689.97 found: 1688.964).[3]

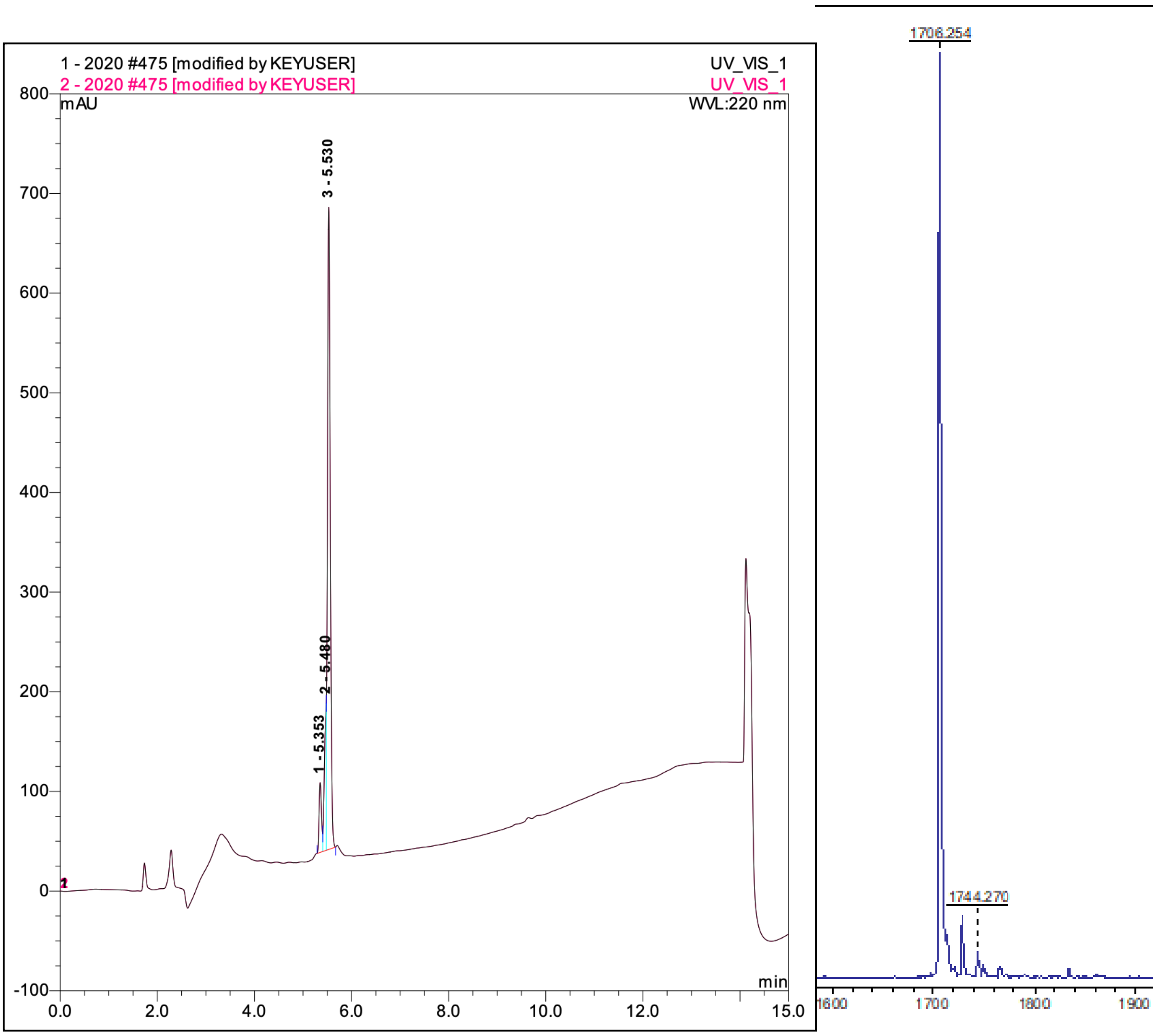

Figure S39: (**left**) HPLC-trace of P4 (*cis*- and *trans*-isomer between 5 and 6 min) (**right**) MALDI-spectrum of P4 (*m/z* calculated: 1705.93 found: 1706.254).[3]

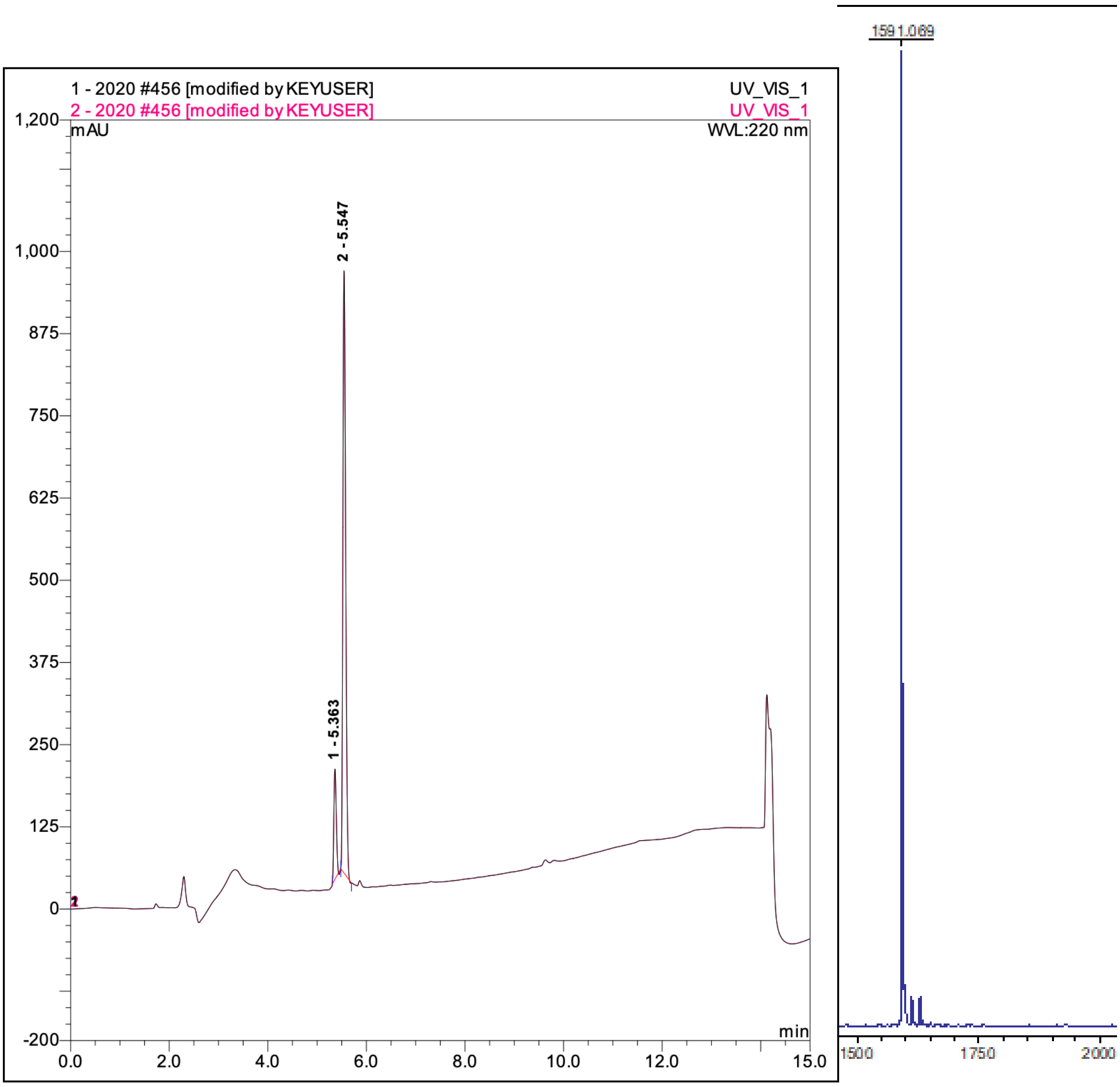

Figure S40: (**left**) HPLC-trace of P5 (*cis*- and *trans*-isomer between 5 and 6 min). (**right**) MALDI-spectrum of P5 (*m/z* calculated: 1590.90 found: 1591.069).[3]

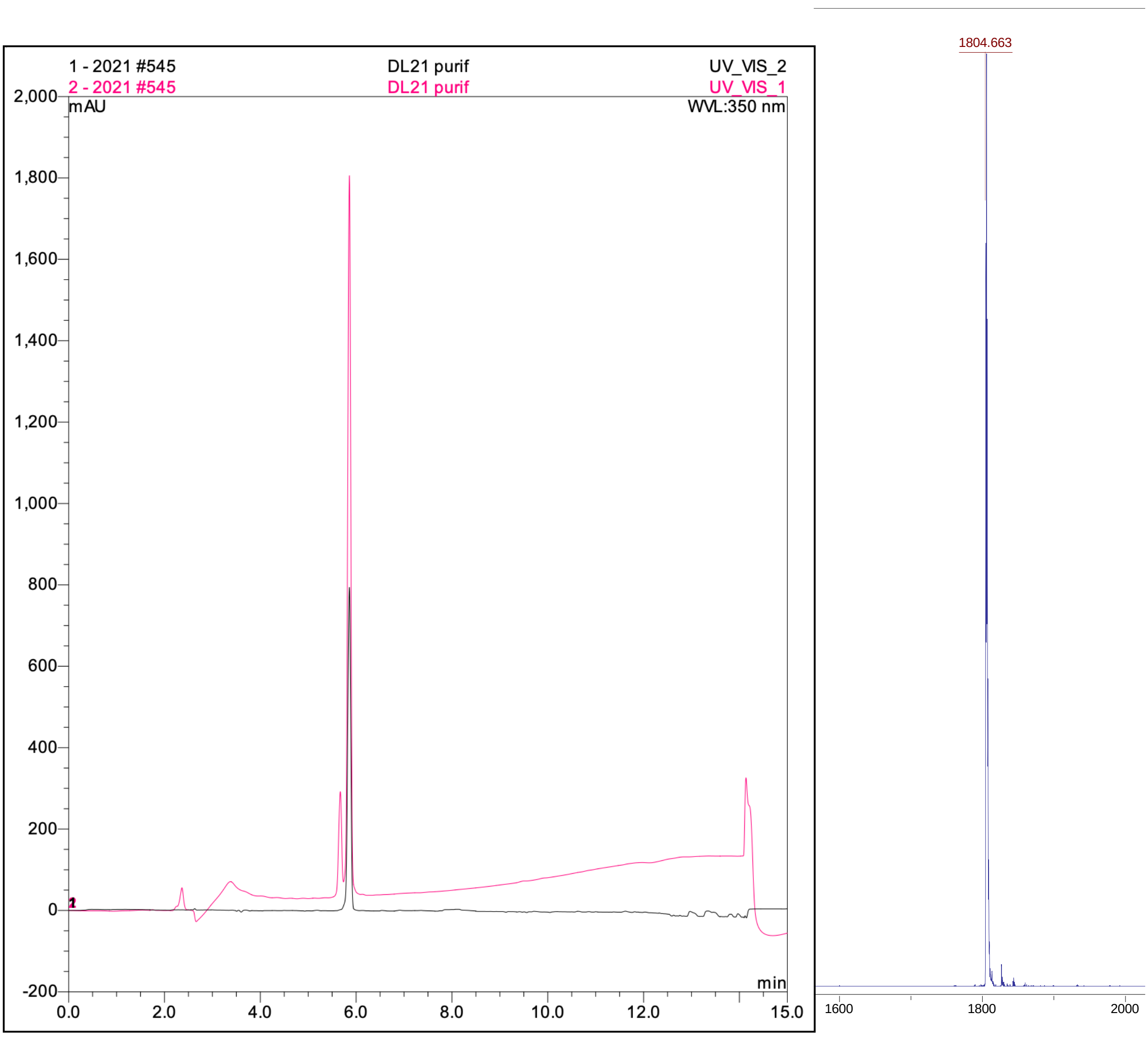

Figure S41: (**left**) HPLC-trace of P6 (*cis*- and *trans*-isomer between 5 and 6 min). (**right**) MALDI-spectrum of P6 (*m/z* calculated: 1805.00 found: 1804.663).

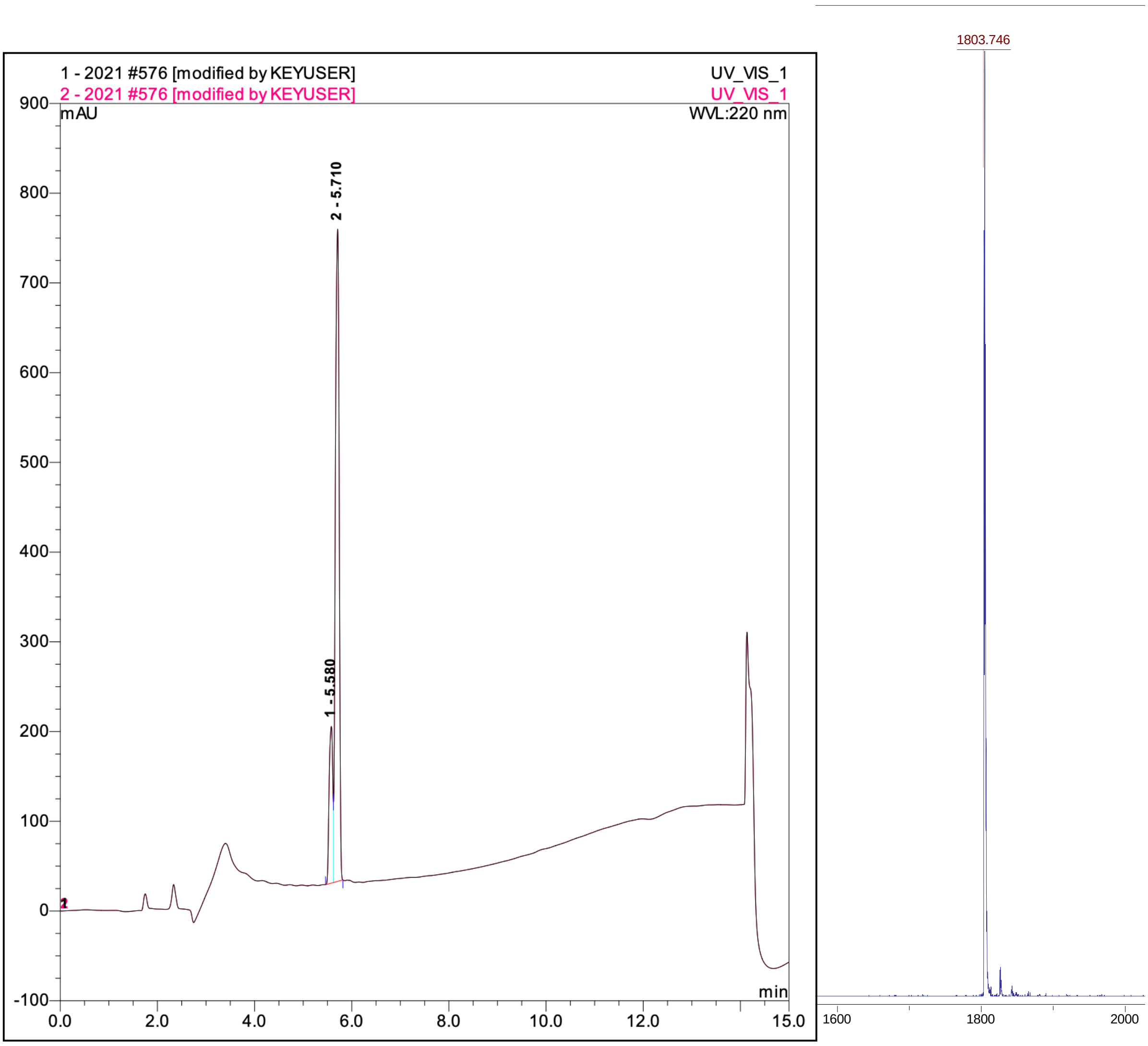

Figure S42: (**left**) HPLC-trace of P7 (*cis*- and *trans*-isomer between 5 and 6 min). (**right**) MALDI-spectrum of P7 (*m/z* calculated: 1805.00 found: 1803.746).

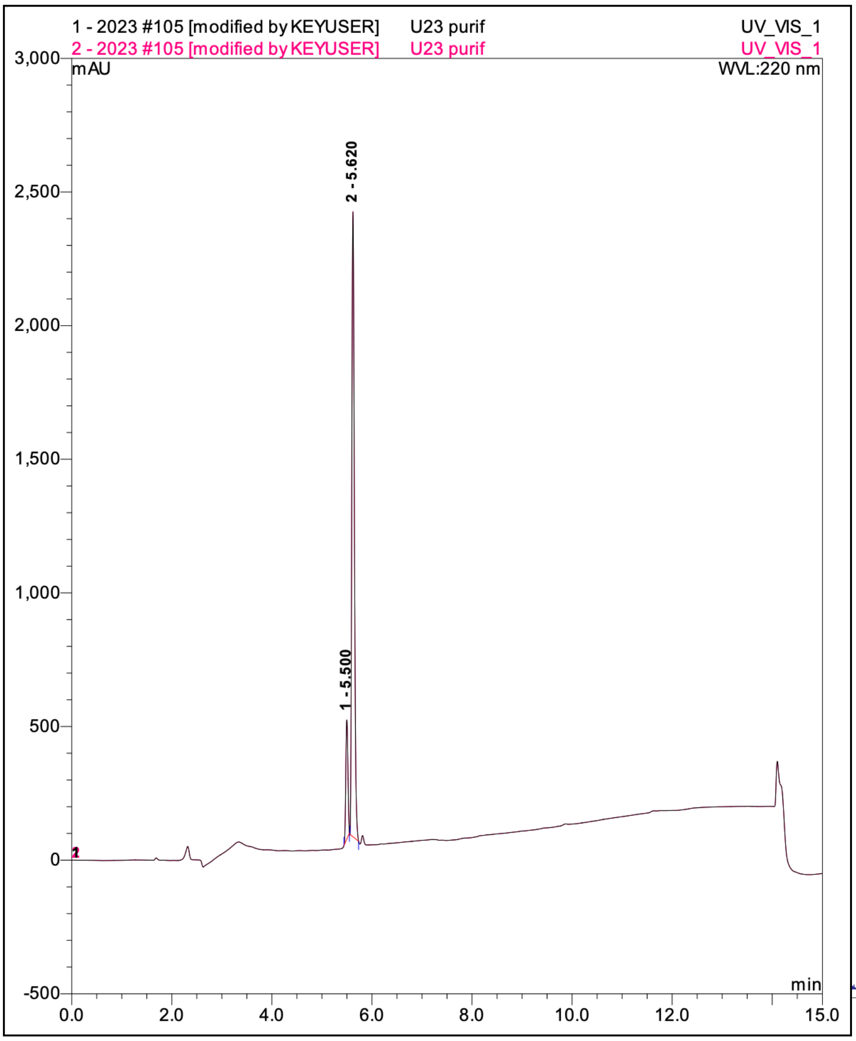

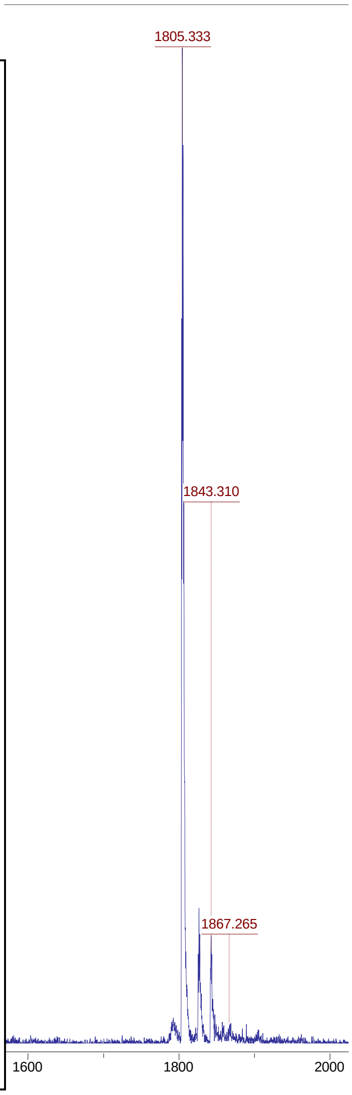

Figure S43: (**left**) HPLC-trace of P8 (*cis*- and *trans*-isomer between 5 and 6 min). (**right**) MALDI-spectrum of P8 (*m/z* calculated: 1805.00 found: 1805.333).

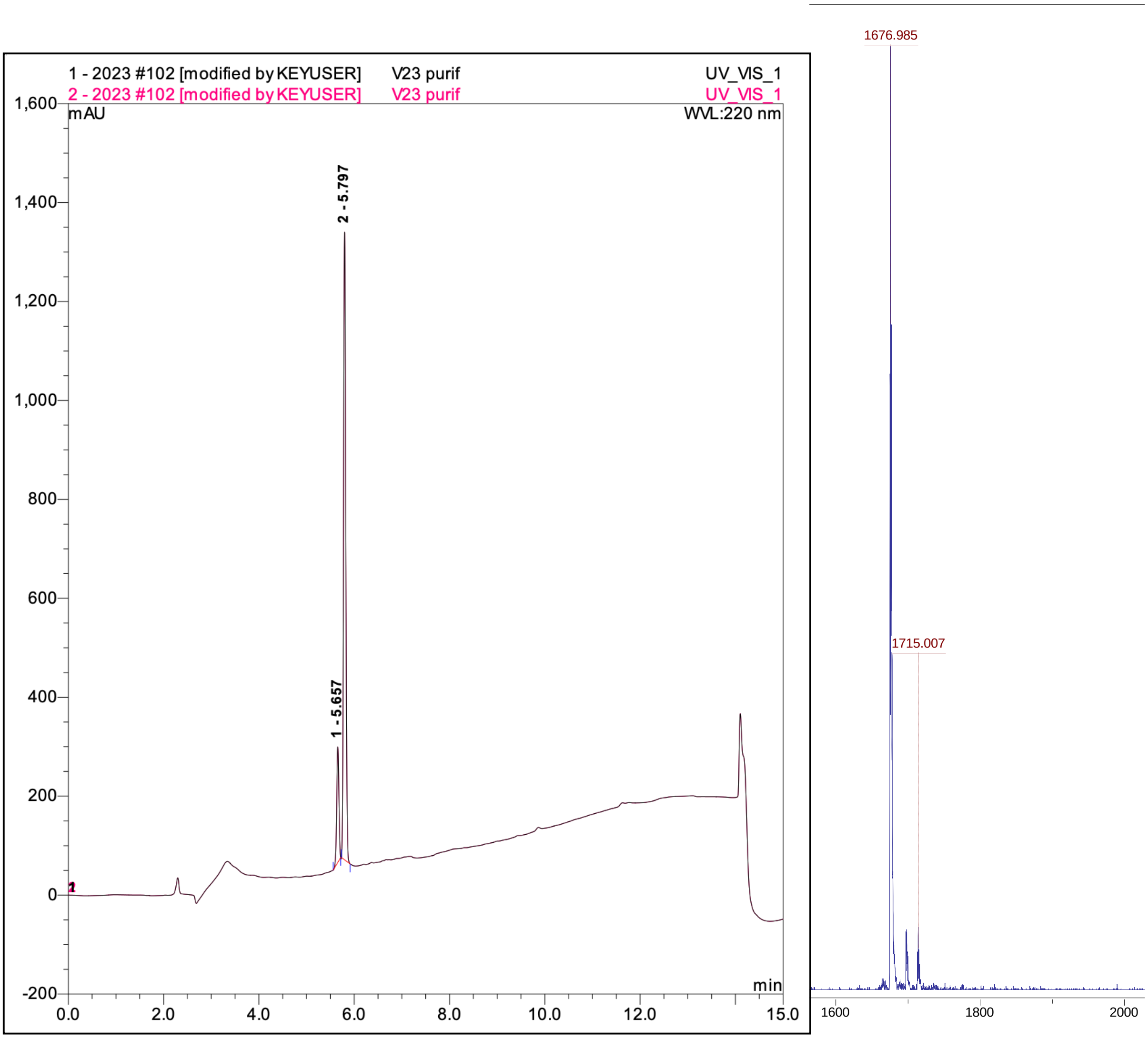

Figure S44: (**left**) HPLC-trace of P9 (*cis*- and *trans*-isomer between 5 and 6 min). (**right**) MALDI-spectrum of P9 (*m/z* calculated: 1676.90 found: 1676.985).

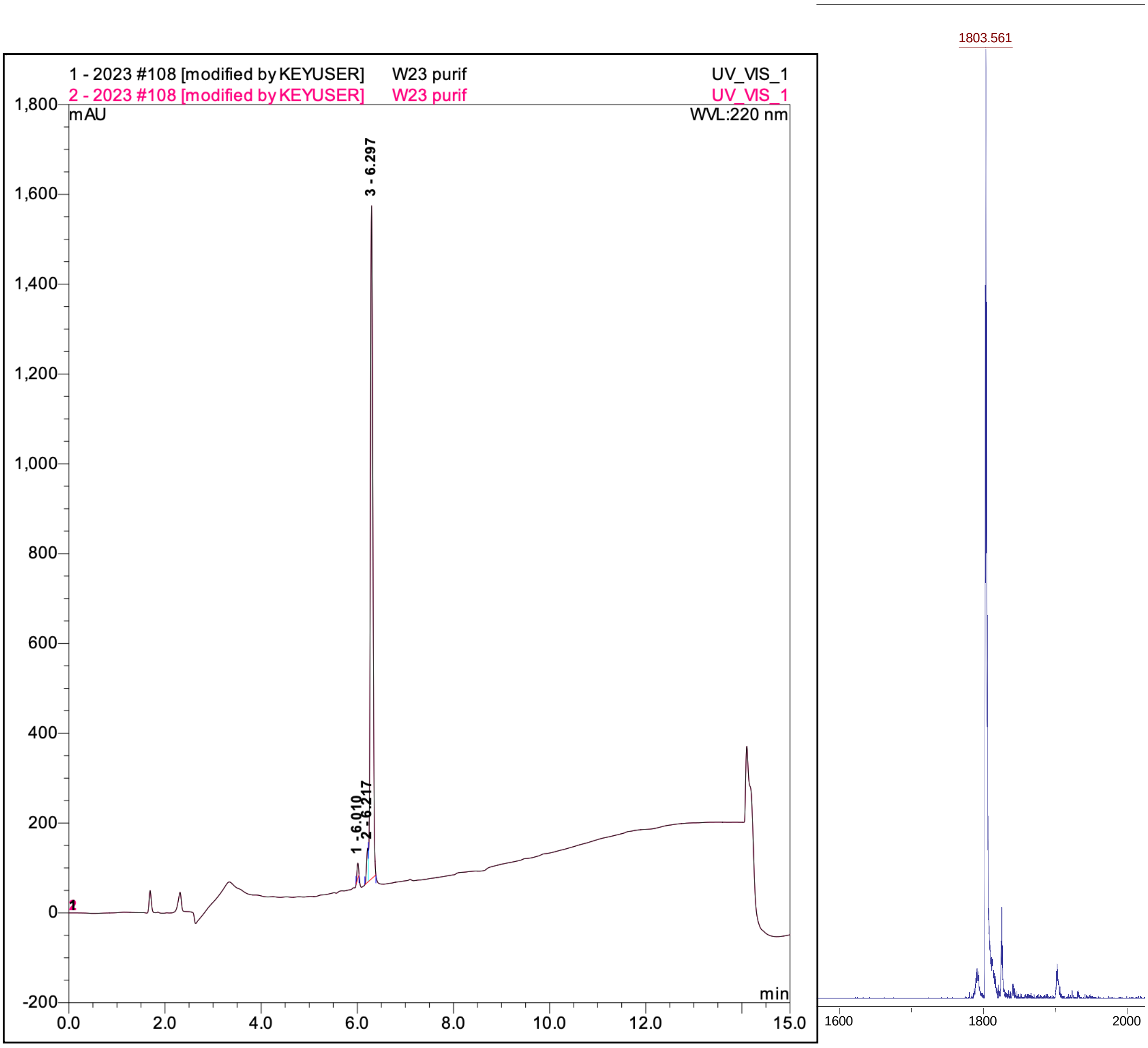

Figure S45: (**left**) HPLC-trace of P10 (*cis*- and *trans*-isomer between 6 and 7 min). (**right**) MALDI-spectrum of P10 (*m/z* calculated: 1805.00 found: 1803.561).

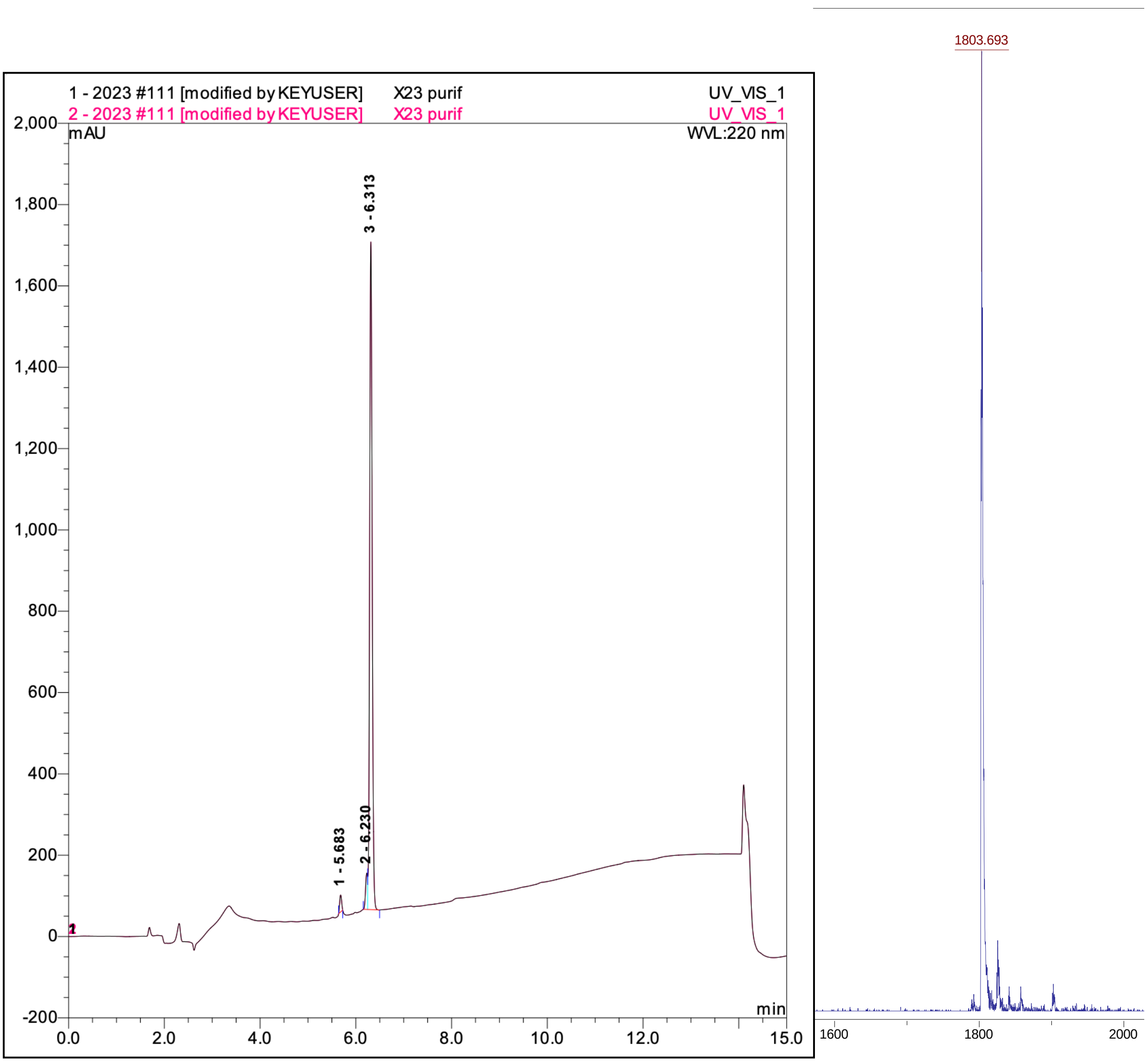

Figure S46: (**left**) HPLC-trace of P11 (*cis*- and *trans*-isomer between 6 and 7 min). (**right**) MALDI-spectrum of P11 (*m/z* calculated: 1805.00 found: 1803.693).

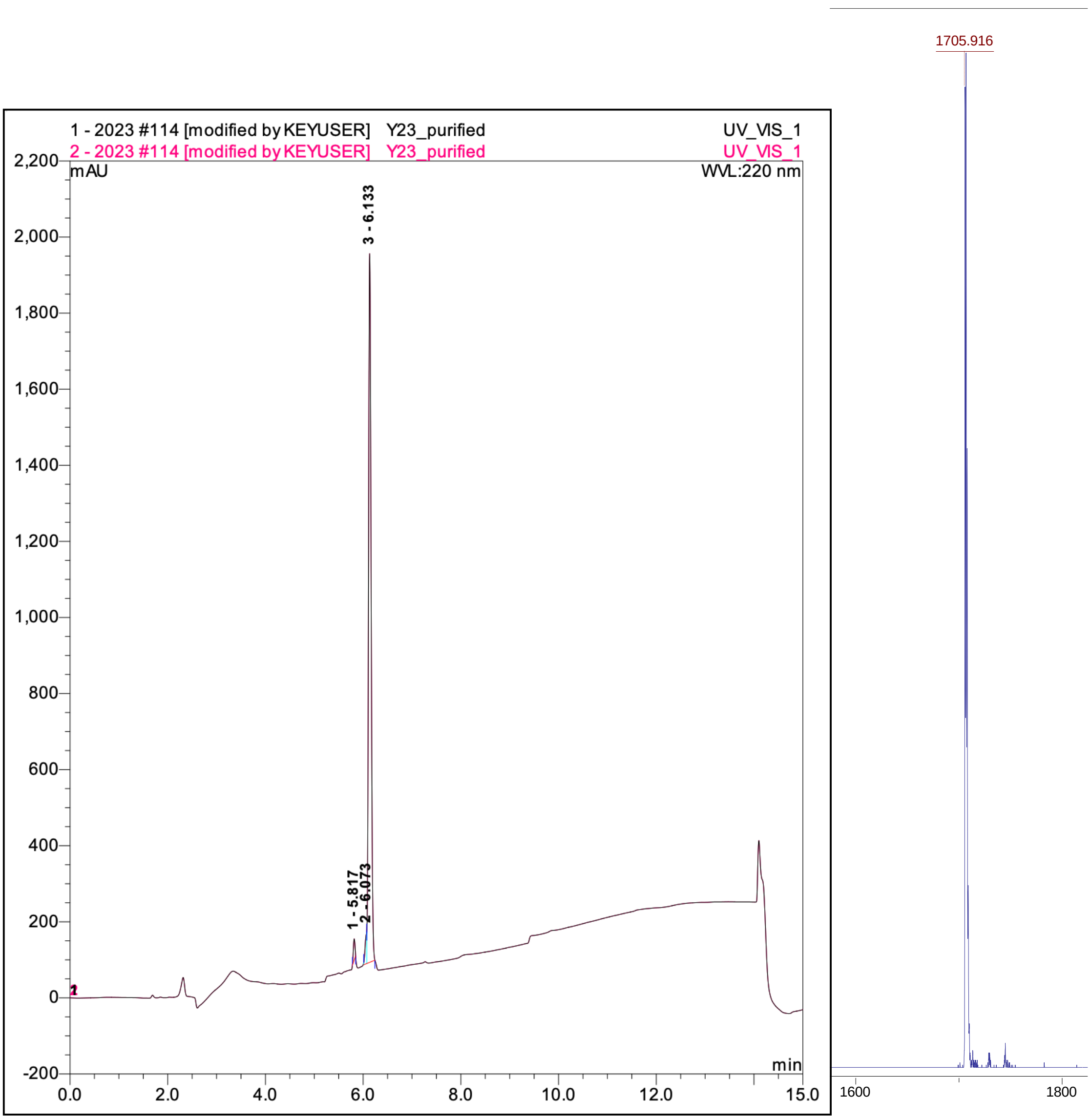

Figure S47: (**left**) HPLC-trace of P12 (*cis*- and *trans*-isomer between 5.5 and 6.5 min) (**right**) MALDI-spectrum of P12 (*m/z* calculated: 1705.93 found: 1705.916).

References

(1) Jurrus, E.; Engel, D.; Star, K.; Monson, K.; Brandi, J.; Felberg, L. E.; Brookes, D. H.; Wilson, L.; Chen, J.; Liles, K. Improvements to the APBS biomolecular solvation software suite. *Protein Sci.* **2018**, *27* (1), 112-128. DOI: 10.1002/pro.3280.
(2) Schrödinger, L. The PyMOL Molecular Graphics System, Version 1.8. In *(No Title)*, 2015.
(3) Paschold, A.; Voigt, B.; Hause, G.; Kohlmann, T.; Rothemund, S.; Binder, W. H. Modulating the Fibrillization of Parathyroid-Hormone (PTH) Peptides: Azo-Switches as Reversible and Catalytic Entities. *Biomedicines* **2022**, *10* (7), 1512. DOI: 10.3390/biomedicines10071512.
